# *Anaplasma phagocytophilum* modifies tick cell microRNA expression and upregulates isc-mir-79 to facilitate infection by targeting the Roundabout protein 2 pathway

**DOI:** 10.1038/s41598-019-45658-2

**Published:** 2019-06-24

**Authors:** Sara Artigas-Jerónimo, Pilar Alberdi, Margarita Villar Rayo, Alejandro Cabezas-Cruz, Pedro J. Espinosa Prados, Lourdes Mateos-Hernández, José de la Fuente

**Affiliations:** 1 SaBio. Instituto de Investigación en Recursos Cinegéticos IREC-CSIC-UCLM-JCCM, Ronda de Toledo s/n, 13005 Ciudad, Real Spain; 2 UMR BIPAR, INRA, Ecole Nationale Vétérinaire d’Alfort, ANSES, Université Paris-Est, 94700 Maisons-Alfort, France; 3 Department of Veterinary Pathobiology, Center for Veterinary Health Sciences, Oklahoma State University, Stillwater, OK 74078 USA

**Keywords:** Molecular biology, Non-coding RNAs, Immunology

## Abstract

The microRNAs (miRNAs) are a class of small noncoding RNAs that have important regulatory roles in multicellular organisms including innate and adaptive immune pathways to control bacterial, parasite and viral infections, and pathogens could modify host miRNA profile to facilitate infection and multiplication. Therefore, understanding the function of host miRNAs in response to pathogen infection is relevant to characterize host-pathogen molecular interactions and to provide new targets for effective new interventions for the control infectious diseases. The objective of this study was to characterize the dynamics and functional significance of the miRNA response of the tick vector *Ixodes scapularis* in response to *Anaplasma phagocytophilum* infection, the causative agent of human and animal granulocytic anaplasmosis. To address this objective, the composition of tick miRNAs, functional annotation, and expression profiling was characterized using high throughout RNA sequencing in uninfected and *A. phagocytophilum*-infected *I. scapularis* ISE6 tick cells, a model for tick hemocytes involved in pathogen infection. The results provided new evidences on the role of tick miRNA during pathogen infection, and showed that *A. phagocytophilum* modifies *I. scapularis* tick cell miRNA profile and upregulates isc-mir-79 to facilitate infection by targeting the Roundabout protein 2 (Robo2) pathway. Furthermore, these results suggested new targets for interventions to control pathogen infection in ticks.

## Introduction

Ticks are bloodsucking arthropod ectoparasites that transmit pathogens, which cause diseases in humans and animals with growing incidence worldwide. *Ixodes scapularis* is the vector of pathogens such as *Borrelia burgdorferi* and *Anaplasma phagocytophilum*, the causative agents of Lyme disease and human granulocytic anaplasmosis (HGA), respectively^1,2^.

*Anaplasma phagocytophilum* is an obligate intracellular bacterium that develops within membrane-bound inclusions in the cytoplasm of vertebrate host neutrophils and tick cells in several tissues such as midguts, hemocytes and salivary glands from where pathogen transmission occurs^3–5^. The application of latest omics technologies has advanced our understanding of the molecular mechanisms involved in the *A. phagocytophilum* interactions with tick and vertebrate hosts, showing that it has evolved through dynamic processes involving genetic traits of the pathogen, vectors and hosts to guarantee pathogen infection, development, persistence, and survival^6–8^. Recently, it was shown the role of RNA interference (RNAi) in tick feeding^9^ and response to virus infection^10,11^. However, despite the relevant role of microRNAs (miRNAs) during pathogen infection^12–16^ information is not available for this process in the tick vectors.

Host miRNAs are short non-coding RNA sequences that suppress gene expression by mostly binding to 3′ untranslated regions (3′-UTRs) of target mRNAs to cause translation repression or mRNA cleavage and degradation^17,18^. However, miRNAs have been also reported to regulate mRNA translation and stability after binding to recognition sites in the coding region and 5′-UTR^18^. In this way, miRNAs have a function in development, differentiation, apoptosis and immune response^13^. Host miRNAs also regulate proteins involved in innate and adaptive immune pathways to control bacterial, parasite and viral infections, and pathogens could modify host miRNA profile to facilitate infection and multiplication^12–16^. Based on their function, miRNAs have been used as biomarkers and potential interventions for the control of pathologies such as malignant, cardiovascular and infectious diseases^12,13^.

Therefore, understanding the function of host miRNAs in response to pathogen infection is relevant to characterize host-pathogen molecular interactions and provide new targets for effective interventions to control infectious diseases. The objective of this study was to characterize the dynamics and functional significance of the miRNAome of the tick vector *I. scapularis* in response to *A. phagocytophilum* infection. To address this objective, the composition of tick miRNAs, functional annotation, and expression profiling was characterized using high throughout RNA sequencing (RNA-seq) in uninfected and *A. phagocytophilum*-infected *I. scapularis* tick ISE6 cells, a model for tick hemocytes^19,20^. The results showed that *A. phagocytophilum* modifies *I. scapularis* tick cell miRNA profile and upregulates isc-mir-79 to facilitate infection by targeting the Roundabout protein 2 (Robo2) pathway.

## Results

### The miRNA profile changes in response to A. phagocytophilum infection of tick vector cells

**Figure 1:**
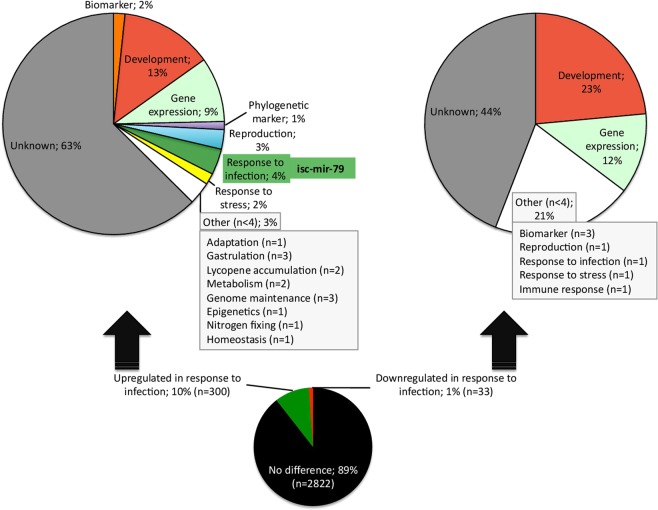
Characterization of the miRNA profile in response to *A. phagocytophilum* infection of tick ISE6 cells. (**A**) Total of 3,155 miRNAs were identified after miRNA-seq. Of them, 300 and 33 were upregulated and downregulated in response to *A. phagocytophilum* infection, respectively. Differentially expressed miRNAs (p < 0.05, q < 0.05) were functionally annotated based on the previously published information for homologous miRNAs in ticks and other species. Of the 16 miRNAs within the response to infection category and upregulated in response to infection, the isc-mir-79 (infected/uninfected ratio = 4.4; p = 0.002, q = 0.047) was selected for further analysis.

An analytical pipeline was developed based on the approach proposed by Kuhn *et al*.^21^ to provide insights into the profile and functional characterization of tick vector cell miRNAs in response to bacterial infection using the *I. scapularis*-*A. phagocytophilum* model (Supplementary Fig. S1). The miRNA sequencing (miRNA-seq) identified a total of 3,155 miRNAs of which 32 were previously reported in *I. scapularis* (Supplementary Table S1). Of the identified miRNAs, 300 and 33 were up and downregulated in response to *A. phagocytophilum* infection, respectively (Fig. 1 and Supplementary Table S1).

The putative functional annotation of the differentially expressed miRNAs based on homologs described in ticks and other species showed that most of them still have an unknown function, followed by annotations in the development and gene expression categories (Fig. 1). Some functional annotations were more abundant or only represented in upregulated miRNAs, including the response to infection, which was selected for further characterization (Fig. 1).

**Figure 2:**
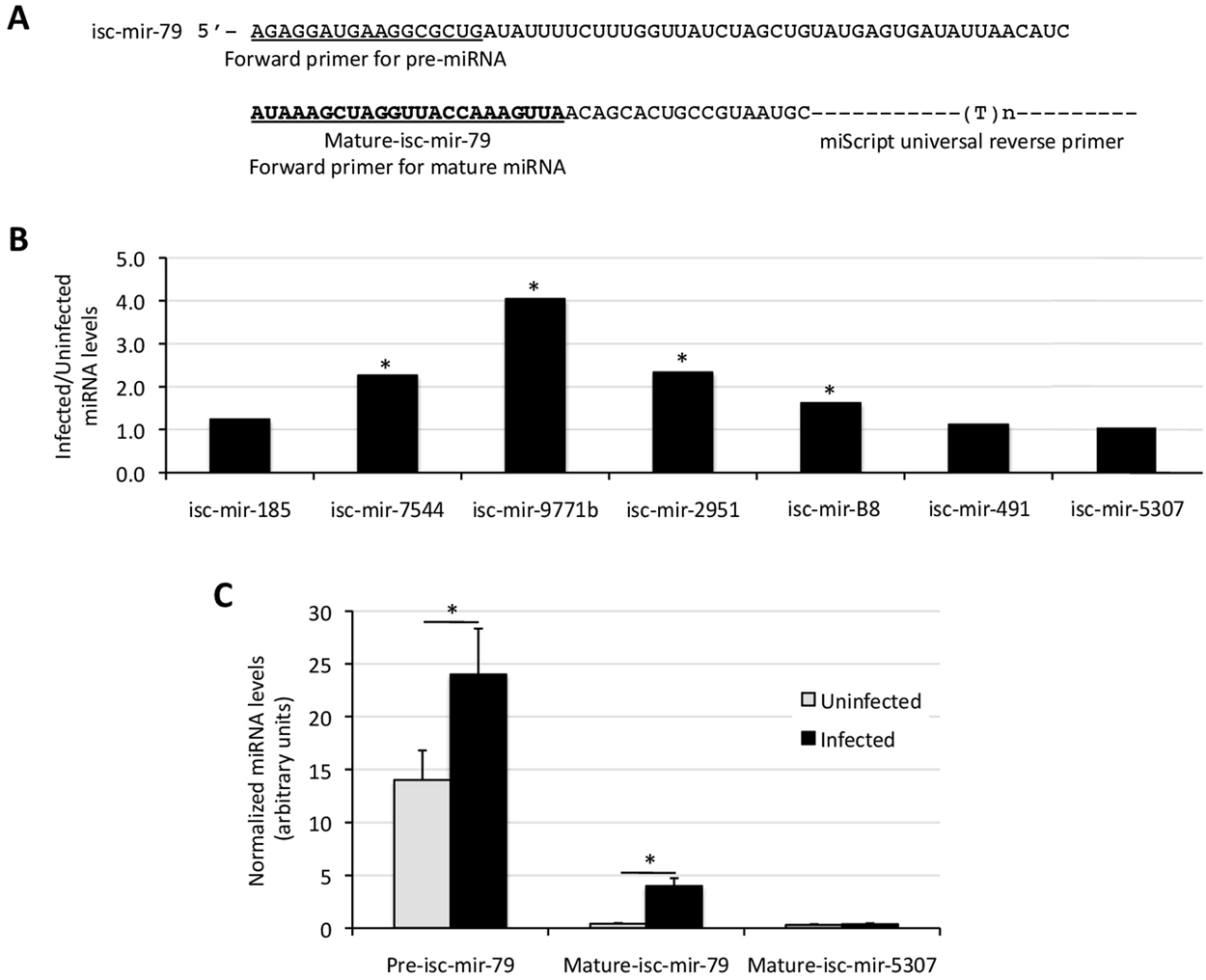
Validation of miRNA levels in response to *A. phagocytophilum* infection of tick ISE6 cells. (**A**) Forward primers were designed for selected miRNAs upregulated in response to infection, and used to characterize by qRT-PCR the levels of pre and mature isc-mir-79 (shown as example), isc-mir-185, isc-mir-7544, isc-mir-9771b, isc-mir-2951, isc-mir-B8, isc-mir-491, and isc-mir-5307 using the miScript universal reverse primer. The mature-isc-mir-5037, which was not differentially regulated in response to infection, was used as control miRNA. The miRNA levels were characterized in uninfected and *A. phagocytophilum*-infected tick cells and shown as infected/uninfected ratio (**B**) or Ave + S.D. for pre and mature isc-mir-79, and isc-mir-5307 levels (**C**). The miRNA levels were normalized against tick *rps4*. The normalized Ct values were compared between groups by Student’s t-test with unequal variance (*p < 0.05; N = 3 biological replicates).

To validate the results of the miRNA-seq quantitative analysis, the levels of selected miRNAs upregulated in response to infection, isc-mir-79, isc-mir-185, isc-mir-7544, isc-mir-9771b, isc-mir-2951, isc-mir-B8, and isc-mir-491 were characterized by qRT-PCR (Fig. 2A–C). The mature-isc-mir-5037, which was not differentially regulated in response to infection, was used as control. The results corroborated the miRNA-seq results for most (6/8, 75%) miRNAs in response to infection (Figs. 2B,C). The isc-mir-79 (infected/uninfected ratio = 4.4; p = 0.002, q = 0.047), which was annotated in the response to infection biological process (Fig. 1) and previously described in *I. scapularis* as encoded in a non-protein coding region of locus DS885551 (https://www.vectorbase.org/Ixodes_scapularis/Gene/Summary?g=ISCW025234;r=DS885551:436490-436590;t=ISCW025234-RA) (Table S1) was selected for additional studies including demonstration of upregulation in response to infection for both precursor (pre) and mature miRNAs (Fig. 2C).

### The predicted mRNA targets for differentially regulated miRNAs affect multiple biological processes in response to A. phagocytophilum infection of tick vector cells

Putative mRNA targets were predicted for miRNAs downregulated (Supplementary Fig. S2A) and upregulated (Supplementary Fig. S2B) in response to *A. phagocytophilum* infection, and functionally annotated by biological processes in which they are implicated (see Supplementary Table S2 for detailed annotations). The results showed that miRNA putative gene targets are involved in multiple biological processes. A clear distinction was observed between the processes affected by downregulated (Supplementary Fig. S2A) and upregulated (Supplementary Fig. S2B) miRNAs in response to infection. The cellular response to stimulus, which includes response to infection, was represented in the upregulated miRNAs only (Supplementary Fig. S2B). Additionally, the conjunction of cellular metabolic processes (cellular metabolic process, regulation of metabolic process, nitrogen compound metabolic process, primary metabolic process, and organic substance metabolic process) represented 62% of the genes affected by upregulated miRNAs (Supplementary Fig. S2B) but was not represented in downregulated miRNAs (Supplementary Fig. S2A). These results showed differences in the genes putatively affected by miRNAs differentially regulated in response to *A. phagocytophilum* and provided additional support for the role of upregulated miRNAs in response to infection.

### The putative mRNA targets for isc-mir-79 were not affected by A. phagocytophilum infection in tick vector cells

**Figure 3:**
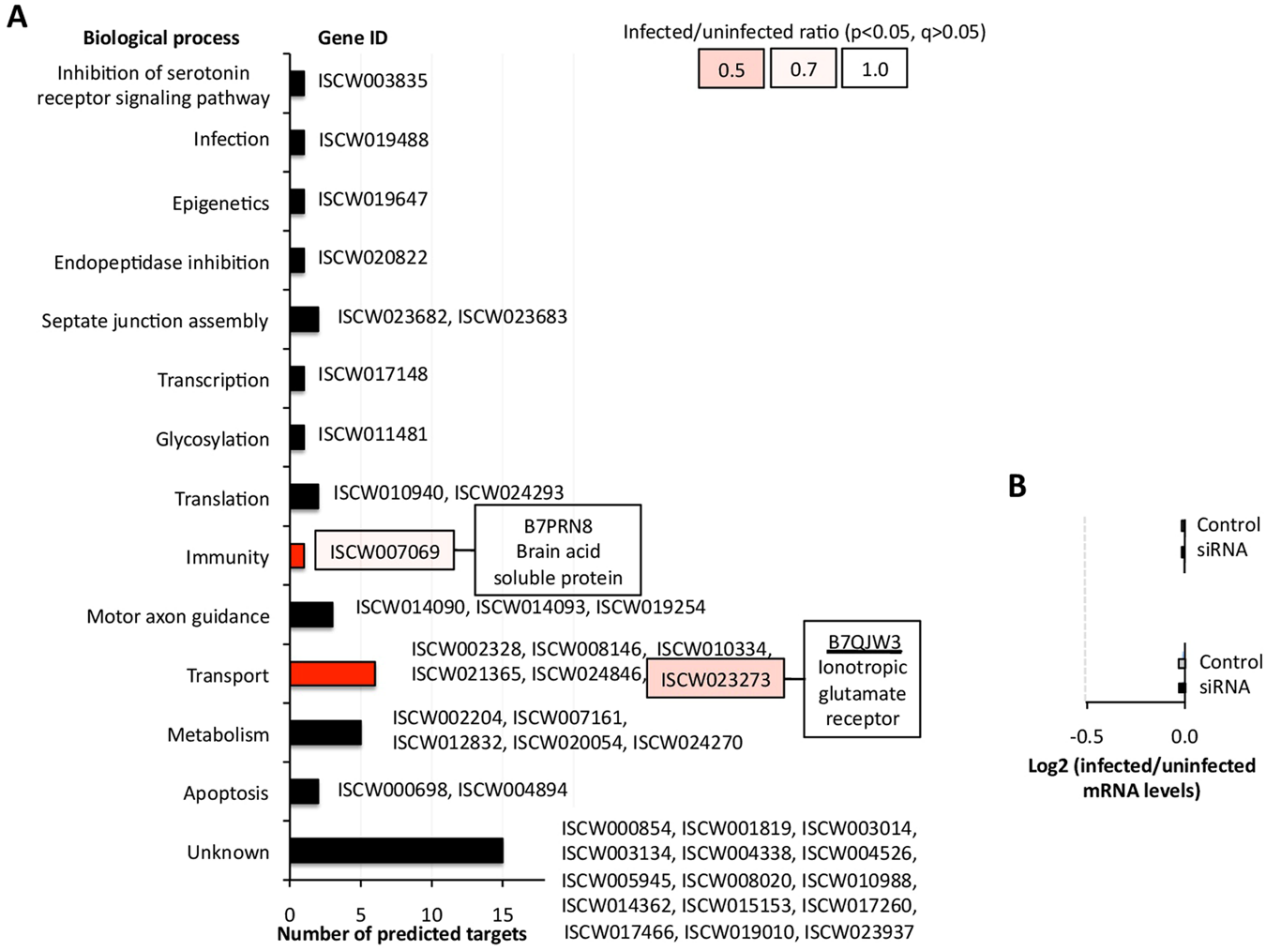
Putative targets for isc-mir-79 in the mRNA of genes downregulated in response to *A. phagocytophilum* infection of tick ISE6 cells. (**A**) Putative isc-mir-79 targets were predicted using RNAhybrid (https://bibiserv2.cebitec.uni-bielefeld.de/rnahybrid). Biological process functional annotations were done using Blast2GO. Data for genes significantly downregulated (p < 0.05) in response to *A. phagocytophilum* infection of tick ISE6 cells were obtained from Villar *et al*.^19^. Protein identity is shown, and the protein identified in the proteome of tick ISE6 cells (Villar *et al*.^19^) is underlined. (**B**) Regulation of predicted isc-mir-79 target mRNAs in response to isc-mir-79 knockdown and *A. phagocytophilum* infection. Only genes that were downregulated in response to *A. phagocytophilum* infection of tick ISE6 cells were included. The mRNA levels were normalized against tick *rps4*, and normalized Ct values were represented as the log2 (infected to uninfected ratio) and compared between control uninfected and control infected (Control) or isc-mir-79 siRNA-treated infected (siRNA) tick cells by Student’s t-test with unequal variance (p > 0.05; N = 3 biological replicates). None of the genes were confirmed as putative mRNA targets for isc-mir-79.

Based on the role of miRNAs in suppressing gene expression by binding to mRNA coding regions^18^, *in silico* analysis predicted 42 isc-mir-79 targets in *I. scapularis* mRNAs encoding proteins involved in different biological processes (Fig. 3A). Of them, only two corresponded to genes that were downregulated in response to *A. phagocytophylum* infection of tick ISE6 cells (p < 0.05)^19^, and were present in the immunity (*Brain acid soluble protein*, *BASP*) and transport (*Ionotropic glutamate receptor*, *iGluR*) biological processes (Fig. 3A). However, at the protein level only iGluR was previously identified in tick ISE6 cells^19^ with one protein encoded by the isc-mir-79 putative target gene ISCW023273 (B7QJW3), and a second protein (B7Q022) encoded by ISCW010196.

The expression of predicted isc-mir-79 mRNA targets in the genes downregulated in response to infection was then characterized in response to isc-mir-79 knockdown and *A. phagocytophilum* infection (Fig. 3B). The results confirmed the transcriptomics data by showing that mRNA levels for these genes did not change in response to infection, and were not affected by isc-mir-79 knockdown (Fig. 3B). Therefore, none of the isc-mir-79 putative mRNA targets were confirmed based on the absence of genes significantly downregulated in response to infection but not after miRNA knockdown (Fig. 3B).

### The gene encoding for Roundabout protein 2 (Robo2) is a putative 3′-UTR target for isc-mir-79 in tick vector cells

**Figure 4:**
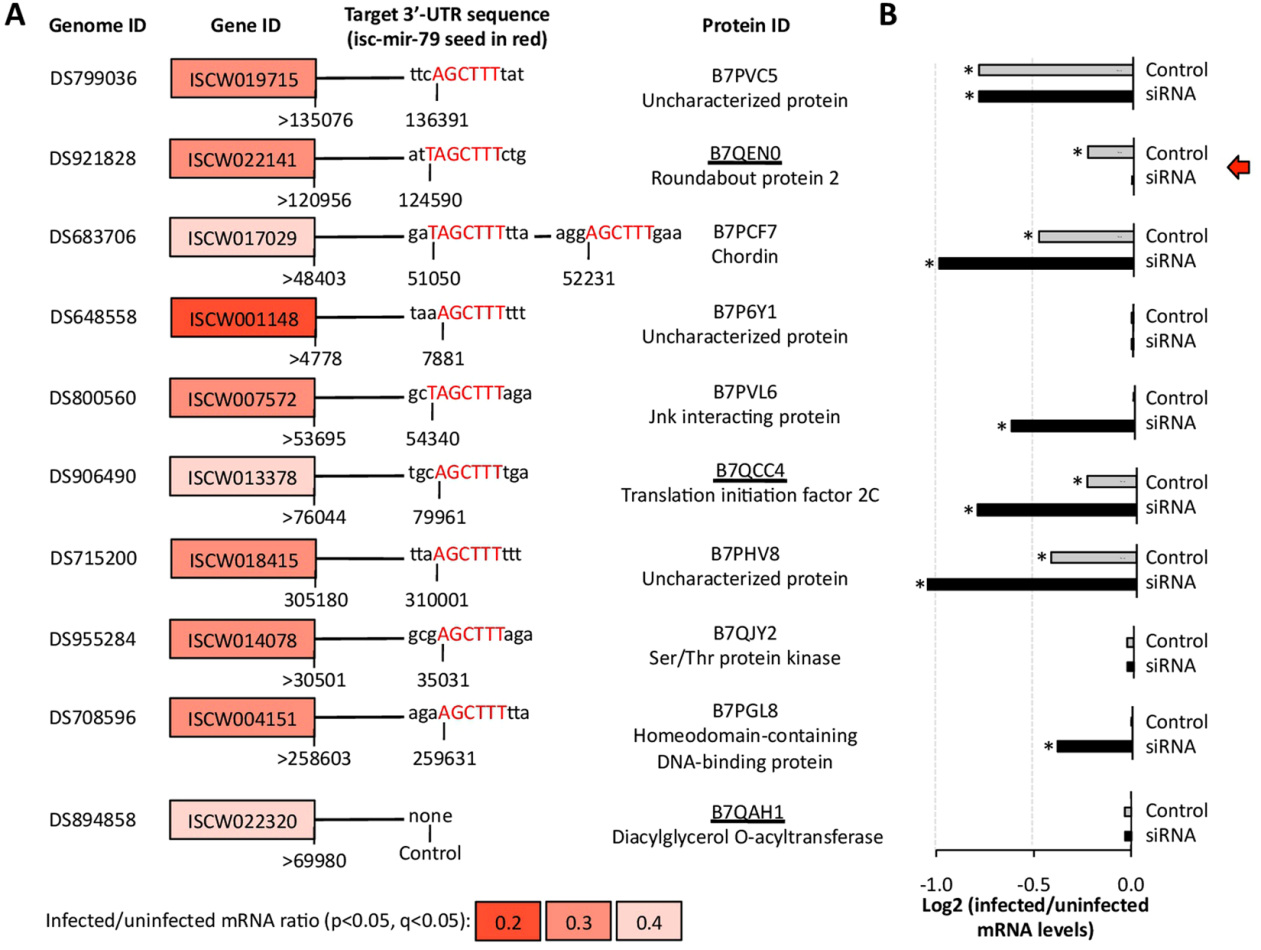
Putative targets for isc-mir-79 in the 3′-UTR of genes downregulated in response to *A. phagocytophilum* infection of tick ISE6 cells. (**A**) Data for genes significantly downregulated (p < 0.05) in response to *A. phagocytophilum* infection of tick ISE6 cells were obtained from Villar et al.^19^. Putative target sites (6–8 bp; seed sequence 5′-AGCTTTA-3′) for mature isc-mir-79 (3′-AUUGAAACCAUUGGAUCGAAAAUA-5′) were predicted using *I. scapularis* genome sequence of corresponding 3′-UTRs (60–5000 bp) as AGCTTT, AGCTTTA, TAGCTTT, TAGCTTTA (weaker to stronger prediction). Proteins identified in the proteome of tick ISE6 cells (Villar *et al*.^19^) are underlined. (**B**) Regulation of predicted isc-mir-79 target genes in response to isc-mir-79 knockdown and *A. phagocytophilum* infection. The mRNA levels were normalized against tick *rps4*, and normalized Ct values were represented as the log2 (infected to uninfected ratio) and compared between control uninfected and control infected (Control) or isc-mir-79 siRNA-treated infected (siRNA) tick cells by Student’s t-test with unequal variance (*p < 0.05; N = 3 biological replicates). The identified putative target for isc-mir-79 (red arrow) corresponds to the gene significantly downregulated in response to infection but not after miRNA knockdown.

Based on the role of miRNAs in suppressing gene expression by binding to UTRs^17,18^, targets for isc-mir-79 were predicted in the 3′-UTRs of the genes previously shown to be significantly downregulated in response to *A. phagocytophilum* infection of tick ISE6 cells (p < 0.05)^19^ (Fig. 4A). The ISCW022320 gene that was downregulated in response to infection but without predicted targets for isc-mir-79 was included in the analysis as control (Fig. 4A). Three of the proteins encoded by these genes were previously identified in the proteome of tick ISE6 cells (Fig. 4A). The regulation of predicted isc-mir-79 targets was then characterized in response to isc-mir-79 knockdown and *A. phagocytophilum* infection (Fig. 4B). The results confirmed downregulation in response to infection in 5/10 (50%) of the genes (Fig. 4B). Furthermore, the results identified *Robo2* as a putative target for isc-mir-79 based on a gene significantly downregulated in response to infection but not after miRNA knockdown (Fig. 4B).

**Figure 5:**
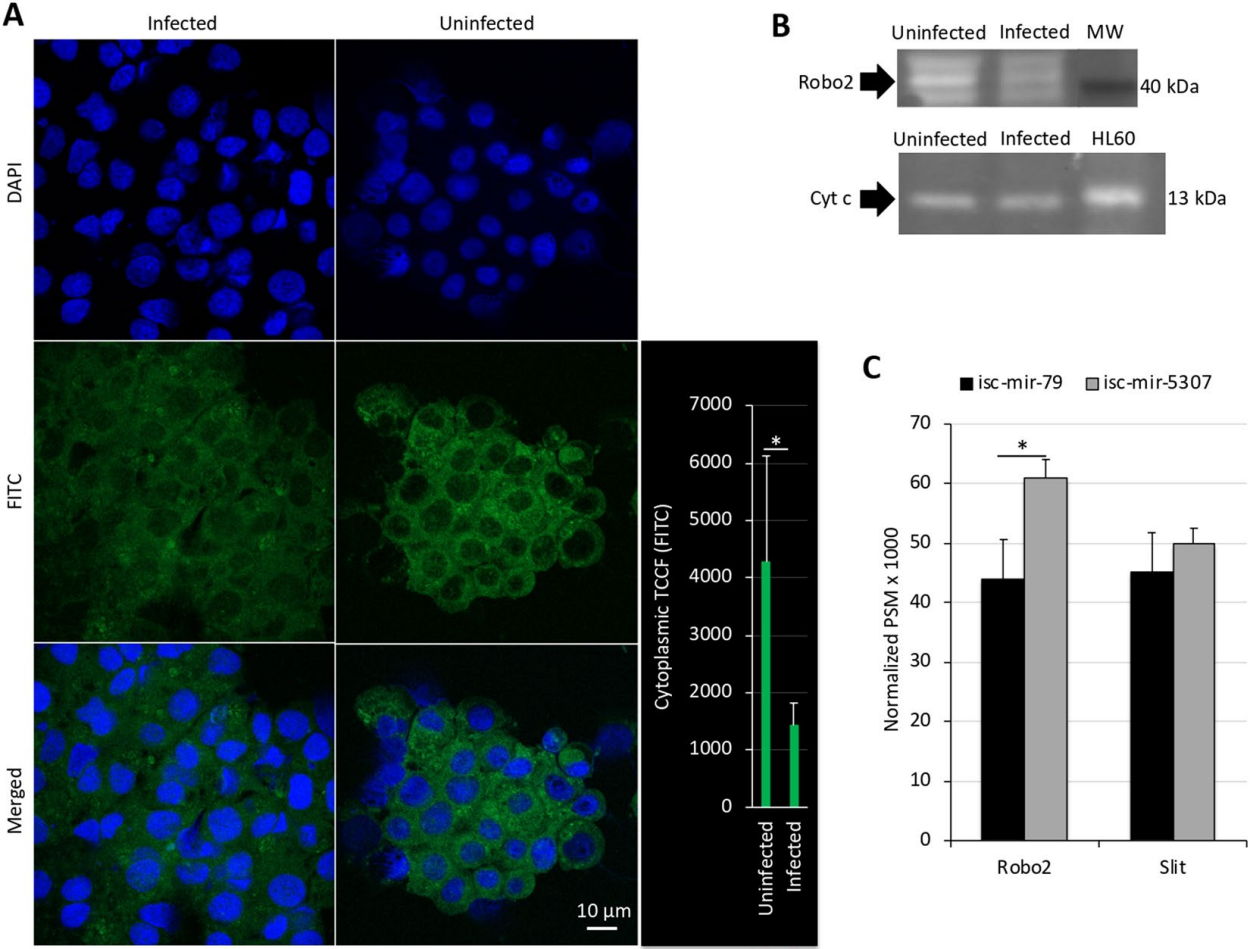
Protein levels of putative isc-mir-79 targets in tick ISE6 cells. (**A**) Representative images of immunofluorescence analysis of uninfected and *A. phagocytophilum*-infected ISE6 tick cells incubated with mouse anti-Robo antibodies. Goat anti-mouse IgG-FITC secondary antibodies were used to label Robo (green). Host cell nucleus was stained with DAPI (blue). Bar, 10 µm. The total cytoplasmic corrected cellular fluorescence (TCCF) was calculated as integrated density − (area of selected cell × mean fluorescence of background readings) and compared between infected and uninfected cells by Student’s t-test with unequal variance (*p = 0.001; N = 10 biological replicates). (**B**) Western-blot analysis of tick Robo2 and Cyt c (control protein not affected by *A. phagocytophilum* infection) levels in uninfected and *A. phagocytophilum*-infected ISE6 tick cells incubated with anti-Robo or anti-Cyt c antibodies. The position for tick Robo2 (predicted molecular weight, 42 kDa) and Cyt c (predicted molecular weight, 13 kDa) is shown with an arrow. For Cyt c, proteins from human HL60 cells were included as a positive control. (**C**) The isc-mir-79 and control isc-mir-5307 miRNA levels were increased in tick ISE6 cells with dsRNA miRNA mimics. Proteomics analysis was targeted at Robo2 (B7QEN0) and Slit (B7PL46) proteins identified as an isc-mir-79 3′-UTR target or not affected by this miRNA, respectively. The results of the normalized PSM x 1000 values were compared between groups by Chi2-test (*p < 0.05; N = 3 biological replicates).

Four putative Robo paralog sequences were identified in the *I. scapularis* genome (Supplementary Fig. S3A). The profile in response to *A. phagocytophilum* infection of *Robo* and *Slit*, the genes encoding for Robo receptor and Slit ligand proteins was compiled from previously published transcriptomics and proteomics analyses in *I. scapularis* ISE6 cells^19^ (Supplementary Fig. S3B). The expression of three *Robo* paralogs was detected in tick ISE6 cells with downregulation in response to infection (Supplementary Fig. S3B). At the protein level, only the putative isc-mir-79 target Robo2 was identified and its levels also decreased in infected cells when compared to uninfected controls (Supplementary Fig. S3B), a result that was corroborated by immunofluorescence (Fig. 5A) and Western blot (Fig. 5B and Supplementary Fig. S4) in ISE6 cells, and in the midguts of infected *I. scapularis* (Supplementary Fig. S5A,B). For Slit, the mRNA and protein levels did not change or increase, respectively in response to infection (Supplementary Fig. S3B).

### The gene encoding for Robo2 is a target for isc-mir-79 in tick vector cells

The results of the analyses at the mRNA level supported that *Robo2* is a putative isc-mir-79 target in tick ISE6 cells (Figs. 4A,B), thus fulfilling criteria for the selection of miRNA targets (Supplementary Fig. S1).

To address the effect of isc-mir-79 at the protein level for the final identification of isc-mir-79 targets (Supplementary Fig. S1), a proteomics analysis was conducted in tick ISE6 cells treated with synthetic isc-mir-79 and control isc-mir-5307 (Fig. 5C). Proteomics analysis was targeted at Robo2 (B7QEN0) and Slit (B7PL46), proteins in the Robo pathway identified as an isc-mir-79 3′-UTR target or not affected by this miRNA, respectively (Figs. 4A,B). The results of combined analyses at the mRNA and protein levels confirmed that Robo2 is an isc-mir-79 target in tick ISE6 cells (Fig. 5C).

### The isc-mir-79 has a role in A. phagocytophilum infection of tick vector cells

**Figure 6:**
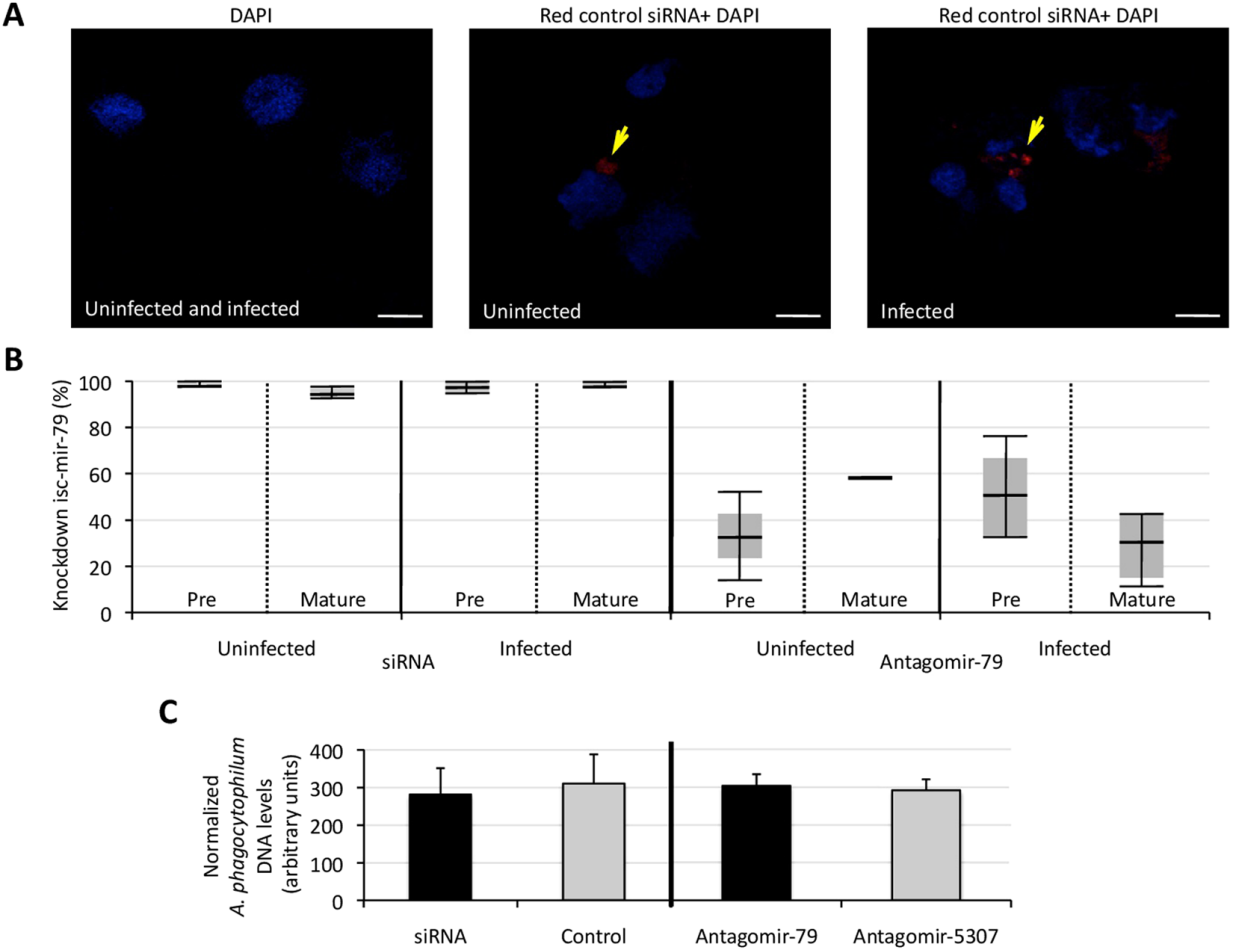
Knockdown of isc-mir-79 and effect on *A. phagocytophilum* infection of tick ISE6 cells. (**A**) Representative images to confirm the delivery of siRNA into tick ISE6 cells. Cytocentrifuge preparations of ISE6 cells treated with an Accell red fluorescent non-targeting control siRNA (red, yellow arrows) were mounted using Prolong Gold antifade reagent with DAPI (blue). Bars, 10 µm. (**B**) The isc-mir-79 was knockdown using a isc-mir-79 miRNA-specific custom siRNA or isc-mir-79 and isc-mir-5307 miRNA-specific antisense oligonucleotide antagomirs. The miRNA levels were characterized in uninfected and *A. phagocytophilum*-infected tick cells and normalized against tick *rps4* to calculate the knockdown percent with respect to medium-treated or isc-mir-5307 antagomir-treated cells for siRNA and Antagomir-79, respectively. (**C**) After treatment with siRNA or antagomirs, tick ISE6 cells were infected with *A. phagocytophilum*. DNA samples were analyzed by real-time qPCR using the *A. phagocytophilum msp4* and normalized against tick *16 S rRNA*. Normalized Ct values were compared between treated and control cells by Student’s t-test with unequal variance (p > 0.05; N = 3 biological replicates).

**Figure 7:**
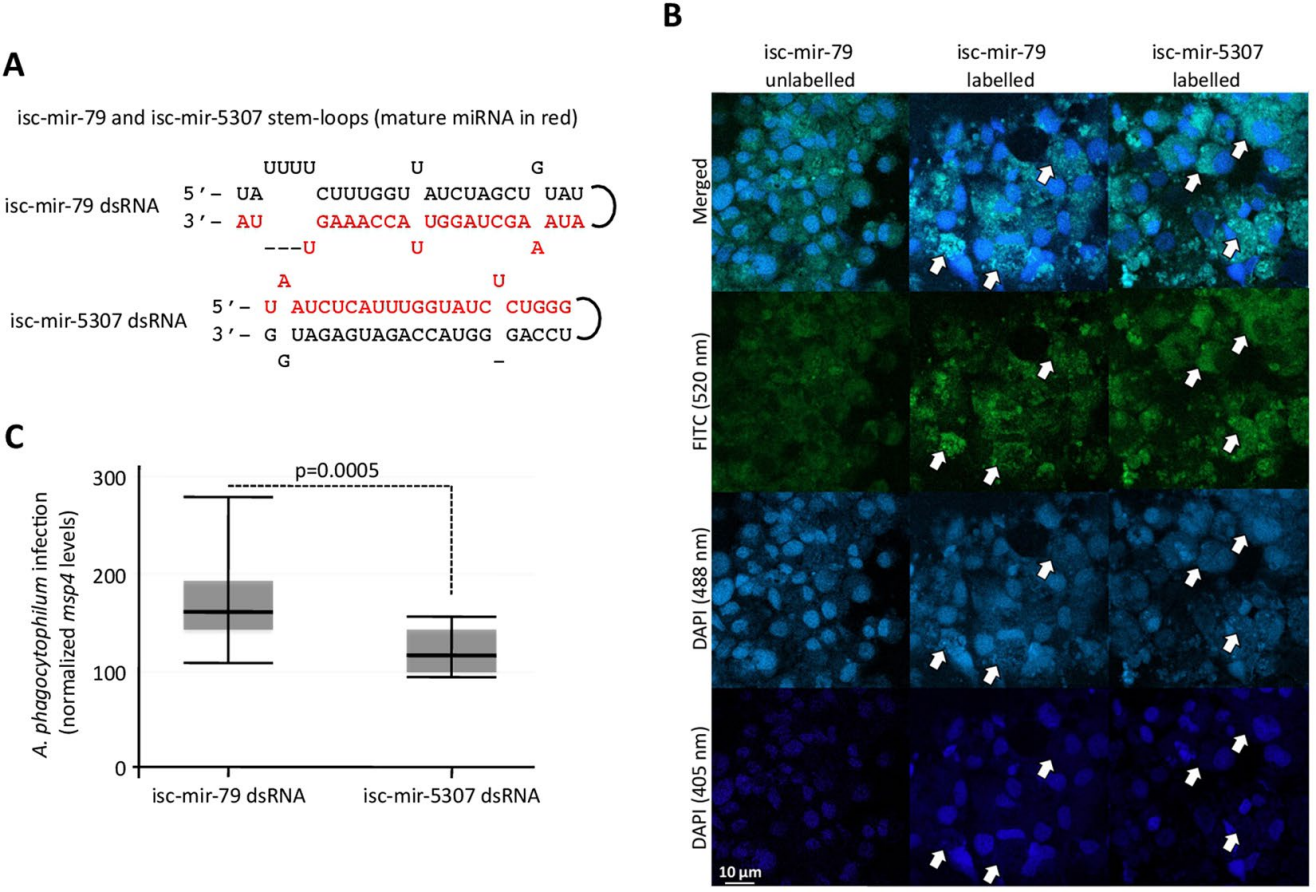
Increase of isc-mir-79 levels and effect on *A. phagocytophilum* infection of tick ISE6 cells. (**A**) The miRNA dsRNA mimics were constructed using RNA oligonucleotides for isc-mir-79 and isc-mir-5307 and their complementary sequences. (**B**) The delivery of miRNA mimics into tick ISE6 cells was confirmed using fluorescent labeled and unlabeled miRNA mimics. Emission peak wavelengths are for DAPI RNA (500 nm), DAPI DNA (460 nm) and FITC (519 nm). Therefore, fluorescence was detected at 405 nm (closer to DNA then RNA), 488 nm (closer to RNA than DNA) and 520 nm for FITC. White arrows show examples of regions with miRNA labeling and some RNA labeling but without DNA labeling, thus corresponding to miRNA mimics inside the cell. (**C**) Tick ISE6 cells were incubated with dsRNA, washed and then treated with fresh medium containing cell free *A. phagocytophilum*. DNA samples were analyzed by real-time qPCR using the *A. phagocytophilum msp4* and normalized against tick *16 S rRNA*. Normalized Ct values were compared between isc-mir-79 and isc-mir-5307 dsRNA treated cells by Student’s t-test with unequal variance (p = 0.0005; N = 4 biological replicates).

To gain insights into the role of isc-mir-79 during *A. phagocytophilum* infection of tick ISE6 cells, its levels were knockdown (Fig. 6A–C) or increased (Fig. 7A–C) before infection. Two complimentary methods based on isc-mir-79 miRNA-specific custom siRNA or isc-mir-79 and isc-mir-5307 miRNA-specific antisense oligonucleotide antagomirs were used for miRNA knockdown (Fig. 6A–C). Delivery of the isc-mir-79 siRNA to tick cells was confirmed (Fig. 6A), and provided a close to 100% (97–100%) knockdown, which was higher than using the antagomirs (12–76%) (Fig. 6B). Nevertheless, *A. phagocytophilum* infection levels did not change in response to isc-mir-79 knockdowm (Fig. 6C).

The miRNA dsRNA mimics were constructed and used to increase the isc-mir-79 and isc-mir-5307 levels in tick cells (Fig. 7A). The delivery of dsRNAs to tick cells was confirmed (Fig. 7B), and the results showed higher *A. phagocytophilum* infection in response to increase in isc-mir-79 when compared to isc-mir-5307 levels (Fig. 7C).

These results showed that although *A. phagocytophilum* infection was not affected after isc-mir-79 knockdown (Fig. 6C), an increase in isc-mir-79 levels resulted in higher bacterial levels in tick cells (Fig. 7C), suggesting a role for this miRNA during pathogen infection of tick vector cells.

### Anaplasma phagocytophilum upregulates isc-mir-79 to target Robo and facilitate infection

**Figure 8:**
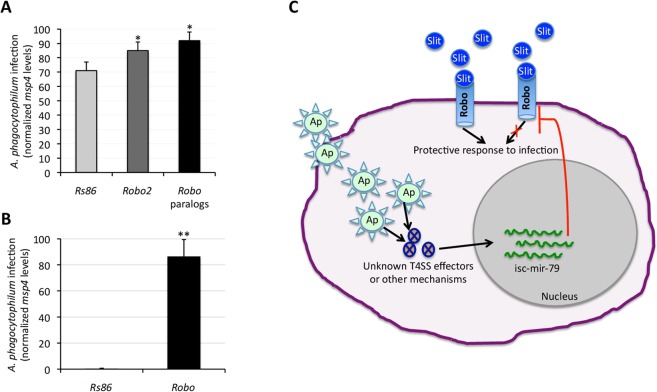
Proposed mechanism affected by isc-mir-79 to facilitate pathogen infection. (**A,B**) Tick ISE6 cells were incubated with siRNAs targeting *Robo2* or *Robo* paralogs, washed and then treated with fresh medium containing cell free *A. phagocytophilum* ((**A**) high infection: 90–100% infected cells; B, low infection: 5–10% infected cells). Control cells were treated with siRNAs targeting the unrelated *Rs86* gene. DNA samples were analyzed by real-time qPCR using the *A. phagocytophilum msp4* and normalized against tick *16 S rRNA*. Normalized Ct values were compared between *Robo* and *Rs86* siRNAs treated cells by Chi^2^ test (*p < 0.0005; **p < 0.00005; N = 4 biological replicates). (**C**) Based on these results we speculated that *A. phagocytophilum* uses unknown type IV secretion system (T4SS) effectors or other mechanisms to promote isc-mir-79 overexpression and target *Robo2* to suppress or diminish protective proinflammatory responses mediated by the Slit-Robo pathway to facilitate infection.

To characterize the biological function of isc-mir-79 during *A. phagocytophilum* infection of tick vector cells (Supplementary Fig. S1), experiments were conducted to determine the effect of *Robo* knockdown on pathogen infection. For *Robo* knockdown by RNAi, siRNAs were synthesized to target both *Robo2*-specific and *Robo* paralogs-common regions (Supplementary Fig. S3A). The results showed that knockdown of *Robo2*, *Robo* paralogs and *Robo2* + *Robo* paralogs (*Robo*) resulted in 1.2-fold, 1.3-fold and 144-fold higher *A. phagocytophilum* infection levels (p < 0.0005), respectively when compared to *Rs86* control (Fig. 8A,B). These results showed that Robo is involved in the tick protective response to *A. phagocytophilum* infection, and suggested a mechanism by which pathogen infection induces isc-mir-79 overexpression targeting *Robo2* to suppress or diminish protective proinflammatory responses mediated by the Slit-Robo pathway to facilitate infection (Fig. 8C). Additionally, it appeared that Robo2 has a role in maintaining tick cell health (Supplementary Fig. S6A–C). After *Robo2* knockdown, the number of live cells decreased over time accompanied by an increase in apoptotic cells when compared to *Rs86* control (Supplementary Fig. S6A–C).

## Discussion

Host miRNAs have been implicated in the regulation of proteins involved in innate and adaptive immune response to pathogen infection, but pathogens could also modify host miRNA profile to facilitate infection and multiplication^12–16^. Pathogen induction or suppression of host miRNAs could result in the inhibition of the host immune response to facilitate infection and multiplication^13^. In particular, intracellular bacteria such as *Mycobacterium* spp., *Listeria monocytogenes*, *Francisella tularensis*, *Salmonella enterica*, and tick-borne *Rickettsia rickettsii* manipulate host miRNA expression to inhibit immune response and apoptosis^13,14,22^. However, the molecular mechanisms by which these bacteria manipulate host cell miRNAs are currently unknown^13^.

Few studies have addressed the functional role of tick miRNAs^23–29^. The results of these studies have suggested that tick miRNAs are involved in processes such as feeding, development, blood digestion, sex differentiation and reproduction, innate immunity, and regulation of host homeostasis. However, the function of tick miRNAs during pathogen infection has not been characterized.

As previously reported in other tick species^23^, in this study we predicted thousands of new miRNAs based on sequence identity to other species (Supplementary Table S1), and showed that tick miRNA profile changes in response to *A. phagocytophilum* infection with a putative impact on multiple biological processes involved in tick-pathogen interactions^8^. Particularly relevant was the effect of miRNAs upregulated in response to infection and putatively targeting genes involved in cellular response to infection and metabolic processes. These processes have been previously characterized as being manipulated by *A. phagocytophilum* during infection of tick cells^8,30,31^. However, to provide insights into the functional role of tick miRNAs during pathogen infection we focused on the characterization of isc-mir-79, which was annotated in the response to infection biological process and upregulated in infected tick cells when compared to uninfected controls.

The isc-mir-79, which belongs to the mir-9 gene family MI0012285 has been implicated in the regulation of several biological processes such as cell differentiation, neurogenesis, apoptosis and immunity, and has been implicated in cancer and viral infectious diseases^32–36^. The sequence of the seed region of mir-9/mir-79 is identical in vertebrates and invertebrates, suggesting that these miRNAs are evolutionarily conserved and may share a common ancestor^32,34,37^. Functionally, mir-9/mir-79 seems to have the ancient feature of a context-dependent role in apoptosis^32^. In *Rhipicephalus microplus*, the rmi-mir-79 levels were shown to increase during tick life cycle from eggs to adult females with an effect of host exposure in larvae resulting in lower miRNA levels after feeding^23^. However, in *Ixodes ricinus* salivary glands the iri-mir-9a-5p levels were higher in nymphs than in adult female ticks while in nymphs the miRNA levels were higher in salivary glands than in midguts^28^. In *Rhipicephalus haemaphysaloides*, mir-79/mir-9 was shown to be downregulated in response to lipopolysaccharide (LPS) induction in female and male ticks^26^. Based on these results the authors suggested that downregulation of tick mir-79 may be involved in the LPS-mediated stimulation of the innate immune response. Recently, a new miRNA, HLWS-m0017 with sequence similar to mir-79 was identified in *Haemaphysalis longicornis* as putatively involved in different biological processes such as immune system process^29^.

However, the role of mir-9/mir-79 during bacterial infection has been only recently characterized in human cells infected with *Pseudomonas aeruginosa*^38^. They reported that the expression of a soluble pattern recognition receptor (*PTX3*) was induced to protect cells from bacterial infection through GroEL (heat shock protein 60)-Toll-like receptor 4 (TLR4) pathway via nuclear factor-kappa B (NF-κB) and the inhibition of mir-9, which targets *PTX3*. However, in tick cells the isc-mir-79 was induced and not inhibited in response to *A. phagocytophilum* infection, suggesting a different role for this miRNA in tick cells during bacterial infection.

Based on the role of mir-9/mir-79 in host immune response^26,29,36,38^, the induction of isc-mir-79 could be due to a tick cell response to limit *A. phagocytophilum* infection or a mechanism used by the pathogen to facilitate infection. The isc-mir-79 knockdown in tick ISE6 cells did not affect *A. phagocytophilum* infection, which could reflect the absence of a role for this miRNA during pathogen infection or the fact that miRNA downregulation by itself is not sufficient to affect infection. Supporting the second option, the increase in isc-mir-79 levels resulted in higher *A. phagocytophilum* infection when compared to controls, suggesting that the pathogen may benefit from isc-mir-79 upregulation to facilitate infection.

To gain information on the possible mechanisms affected by isc-mir-79 to facilitate pathogen infection, *Robo2* was identified as a putative miRNA target and its knockdown increased *A. phagocytophilum* infection. Robo2 belongs to the Robo immunoglobulin superfamily of proteins that are highly conserved from planarians to humans, and constitute transmembrane receptors for Slit molecules that function in axon guidance, cell mobility and permeability, and virus infection^39–43^. In particular, it has been shown in human endothelial cells that Robo1 has proinflammatory properties and Slit2 downregulates its expression via mir-218 encoded in a *Slit2* intron to suppress inflammatory responses^42^.

Recently, Shaw *et al*.^44^ showed that infection-derived lipids induce the proinflammatory immune deficiency (IMD) pathway in ticks, which protects ticks against infection by pathogens such as *A. phagocytophilum*. Other tick innate immune response pathways such as the Toll and Janus Kinase/Signal Transducers and Activators of Transcription (JAK/STAT) are also activated in response to infection^3,8,45,46^. Therefore, it may be possible that *A. phagocytophilum* benefits from isc-mir-79 overexpression to target *Robo2* to suppress or diminish protective proinflammatory responses similar to those mediated by the IMD, Toll and JAK/STAT pathways to facilitate infection (Fig. 8C).

The mechanism used by *A. phagocytophilum* to modify host miRNA profile to facilitate infection and multiplication is not known, but considering that the isc-mir-79 is encoded in a non-protein coding region, we speculated that it may include epigenetic mechanisms^47^ that have been described before to play a role in tick-*A. phagocytophilum* interactions^48^. If supported by additional experiments, these results discovered a new mechanism involved in immunity to pathogen infection in ticks mediated by Slit-Robo signaling pathways, and how *A. phagocytophilum* diminishes its activation through isc-mir-79 upregulation to facilitate infection. Furthermore, these results suggested new targets for interventions to control pathogen infection in ticks^49^.

## Methods

### Experimental design

The study was designed to characterize the miRNA profile and function in tick vector cells in response to infection with the intracellular bacterium *A. phagocytophilum* (Supplementary Fig. S1). The experimental pipeline was based on the approach previously proposed by Kuhn *et al*.^21^. The *I. scapularis* embryo-derived ISE6 cells, which constitute a model for hemocytes^19,20^ were used to characterize the effect of *A. phagocytophilum* infection on tick miRNAs. The tick miRNAs were sequenced in infected and uninfected cells, and their differential expression was determined in response to infection. The miRNA-seq results were validated for selected miRNAs by qRT-PCR, and the putative function and targets of tick miRNAs differentially expressed in response to infection was proposed. The study was then focused on the upregulated isc-mir-79 involved in response to infection. The function of this miRNA during *A. phagocytophilum* infection was then established by characterizing the target genes and their function in tick cells.

### I. scapularis tick ISE6 cells and miRNA isolation

The *I. scapularis* embryo-derived tick ISE6 cells were cultured in L-15B300 medium as described previously^20,50^, except that the osmotic pressure was lowered by the addition of one-fourth sterile water by volume. Tick ISE6 cells (25 cm^2^ flasks with approximately 10^7^ cells per flask) were experimentally infected with the human isolate of *A. phagocytophilum* NY18 as described previously^51,52^. Uninfected tick cells were cultured in parallel under similar conditions for comparison. Two replicates were included for each infected and uninfected cells. At 12 days post-infection (dpi), cells (percent infected cells 68–73% (Ave ± SD, 70 ± 3) were collected. The percentage of cells infected with *A. phagocytophilum* was calculated by examining at least 200 cells using a 100x oil immersion objective. The miRNAs were extracted using a Purelink miRNA isolation kit (Invitrogen, Thermo Scientific, Waltham, MA, USA) following manufacturers recommendations. The miRNA samples from uninfected (U2, U4) and infected (NY3 and NY5) cells were stored at −80 °C until sequencing.

### Sequencing of miRNA in I. scapularis ISE6 tick cell and data processing

The infected (NY3 and NY5) and uninfected (U2, U4) tick ISE6 samples were used for miRNA-seq by abm (Richmond BC, Canada). Quality check of all samples was carried out using the Agilent 2100 Bioanalyzer with the Small RNA kit (Agilent Technologies, Santa Clara, CA, US) to determine sample quality and miRNA concentration. The four samples passed abm internal quality control with good bioanalyzer profiles and enough miRNA in each sample to proceed with library construction (Supplementary file 1). The libraries were prepared using the TruSeq Small RNA Sample Prep Kit (Illumina, San Diego, CA, USA), where the miRNA samples were subjected to ligation of the 3′ and 5′ adapters, and reverse transcription. Unique barcodes were incorporated during the PCR enrichment step to ensure sufficient amount of library is generated. Adapter dimers were removed and the small RNA libraries were purified by polyacrilamide gel electrophoresis (PAGE). Resulting libraries were run on the Agilent Bioanalyzer with the DNA High Sensitivity Chip (Agilent Technologies) to determine library concentration and size distribution profile. Real-time quantification (KAPA qPCR quantification for Illumina systems, KAPA Bio Systems, Roche Holding AG, Basel, Switzerland) was performed to quantify the libraries prior to sequencing with Illumina NextSeq. 500, which uses two channel chemistry to reduce the sequencing time while maintaining unparallel accuracies of the data. Samples with different barcodes were pooled together prior to sequencing but were separated post sequencing through demutiplexing. Resulting miRNA-seq single end reads and binary base call (Bcl) files were converted to Fastq data immediately after the run and used to generate Cufflinks output (Supplementary file 1).

For data processing, reads were mapped using Bowtie integrated into Tophat 2.1.0 (https://ccb.jhu.edu/software/tophat/index.shtml). The reads were mapped to the reference *I. scapularis* genome (assembly JCVI_ISG_i3_1.0; NZ_ABJB000000000.1) as if they were from a non-small RNA-seq experiment to obtain reads mapping to coding (protein and putative miRNA coding sequences) and non-coding regions. Transcripts assembly and abundance estimation was performed using Cufflinks 2.2.1 (http://cole-trapnell-lab.github.io/cufflinks/) by reads assembly with Cufflinks, transcripts merge and comparison with the reference genome with Cuffmerge, and analysis of differential expression with Cuffdiff according to the number of fragments mapping to each gene^53^. Selected transcripts for miRNAs were searched against the miRBase (http://www.mirbase.org) to identify *I. scapularis* known miRNAs and putative new miRNAs (classified as intergenic and ≤ 125 bp) (Supplementary Table S1). Data were deposited at NCBI under study name “Effect of *Anaplasma phagocytophilum* infection on the micro RNA profile of *Ixodes scapularis* tick cells” and GEO accession number GSE79324 (http://www.ncbi.nlm.nih.gov/geo/query/acc.cgi?acc=GSE79324).

### Functional annotation of differentially expressed tick miRNAs

Differentially expressed miRNAs (p < 0.05, q < 0.05) were functionally annotated based on the previously published information for homologous miRNAs in ticks and other species (Supplementary Table S1). The prediction of gene targets for differentially expressed miRNAs was done using RNAhybrid (https://bibiserv2.cebitec.uni-bielefeld.de/rnahybrid)^54^ with the parameters (a) energy threshold ∆G < −25 kcal/mol, f-value ≤ 0.001) previously reported by Shao *et al*.^25^, (b) no G:U mismatch was allowed in the seed sequence, (c) miRNA seeds were forced to form a helix with respect to the query sequence, and (d) no bulge or internal loops were allowed. Functional annotations were done using Blast2GO as previously reported^19,55^ using the VectorBase (https://www.vectorbase.org) and Uniprot (http://www.uniprot.org) databases. The quantitative transcriptomics data for uninfected and *A. phagocytophilum*-infected *I. scapularis* ISE6 cells were obtained from previously published results^19^. Genes were annotated based on the p-value threshold as significantly (p < 0.05) downregulated in response to *A. phagocytophilum* infection. For tick isc-mir-79 (MI0012285; gene family MIPF0000014, mir-9), putative targets in the 3′-UTR of genes differentially downregulated in response to *A. phagocytophilum* infection of tick ISE6 cells^19^ were predicted by searching the corresponding *I. scapularis* genome scaffold for target sites (6–8 bp; seed sequence 5′-AGCTTT) in the 3′-UTRs (60–5000 bp) as AGCTTT, AGCTTTA, TAGCTTT, TAGCTTTA (weaker to stronger prediction).

### Characterization of miRNA levels

The isc-mir-79, isc-mir-185, isc-mir-7544, isc-mir-9771b, isc-mir-2951, isc-mir-B8, and isc-mir-491 that were upregulated in response to infection within the response to infection functional category were selected for analysis by qRT-PCR. The isc-mir-5307, which levels did not change in response to infection (infected/uninfected ratio = 1.3; p = 0.65), was included as control. Total RNA was extracted from uninfected and *A. phagocytophilum*-infected ISE6 cells using Tri Reagent (Sigma-Aldrich, St. Louis, MO, USA) following manufacturer’s recommendations and used to characterize the expression of precursor (pre) and/or mature isc-mir-79, isc-mir-185, isc-mir-7544, isc-mir-9771b, isc-mir-2951, isc-mir-B8, isc-mir-491, and isc-mir-5307 by qRT-PCR. The cDNA was prepared with the miRNA reverse transcription miScript II RT kit (Qiagen, Hilden, Germany). Forwards primers (pre-isc-mir-79: 5′-AGAGGATGAAGGCGCTG-3′, mature-isc-mir-79: 5′-ATAAAGCTAGGTTACCAAAGTTA-3′, mature-isc-mir-185: 5′-GGAGAGAAAGGCAGTTGCTGG-3′, mature-isc-mir-7544: 5′-CATCTGAAAAATGTTGTTAT-3′, mature-isc-mir-9771b: 5′-AAAGTCAGGCCATAAACCAG-3′, mature-isc-mir-2951: 5′-AGTCGAAGTTACGGAACCGGG-3′, mature-isc-mir-B8: 5′-CAGCCTTGTCGGAACTAGGC-3′, mature-isc-mir-491: 5′-CTGCCGGGTTCCCCACGAA-3′, mature-isc-mir-5307: 5′-TAATCTCATTTGGTATCTCTGGG-3′) were specifically designed for each miRNA and used in combination with miScript universal reverse primer for qPCR using the Kapa SYBR Fast One-Step qRT-PCR Kit (Sigma-Aldrich) in a Rotor-Gene Q real-time PCR cycler (Qiagen) following manufacturer’s recommendations. The miRNA levels were normalized against tick *ribosomal protein S4* (*rps4*) levels using the genNorm method (ddCT)^3^. Normalized Ct values were compared between infected and uninfected tick cells by Student’s t-test with unequal variance (p = 0.05; N = 3 biological replicates).

### miRNA knockdown

Two different methods were used to knockdown isc-mir-79 expression. In the first method, a miRNA-specific custom Accell siRNA (GE Healthcare Dharmacon Inc. Lafayette, CO, USA) was used. Tick ISE6 cells were incubated with 10 μl of 10 µM siRNA and 90 µl of fresh medium in 24-well plates using three replicates per treatment. Controls were incubated with medium alone. After 48 h of siRNA exposure, medium was removed and replaced with fresh medium containing cell free *A. phagocytophilum*. One ml of infected medium or medium alone was added per well for infected and uninfected cells, respectively. Cells were incubated for 48 h and then collected for RNA and DNA extraction to characterize miRNA and *A. phagocytophilum* DNA levels, respectively. To confirm the delivery of siRNA into tick ISE6 cells, cytocentrifuge preparations of ISE6 cells treated with an Accell red fluorescent non-targeting control siRNA were mounted using Prolong Gold antifade reagent with DAPI (Molecular Probes, Eugene, OR, USA). Slides were examined using a Zeiss LSM 800 laser scanning confocal microscope (Carl Zeiss, Oberkochen, Germany).

The second method used antagomirs, miRNA-specific antisense oligonucleotides (GE Healthcare Dharmacon Inc.). Antagomirs were specific for isc-mir-79 and isc-mir-5307 as control, and chemically modified as described previously^56^. Silencing experiments were conducted by incubating tick ISE6 cells with 100 nM antagomirs in 500 µl per well in 24-well plates using three replicates per treatment. After 48 h of antagomir exposure, medium containing the antagomirs was removed and replaced with fresh medium containing cell free *A. phagocytophilum*. One ml of infected medium or medium alone was added per well for infected and uninfected cells, respectively. As when using siRNA, cells were incubated for 48 h and then collected for RNA and DNA extraction to characterize miRNA and *A. phagocytophilum* DNA levels, respectively.

### Increase in miRNA levels

The miRNA mimics were constructed using RNA oligonucleotides for isc-mir-79 and isc-mir-5307 and their complementary sequences (Sigma-Aldrich). Each pair of RNA oligonucleotides was incubated for 5 min at 75 °C and then cooled down to room temperature (RT) to form dsRNA. To increase the miRNA levels, tick ISE6 cells were incubated in 24-well plates with 100 nM of dsRNA in 500 µl per well using four replicates per treatment. After 48 h of dsRNA exposure, medium containing the dsRNA was removed and replaced with 500 µl/well fresh medium containing cell free *A. phagocytophilum*. Cells were incubated for 48 h and then collected for DNA extraction to characterize *A. phagocytophilum* infection levels.

### miRNA mimics fluorescent labeling

To confirm the delivery of miRNA mimics into tick ISE6 cells, miRNA mimics for isc-mir-79 and isc-mir-5307 were labeled with Alexa Fluor 488 using the Ulysis Alexa Fluor 488 Nucleic Acid Labeling Kit (Molecular Probes, Eugene, OR, USA), then purified using Micro Bio-Spin P-30 Gel Columns with SSC Buffer (Bio-Rad, Hercules, CA, USA) according to manufacturer’s protocols, and labeled strands hybridized with their complementary strand to form dsRNA. Tick ISE6 tick cells were incubated with 2 µM of miRNA mimics in 500 µl of fresh medium in a 24-well plate using two wells per treatment. After 48 h of miRNA mimic exposure, cells were washed with PBS and collected for fluorescence microscopy studies. Non-labeled miRNA mimics were used as negative controls. Tick cells were fixed and permeabilized with the Intracell fixation and permeabilization kit (Immunostep, Salamanca, Spain) following manufacturer recommendations. The slides were washed twice with PBS and mounted in ProLong Antifade with DAPI reagent (Molecular Probes) for examination using a Zeiss LSM 800 laser scanning confocal microscope (Carl Zeiss, Oberkochen, Germany).

### Characterization of A. phagocytophilum DNA levels

Total DNA was extracted from tick ISE6 cells using a NucleoSpin Tissue kit (Macherey-Nagel, Fisher Scientific, Madrid, Spain). DNA samples were analyzed by real-time qPCR using the *A. phagocytophilum major surface protein 4* (*msp4*) gene-specific primers and normalized against tick *16S rRNA* using the genNorm method (ddCT) as previously described^57^. Normalized Ct values were compared between treated and control cells by Student’s t-test with unequal variance (p = 0.05; N = 3–4 biological replicates).

### Characterization of putative isc-mir-79 target mRNA levels

Tick ISE6 cells were treated with the isc-mir-79-specific siRNA or medium alone as control and infected or not with *A. phagocytophilum* as described above. Total RNA was extracted using Tri Reagent (Sigma-Aldrich) following manufacturer’s recommendations. To determine putative target gene mRNA levels by qRT-PCR, the KAPA SYBR FAST One-Step qRT-PCR Kit (KAPA Bio Systems) was used following manufacturer’s recommendations together with gene-specific oligonucleotide primers and PCR conditions (Table 1). The mRNA levels were normalized against tick *rps4* levels using the genNorm method (ddCT)^3^. Normalized Ct values were compared between control uninfected and control infected or isc-mir-79 siRNA-treated infected tick cells by Student’s t-test with unequal variance (p = 0.05; N = 3 biological replicates).

**Table 1:** Sequence of oligonucleotide primers and annealing temperature for the qRT-PCR analysis of putative isc-mir-79 targets.

Gene ID	Forward and reverse primer sequences (5′-3′)	Annealing temperature
ISCW019715	B7PVC5_F: TGCTGCAAATTCTCCGGCTA B7PVC5_R: TCGAACTTGTGGCAGGTGTT	55 °C
ISCW022141	B7QEN0_F: AGCTCTTCTTCCTGCACGTC B7QEN0_R: GGTAATATGCGGGACTGCGA	57 °C
ISCW017029	B7PCF7_F: CACCTTTTGGCTTCAGTCGC B7PCF7_R: GCGACTTGACTGGGATGACA	57 °C
ISCW001148	B7P6Y1_F: CTGGTGTACCTGGTCGTCCC B7P6Y1_R: GTACCGGAGTTGACGGGTG	57 °C
ISCW022320	B7QAH1_ F: GATCTCCATCTGGGAGTGCG B7QAH1_ R: AGCAGAACCTCGTGAAGAGC	57 °C
ISCW007572	B7PVL6_F: GCTCACGACAAGCACAAAGG B7PVL6_R: CAGATTCGGAAGCACAGGGT	57 °C
ISCW013378	B7QCC4_F: CGGGCCAATCACTTCCAGAT B7QCC4_R: ATCTCCCTGTTGACCTTGCG	57 °C
ISCW018415	B7PHV8_F: GCTTCTGTGTACCTCGGCTT B7PHV8_R: TCTCCGCATAGTTGTCGCTC	57 °C
ISCW014078	B7QJY2_F: CACGGATCTGGTTCAGGGAC B7QJY2_R: TGACCTTTCACGTCTCGGTG	57 °C
ISCW004151	B7PGL8_F: CTTCCCAGCGAGTTGACCAT B7PGL8_R: TCATCAGGTACTGCATCGGC	57 °C
ISCW023273	GlutamateR_F: CTGCAAGAGGCAGGGAAACT GlutamateR_R: TCCTCCTTGGCCATCTTTCG	57 °C
ISCW007069	BrainP_F: CCGGCCAGGTGAACTATGAG BrainP_R: CCTGCCCTTGGAGCTTCTG	57 °C

### Proteomics characterization of predicted isc-mir-79 target Robo2 protein levels

To increase the isc-mir-79 and control isc-mir-5307 miRNA levels, tick ISE6 cells were incubated with 100 nM of dsRNA miRNA mimics as described above using three replicates per treatment. Proteins were extracted using the AllPrep DNA/RNA/Protein Mini Kit (Qiagen) according to manufacturer instructions. Precipitated proteins were resuspended in 10 mM PBS with 2% SDS and protein concentration was determined using the BCA Protein Assay (Thermo Scientific) using bovine serum albumin (BSA) as standard.

Protein extracts (80 µg per sample) were methanol/chloroform precipitated and proteins were digested using the filter aided sample preparation (FASP) protocol^58^. Samples were dissolved in 30 μl 50 mM Tris-HCl pH 7.5 supplemented with 2% SDS and 50 mM DTT. Proteins were diluted in 8 M urea in 0.1 M Tris-HCl pH 7.5 (UA), loaded onto 30 kDa centrifugal filter devices (FASP Protein Digestion Kit, Expedeon, TN, USA) and washed three times with UA. Proteins were later alkylated using 50 mM iodoacetamide in UA for 20 min in the dark, and the excess of alkylation reagents were eliminated by washing three times with UA and three additional times with 50 mM ammonium bicarbonate. Proteins were digested overnight at 37 °C with sequencing grade trypsin (Promega, Madison, WI, USA) in 50 mM ammonium bicarbonate at 40:1 protein:trypsin (w/w) ratio. The resulting peptides were eluted by centrifugation with 50 mM ammonium bicarbonate (twice) and 0.5 M sodium chloride. Trifluoroacetic acid (TFA) was added to a final concentration of 1% and the peptides were finally desalted onto OMIX Pipette tips C18 (Agilent Technologies), dried-down and stored at −20 °C until mass spectrometry analysis. The desalted protein digests were resuspended in 0.1% formic acid and analyzed by reverse phase liquid chromatography-mass spectrometry (RP-LC-MS/MS) using an Easy-nLC II system coupled to an ion trap LTQ mass spectrometer (Thermo Scientific). The peptides were concentrated (on-line) by reverse phase chromatography using a 0.1 × 20 mm C18 RP precolumn (Thermo Scientific), and then separated using a 0.075 × 100 mm C18 RP column (Thermo Scientific) operating at 0.3 ml/min. Peptides were eluted using a 180-min gradient from 5% to 40% solvent B in solvent A (Solvent A: 0.1% formic acid in water, solvent B: 0.1% formic acid in acetonitrile). ESI ionization was done using a Fused-silica Pico-Tip Emitter ID 10 mm (New Objective, Woburn, MA, USA) interface. Peptides were detected in survey scans from 400 to 1600 amu (1 mscan), followed by twelve data dependent MS/MS scans, using an isolation width of 2 mass-to-charge ratio units, normalized collision energy of 35%, and dynamic exclusion applied during 30 sec periods. The MS/MS raw files were searched against the Uniprot - *Ixodes scapularis* proteome database (20,473 entries in January 2019) (http://www.uniprot.org) using the SEQUEST algorithm (Proteome Discoverer 1.4, Thermo Scientific). The following constraints were used for the searches: tryptic cleavage after Arg and Lys, up to two missed cleavage sites, and tolerances of 1 Da for precursor ions and 0.8 Da for MS/MS fragment ions and the searches were performed allowing optional Met oxidation and Cys carbamidomethylation. A false discovery rate (FDR) <0.05 was considered as condition for successful peptide assignments and at least two peptides per protein were the necessary condition for protein identification. For the quantitative analysis of Robo2 and Slit proteins, the total number of peptide-spectrum matches (PSMs) for each protein was normalized against the total number of PSMs in each sample and compared between isc-mir-79 and isc-mir-5307-treated control cells by Chi2-test (p < 0.05).

### Robo gene knockdown by RNAi

The identified putative target for isc-mir-79, the genes encoding for Robo proteins, were selected for knockdown by RNAi in tick ISE6 cells. For RNAi, siRNAs targeting Robo2 (*Robo2* siRNA1: 5′ CAG ACG UUG CGC AGA GAU A 3′, *Robo2* siRNA2: 5′ GAG AGU GGC CGC UGC CUA A 3′) or Robo paralogs (*Robo* siRNA1: 5′ CUC UAC UGG UGU ACG GCA A 3′, *Robo* siRNA2: 5′ CGG AGG GAC AGC UCU UCU U 3′) were synthesized (Cultek S.L., Madrid, Spain). The unrelated gene *Rs86* (*Rs86* siRNA1: 5′ CGG UAA AUG UCG AAG CAA A 3′, *Rs86* siRNA2: 5′ GCG AAU AUG AAG UCG GUA A 3′) was used as negative control^56^. RNAi experiments were conducted in cell cultures by incubating tick ISE6 cells with 10 μl of 10 µM siRNA and 90 µl of fresh L15B medium in 24-well plates using 4 wells per treatment. Control cells were incubated with the unrelated *Rs86* siRNAs. After 48 h of siRNA exposure, tick cells were infected with cell-free *A. phagocytophilum* obtained from approximately 5 × 10^6^ infected HL-60 cells (high infection: 90–100% infected cells; low infection: 5–10% infected cells) and resuspended in 24 ml culture medium to use 1 ml/well or mock infected by adding the same volume of culture medium alone. Cells were incubated for an additional 48 h, harvested and used for DNA and RNA extraction. RNA was used to analyze gene knockdown by real-time qRT-PCR with respect to *Rs86* control as previously described^57^. DNA was used to quantify the *A. phagocytophilum* infection levels by *msp4* qPCR as described above.

### Immunofluorescence assay (IFA) in tick ISE6 cells

Uninfected and *A. phagocytophilum*-infected *I. scapularis* ISE6 tick cell slides were prepared using a cytocentrifuge. The slides were air-dried, fixed with ice-cold methanol for 10 min, then blocked with 3% BSA/PBS for 1 hr at RT. The slides were then incubated for 14 h at 4 °C with mouse anti-*Drosophila* Robo monoclonal antibodies (Creative Diagnostics, Shirley, NY, USA) diluted 1:64 in 3% BSA/PBS and, after 3 washes in PBS, developed for 1 h with goat anti-mouse IgG conjugated with FITC (Sigma-Aldrich) diluted 1:100 in 3% BSA/PBS. The slides were washed twice with PBS and mounted in ProLong Antifade with DAPI reagent (Molecular Probes, Eugene, OR, USA). The sections were examined using a Zeiss LSM 800 laser scanning confocal microscope (Carl Zeiss, Oberkochen, Germany) with a 63x oil immersion objective. Using ImageJ, an outline was drawn around each cell and area, mean fluorescence and integrated density were measured, along with several adjacent background readings. Then, the total cytoplasmic corrected cellular fluorescence (TCCF) was calculated as integrated density − (area of selected cell × mean fluorescence of background readings)^59^ and compared between infected and uninfected cells by Student’s t-test with unequal variance (p = 0.05; N = 10 biological replicates).

### Western blot analysis of Robo2 in tick ISE6 cells

Twenty-five μg of protein lysate from uninfected and *A. phagocytophilum*-infected *I. scapularis* ISE6 tick cells were methanol/chloroform precipitated, resuspended in Laemmli sample buffer and separated on a 12% sodium dodecyl sulfate (SDS) polyacrylamide precast gel (ClearPage Expedeon, VWR, Radnor, PA, USA). After electrophoresis, proteins were transferred to a nitrocellulose blotting membrane (GE Healthcare Dharmacon Inc.), blocked with 3% BSA (Sigma) in Tris-buffered saline (TBS; 50 mM Tris-Cl, pH 7.5, 150 mM NaCl) and incubated overnight at 4 °C with mouse anti-*Drosophila* Robo monoclonal antibodies (Creative Diagnostics) or rabbit anti-Cytochrome c (Cyt c) antibodies (H-104: sc-7159; Santa Cruz Biotechnology, Inc. Dallas, TX, USA)^3^ diluted 1:100 in 3% BSA/PBS. Cyt c was included as a control because protein levels are not affected by *A. phagocytophilum* infection of ticks and ISE6 cells^3,19^. For Cyt c, proteins from human HL60 cells were included as a positive control^3^. To detect the IgG antibodies bound to tick proteins, membranes were incubated with goat anti-mouse or anti-rabbit IgG peroxidase antibody (Sigma) diluted 1:1000 in 3% BSA/PBS. Immunoreactive proteins were visualized with chemiluminescence with Pierce ECL Western Blotting Substrate (Thermo Scientific).

## Supplementary information

Supplemental information

Table S2

Table S1
