# Supplementary material for: Anaplasma phagocytophilum modifies tick cell microRNA expression and upregulates isc-mir-79 to facilitate infection by targeting the Roundabout protein 2 pathway
Source: Sci Rep. 2019 Jun 24;9:9073. doi: 10.1038/s41598-019-45658-2 (PMC6591238; doi:10.1038/s41598-019-45658-2)
Supplement: Supplementary file 1 — Supplemental information [file 41598_2019_45658_MOESM1_ESM.pdf]

Anaplasma phagocytophilum modifies tick cell microRNA expression and upregulates isc-mir-79 to facilitate infection by targeting the Roundabout protein 2 pathway

Sara Artigas-Jerónimo, Pilar Alberdi, Margarita Villar Rayo, Alejandro Cabezas-Cruz, Pedro J. Espinosa Prados, Lourdes Mateos-Hernández, José de la Fuente

Supplemental figures and methods and Supplemental file 1

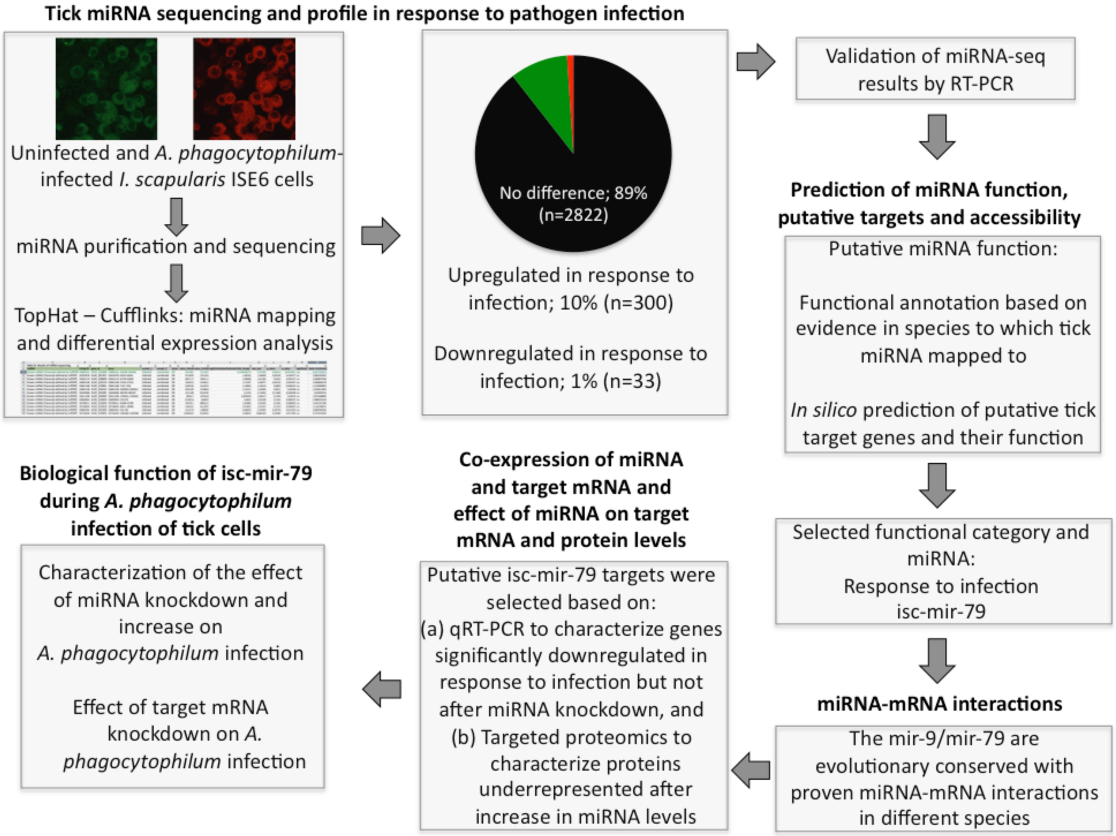

Figure S1. Experimental design. The study was designed based on the approach proposed by Kuhn et al. (2008) to characterize the miRNA profile and function in tick ISE6 cells in response to *A. phagocytophilum* infection. The differential expression of tick miRNAs in response to infection was characterized by miRNA-seq in infected and uninfected cells. The miRNA-seq results were validated for selected miRNAs by qRT-PCR, and the putative function and targets of tick miRNAs differentially expressed in response to infection was proposed. The study was then focused on the upregulated isc-mir-79 involved in response to infection. The function of this miRNA during *A. phagocytophilum* infection was then established by characterizing the target genes and their function in tick cells.

Reference

Kuhn DE, Martin MM, Feldman DS, Terry AV Jr, Nuovo GJ, Elton TS. 2008. Experimental Validation of miRNA Targets. *Methods* **44:** 47-54.

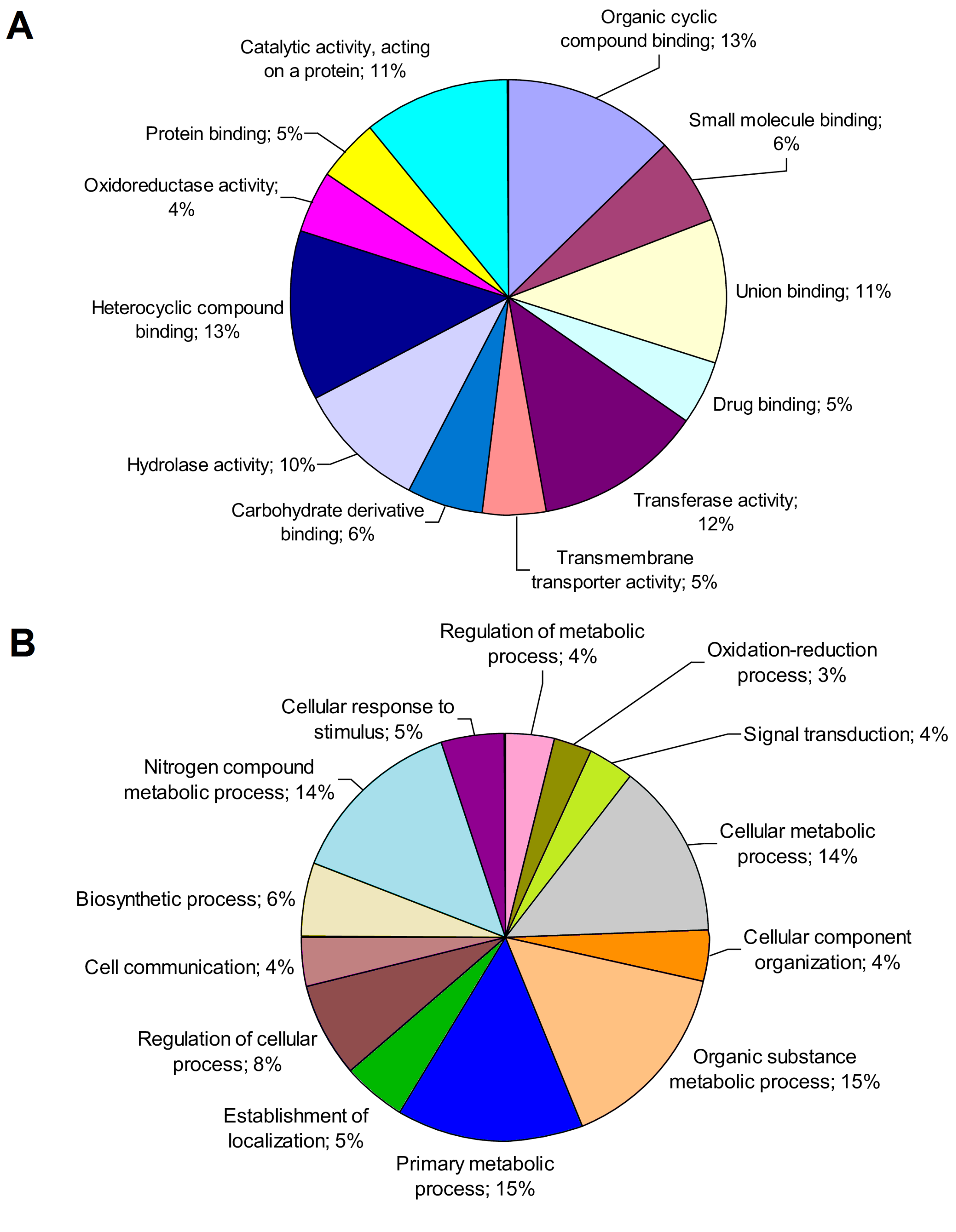

Figure S2. Predicted gene targets for differentially expressed miRNAs in response to *A. phagocytophilum* infection of tick ISE6 cells. Putative miRNA targets were predicted using RNAhybrid (https://bibiserv2.cebitec.uni-bielefeld.de/rnahybrid) for miRNA (A) downregulated and (B) upregulated in response to *A. phagocytophilum* infection. Biological process functional annotations were done using Blast2GO. A detailed list of gene targets is provided in Table S2.

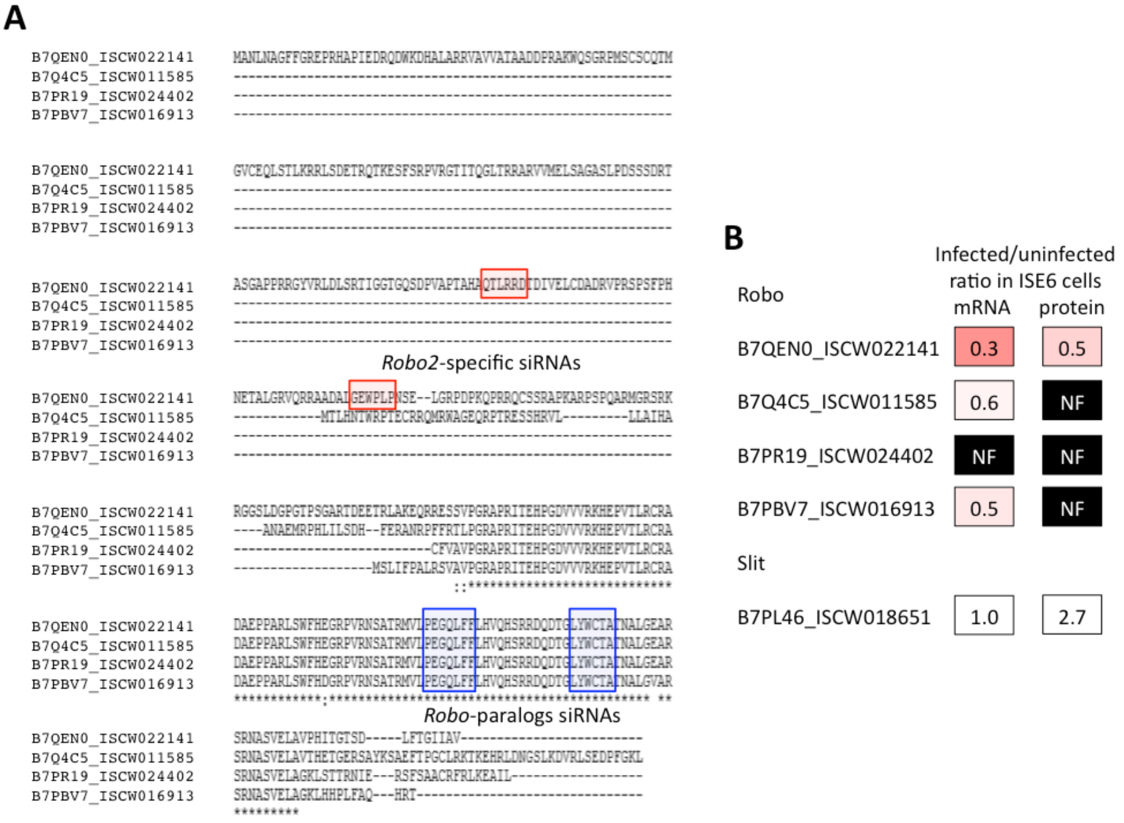

Figure S3. *I. scapularis* Robo and effect of *A. phagocytophilum* infection in tick ISE6 cells. (A) Four *Robo* paralogs were identified in *I. scapularis*, and dsRNAs wre synthesized to knockdown *Robo2* and all *Robo* paralogs expression by RNAi. (B) The gene and protein profiles of Robo and Slit in response to *A. phagocytophilum* infection is shown as infected to uninfected ratio and was compiled from previously published transcriptomics and proteomics analyses in *I. scapularis* ISE6 cells (Villar et al., 2015). Color code, decrease (red), no change or increase (white) or not found (NF; black) in infected cells when compared to uninfected controls.

Reference

Villar M, Ayllón N, Alberdi P, Moreno A, Moreno M, Tobes R, Mateos-Hernández L, Weisheit S, Bell-Sakyi L, de la Fuente J. 2015. Integrated metabolomics, transcriptomics and proteomics identifies metabolic pathways affected by *Anaplasma phagocytophilum* infection in tick cells. *Mol Cell Proteomics* **14:** 3154-3172.

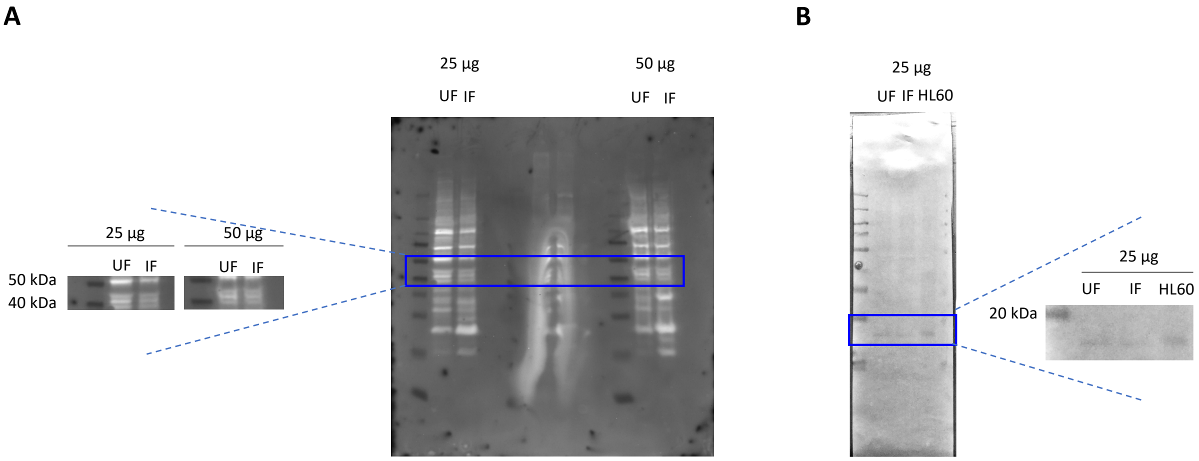

Figure S4. Western blot analysis of tick Robo2 levels in uninfected and *A. phagocytophilum*-infected ISE6 tick cells incubated with mouse anti-Robo antibodies. (A) Western blot in uninfected (UF) and infected (IF) ISE6 cells using anti-*Drosophila* Robo antibodies. The limitation of this analysis is that the *Drosophila* Robo1 protein used for Mab production is 46-48% identical (E-value = 0.0) to *I. scapularis* conserved hypothetical proteins XP_002407743.1, XP_002408970.1, XP_002401303.1 and XP_002411516.1, which in turn showed a 52% identity to Robo2 proteins A0A146MDD1_LYGHE and A0A146L1T0_LYGHE (Blastp). These results suggested a possible cross-reaction between the antibodies and tick Robo2. However, other proteins of unknown function and putative Robo paralogs may be also detected thus explaining the multiple bands detected in the Western blot. (B) Western blot in UF and IF ISE6 cells using anti-Cytochrome c (Cyt c) antibodies. The Cyt c was used because the levels of this protein do not change in response to *A. phagocytophilum* infection (Ayllón et al., 2015). Proteins from human HL60 cells were included as a positive control.

Reference

Ayllón N, Villar M, Galindo, RC, Kocan KM, Šíma R, López JA, Vázquez J, Alberdi P, Cabezas-Cruz A, Kopáček P, de la Fuente J. 2015. Systems biology of tissue-specific response to *Anaplasma phagocytophilum* reveals differentiated apoptosis in the tick vector Ixodes scapularis. *PLoS Genet* **11:** e1005120.

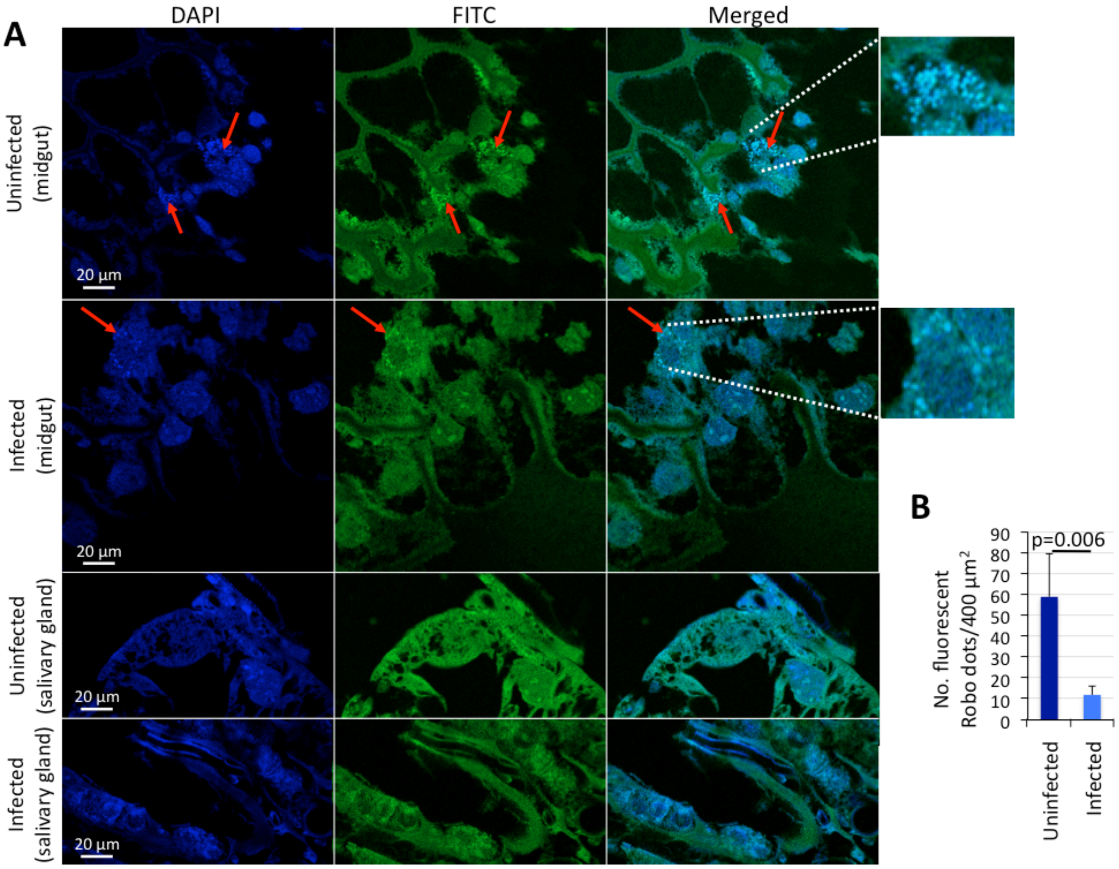

Figure S5. ***I. scapularis*** **Robo and effect of** ***A. phagocytophilum*** **infection in tick tissues.** (A) Robo protein levels were measured by immunofluorescence assays in tick tissues. Host cell nucleus was stained with DAPI (blue). Goat anti-mouse IgG-FITC antibody was used to detect Robo (green; arrows). Bar, 20 µm. (B) Robo signals were counted and represented as the number of fluorescent Robo dots per 400 μm^2 in uninfected and infected tick midguts, and compared by Student's t-test with unequal variance (p=0.05; N=4 biological replicates).

Methods

Adult *I. scapularis* females were infected with *A. phagocytophilum* (NY18) as described before (Kocan et al. 2012). Female ticks were removed from the sheep 10 days after infestation, held in the humidity chamber for 4 days and fixed with 4% paraformaldehyde in 0.2 M sodium cacodylate buffer, dehydrated in a graded series of ethanol and embedded in paraffin (Ayllón et al. 2015). Sections (4 µm) were prepared and mounted on glass slides. The paraffin was removed from the sections with xylene and the sections were hydrated by successive 2 min washes with a graded series of 100, 95, 80, 75, and 50% ethanol. The slides were treated with Proteinase K (Dako, Barcelona, Spain) for 7 min, washed with PBS and incubated with 3% BSA (Sigma-Aldrich) in PBS for 1 hr at RT. The slides were then incubated for 14 hrs at 4 ºC with mouse anti-*Drosophila* Robo monoclonal antibody (Creative Diagnostics, Shirley, NY, USA) diluted 1:64 in 3% BSA/PBS and, after 3 washes in PBS, developed for 1 hr with goat anti-mouse IgG conjugated with FITC (Sigma-Aldrich) diluted 1:100 in 3% BSA/PBS. The slides were

washed twice with PBS and mounted in ProLong Antifade with DAPI reagent (Molecular Probes, Eugene, OR, USA). The sections were examined using a Zeiss LSM 800 laser scanning confocal microscope (Carl Zeiss, Oberkochen, Germany). Sections of uninfected ticks and IgG from pre-immune sera were used as controls.

References

Ayllón N, Villar M, Galindo, RC, Kocan KM, Šíma R, López JA, Vázquez J, Alberdi P, Cabezas-Cruz A, Kopáček P, de la Fuente J. 2015. Systems biology of tissue-specific response to *Anaplasma phagocytophilum* reveals differentiated apoptosis in the tick vector Ixodes scapularis. *PLoS Genet* **11:** e1005120.

Kocan KM, Busby AT, Allison RW, Breshears MA, Coburn L, Galindo RC, Ayllón N, Blouin EF, de la Fuente J. 2012. Sheep experimentally-infected with a human isolate of *Anaplasma phagocytophilum* serve as a host for infection of *Ixodes scapularis*. *Ticks Tick-Borne Dis* **3:** 147-153.

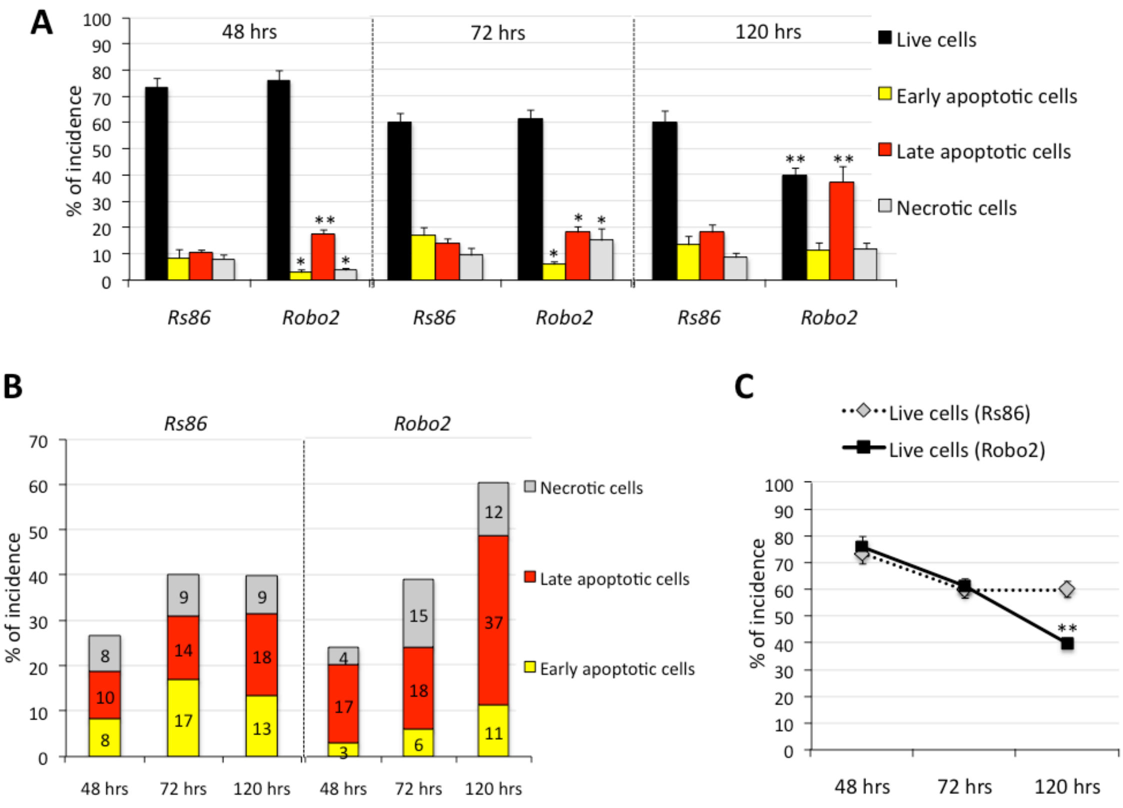

Figure S6. Effect of *Robo* knockdown on tick cell health. (A) Proportion of live, early apoptotic, late apoptotic and necrotic cells at 48, 72 and 120 hrs after *Robo* and *Rs86* control gene knockdown. (B) Variation in the percent of apoptotic and necrotic cells at 48, 72 and 120 hrs after *Robo* and *Rs86* control gene knockdown. (C) Variation in the percent of live cells at 48, 72 and 120 hrs after *Robo* and *Rs86* control gene knockdown.

Methods

Approximately $5x10^5$-$1x10^6$ uninfected tick cells were collected after *Rs86* or *Robo2* gene knockdown as described above. Cell health was measured by flow cytometry using the Annexin V-fluorescein isothiocyanate (FITC) apoptosis detection kit (Immunostep, Salamanca, Spain) following the manufacturers protocols. The technique detects changes in phospholipid symmetry analyzed by measuring Annexin V (labelled with FITC) binding to phosphatidylserine, which is exposed in the external surface of the cell membrane in apoptotic cells. Cells were stained simultaneously with the non-vital dye propidium iodide (PI) allowing the discrimination of live (Annexin V-FITC negative, PI positive), early apoptotic (Annexin V-FITC positive, PI negative), necrotic (Annexin V-FITC negative, PI positive), and late apoptotic (Annexin V-FITC positive, PI positive). All samples were analyzed on a FAC-Scalibur flow cytometer equipped with CellQuest Pro software (BD Bio-Sciences, Madrid, Spain). The viable cell population was gated according to forward-scatter and side-scatter parameters. The percentage of apoptotic cells was determined by flow cytometry after Annexin V-FITC and PIes labeling and compared between *Robo* and *Rs86* control gene knockdown cells by Student's t-test with unequal variance (*$p<0.05$, **$p<0.01$; N=3 biological replicates).

Supplemental file 1. Quality control of RNA samples, library construction and miRNA sequencing (FastQC report for each infected (NY3 and NY5) and uninfected (U2, U4) tick ISE6 samples.

Quality control of RNA samples

Sample	Small RNA	miRNA	% miRNA	RIN	Pass/Fail
ISE6 U2	32.0 ng/ul	3.6 ng/ul	11	N/A	Pass
ISE6 U4	26.3 ng/ul	3.1 ng/ul	12	N/A	Pass
ISE6 NY3	25.1 ng/ul	4.8 ng/ul	19	N/A	Pass
ISE6 NY5	22.3 ng/ul	2.7 ng/ul	12	N/A	Pass

Library construction

Sample	Library Size (Average)
ISE6 U2	155 bp
ISE6 U4	149 bp
ISE6 NY3	156 bp
ISE6 NY5	155 bp

Cluster generation and sequencing

Sample	Total Reads
ISE6 U2	42,027,955
ISE6 U4	11,232,949
ISE6 NY3	50,419,722
ISE6 NY5	35,765,024

Report

Summary

- Basic Statistics
- Per base sequence quality
- Per tile sequence quality
- Per sequence quality scores
- Per base sequence content
- Per sequence GC content
- Per base N content
- Sequence Length Distribution
- Sequence Duplication Levels
- Overrepresented sequences
- Adapter Content
- Kmer Content

Basic Statistics

Measure	Value
Filename	ISE6-NY3_R1.fastq
File type	Conventional base calls
Encoding	Sanger / Illumina 1.9
Total Sequences	50419722
Sequences flagged as poor quality	0
Sequence length	36
%GC	50

Per base sequence quality

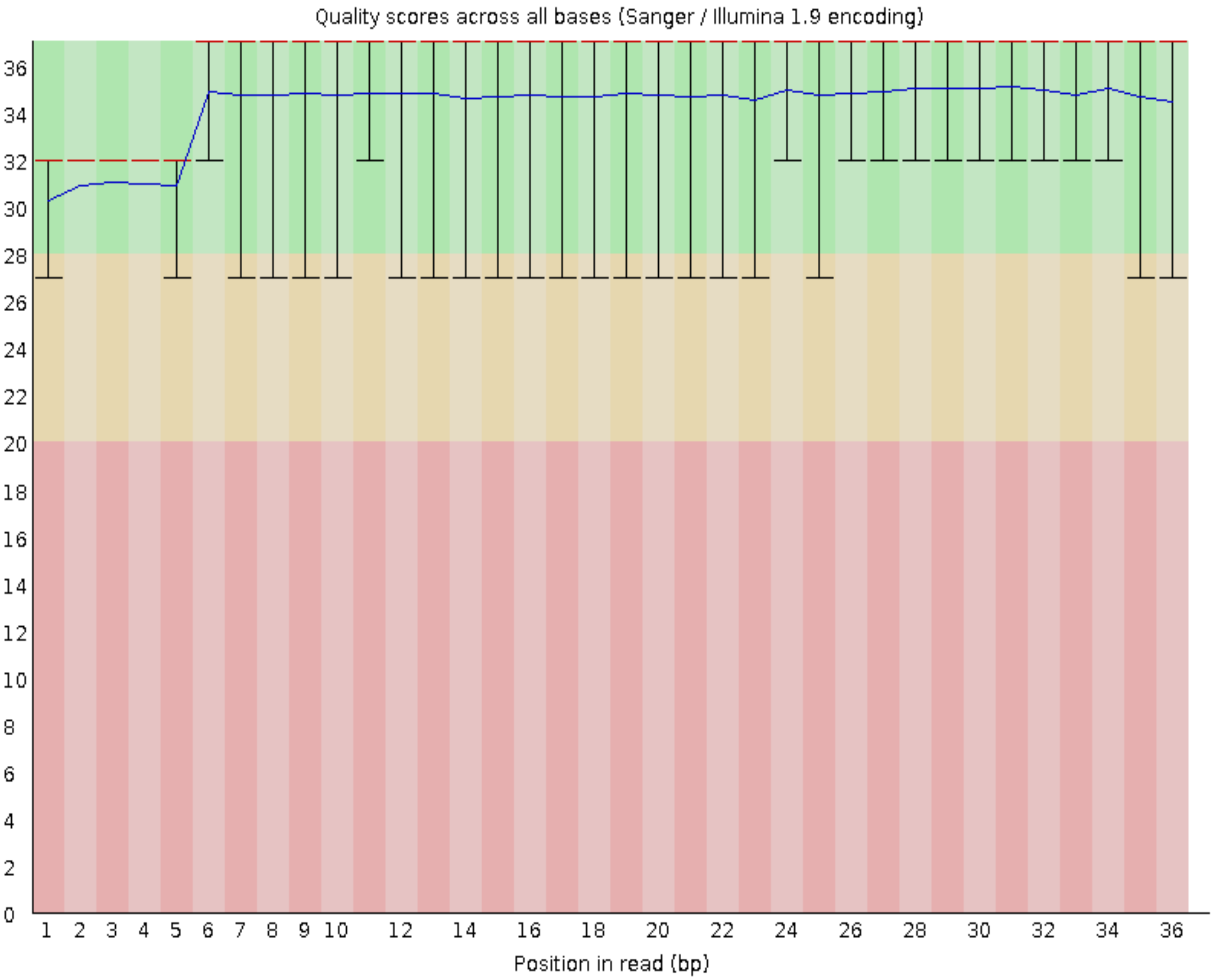

Per tile sequence quality

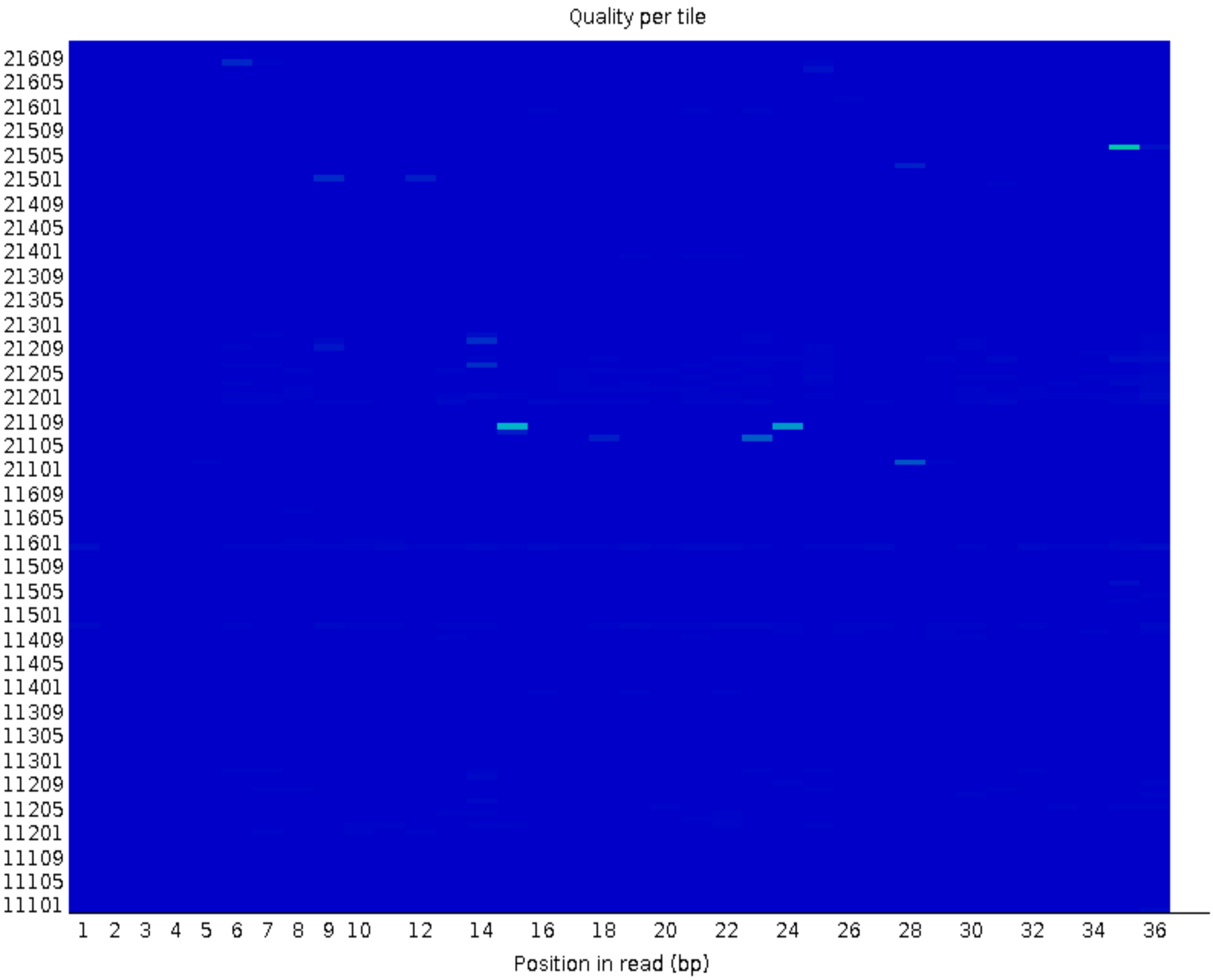

Per sequence quality scores

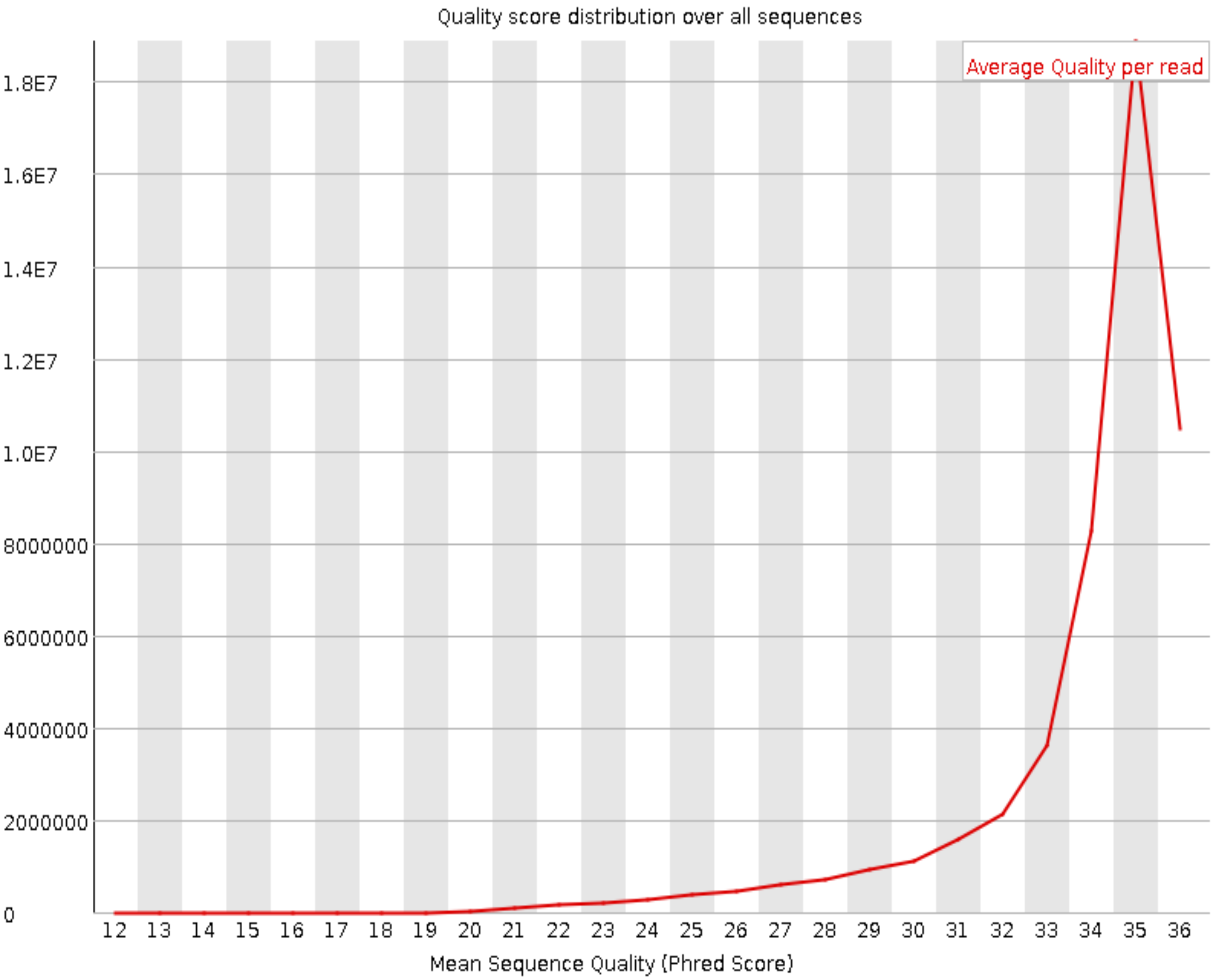

Per base sequence content

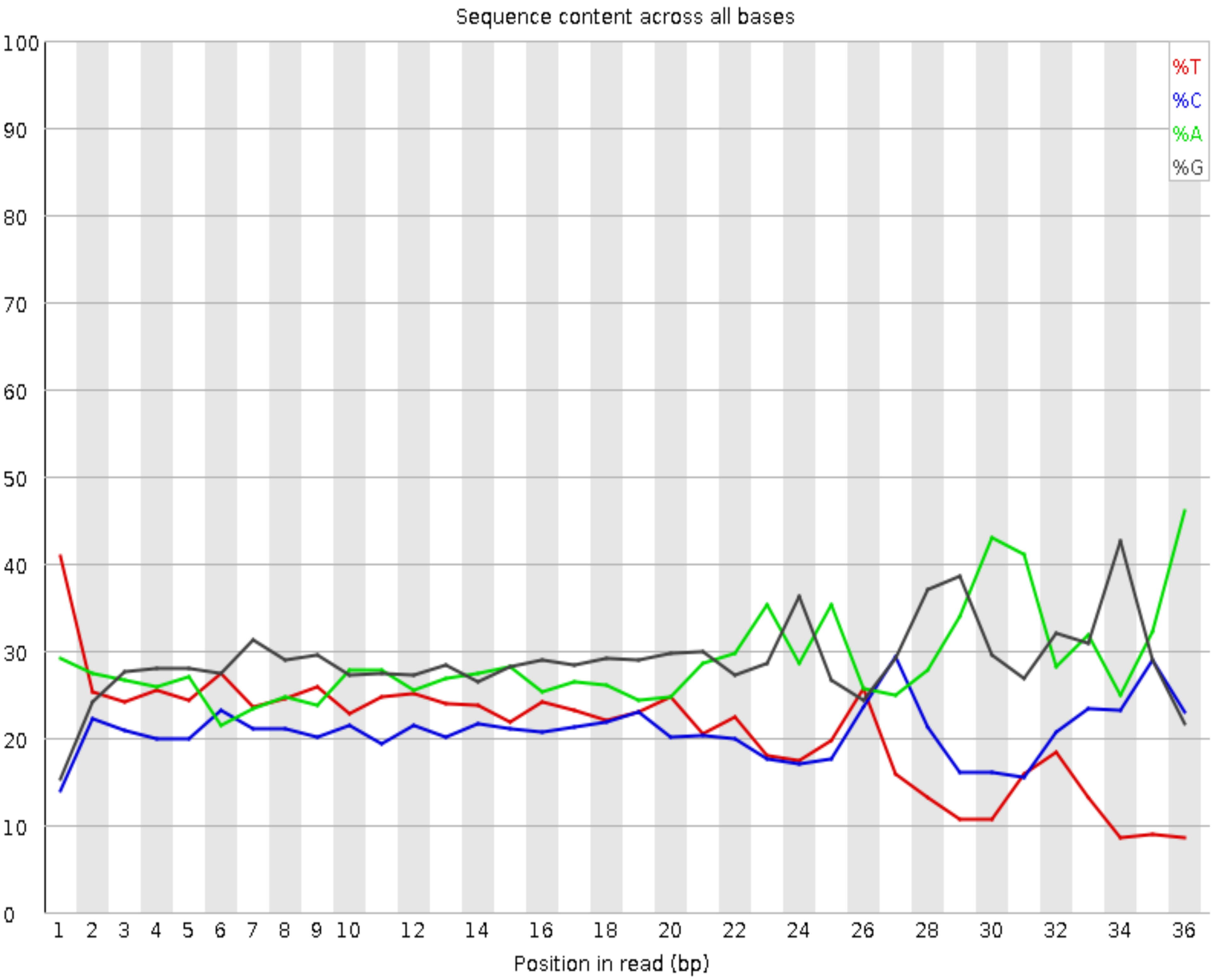

Per sequence GC content

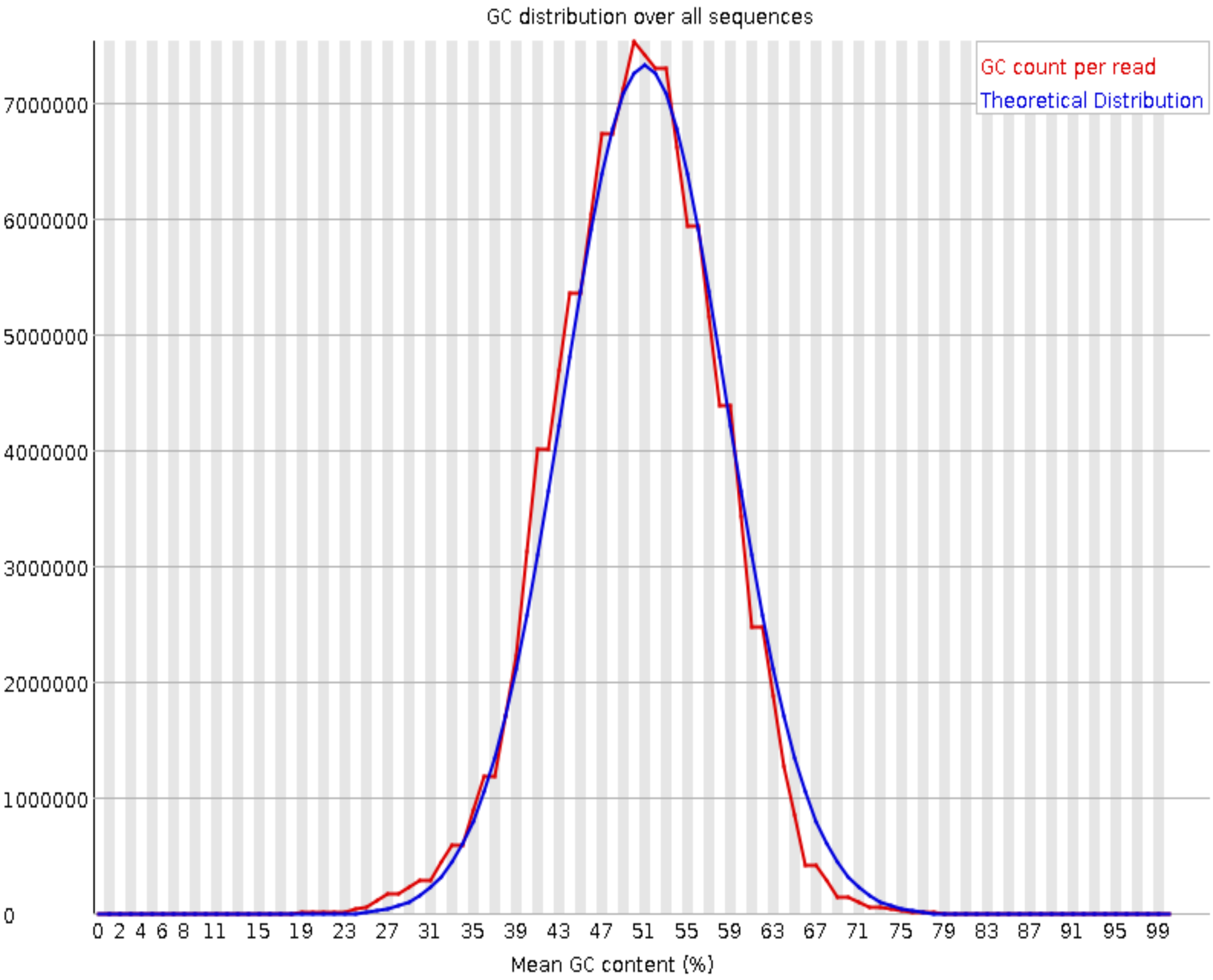

Per base N content

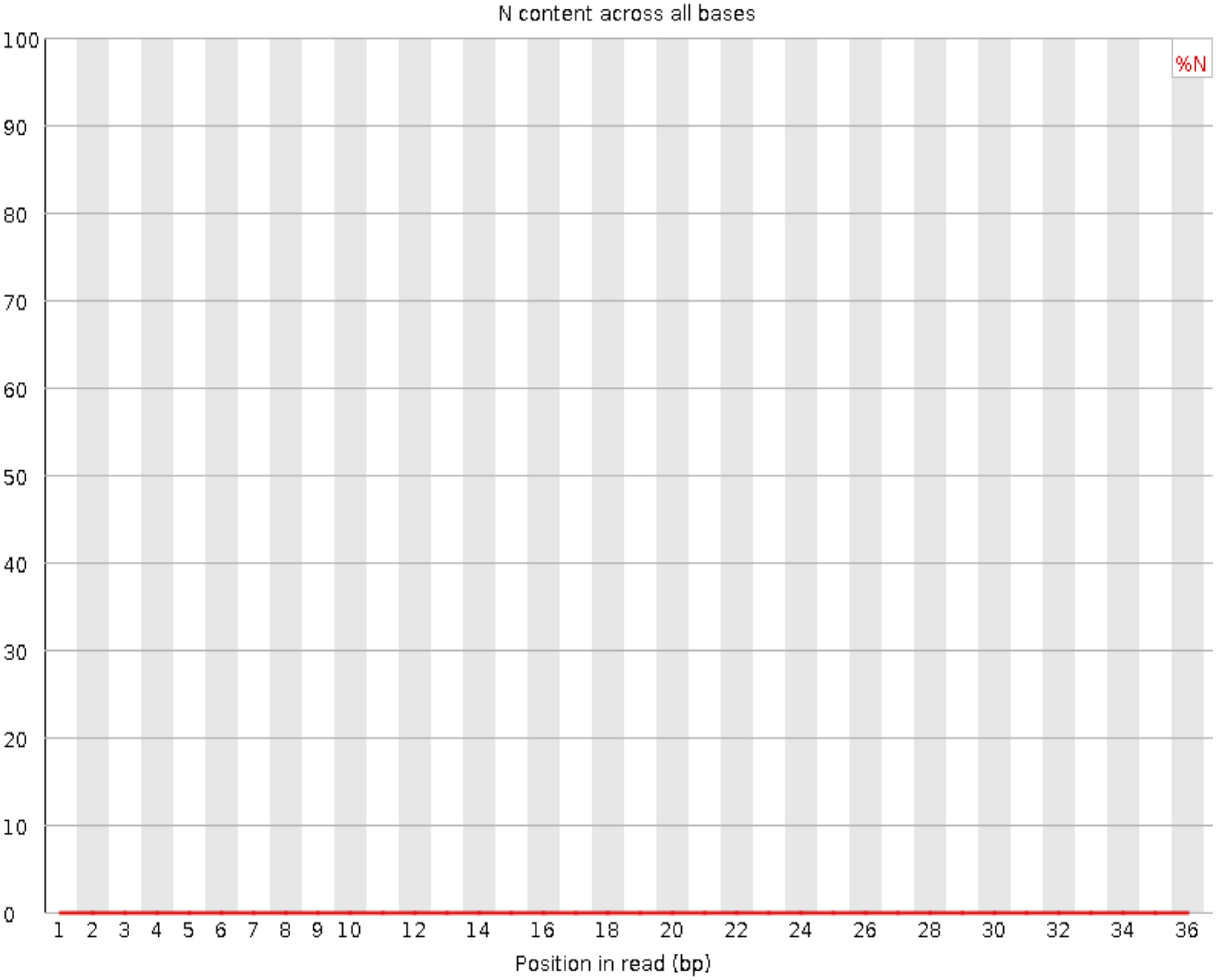

Sequence Length Distribution

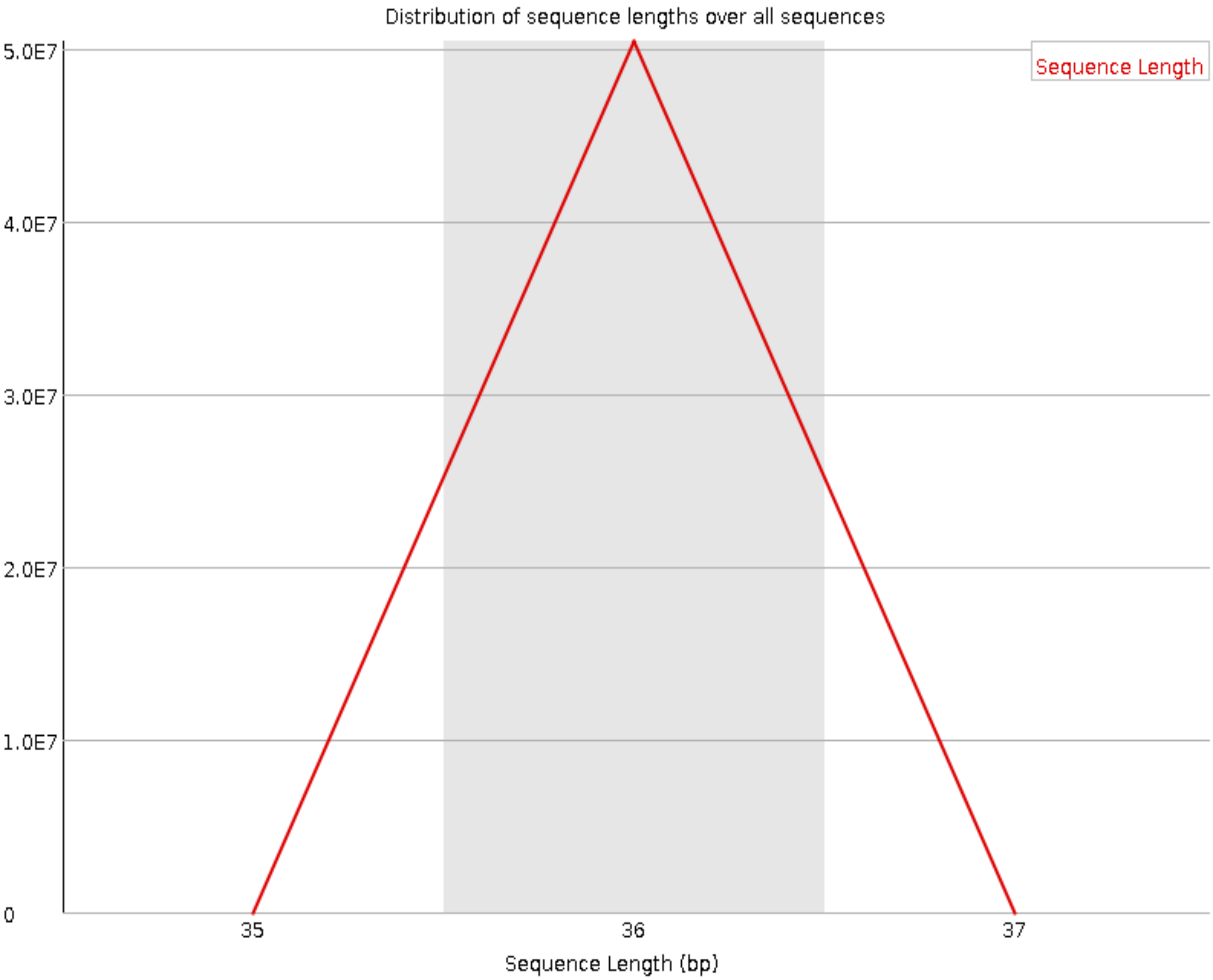

Sequence Duplication Levels

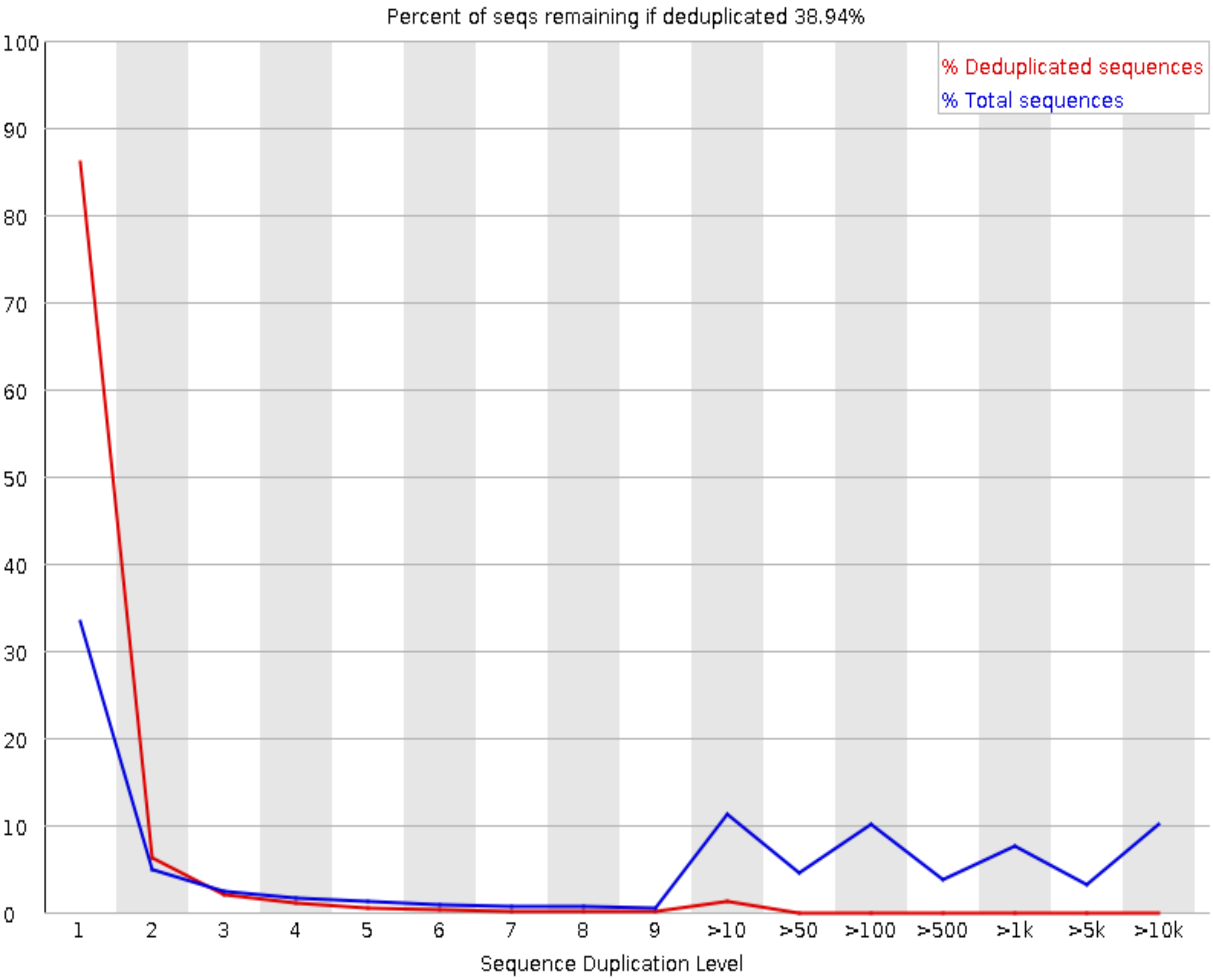

Overrepresented sequences

Sequence	Count	Percentage	Possible Source
ATTTGTGGTAGTGGGGACTGCGTTCGCGCTTTCCC	297419	0.5898862353901911	No Hit
ACCTGGGAACACGGAGTGCTGATGGCATAGATCGGA	235093	0.46627190844090727	No Hit
TGGAATGTAAAGAAGTATGGAGAGATCGGAAGAGCA	137016	0.2717508041793646	No Hit
TAGATCGGAAGAGCACACGTCTGAACTCCAGTCACG	118091	0.23421588877463465	Illumina Multiplexing PCR Primer 2.01 (100% over 34bp)
CAATTTTTGGTAGTGGGGACTGCGTTCGCGCTATC	104174	0.2066135945771379	No Hit
CAGATCGGAAGAGCACACGTCTGAACTCCAGTCACG	98277	0.19491777443755046	Illumina Multiplexing PCR Primer 2.01 (100% over 34bp)

ATGGGAGACCGCCTGGGAATACCGGGTGCTGTAGGC	93483	0.18540959031864557	No Hit
TGAGATCATTGTGAAAGCTGATTAGATCGGAAGAGC	89485	0.17748015350025134	No Hit
GACCGCCTGGGAATACCGGGTGCTGTAGGCTTAGAT	76045	0.15082391767253298	No Hit
ACCTGGGAACACGGAGTGCTGATGGCACAGATCGGA	76016	0.15076640049701187	No Hit
CCGCCTGGGAATACCGGGTGCTGTAGGCTTAGATCG	70194	0.13921933167342732	No Hit
ACCTGGGAACACGGAGTGCTGATGGCAAGATCGGAA	67613	0.1341003030520478	No Hit
TGGAATGTAAAGAAGTATGGAAGATCGGAAGAGCAC	67186	0.1332534122262713	No Hit
ATAGATCGGAAGAGCACACGTCTGAACTCCAGTCAC	60882	0.12075036827850817	Illumina Multiplexing PCR Primer 2.01 (100% over 34bp)
AAGATCGGAAGAGCACACGTCTGAACTCCAGTCACG	59958	0.11891775206535253	Illumina Multiplexing PCR Primer 2.01 (100% over 34bp)
TGACTAGATCCACACTCATCCAAGATCGGAAGAGCA	58926	0.11687093395715271	No Hit
CTTTGCAAGATCTGATCAGATCGGAAGAGCACACGT	58602	0.11622832827202022	No Hit
ACCTGGGAACACGGAGTGCTGATGGCAGATCGGAAG	58202	0.11543498792000478	No Hit

Adapter Content

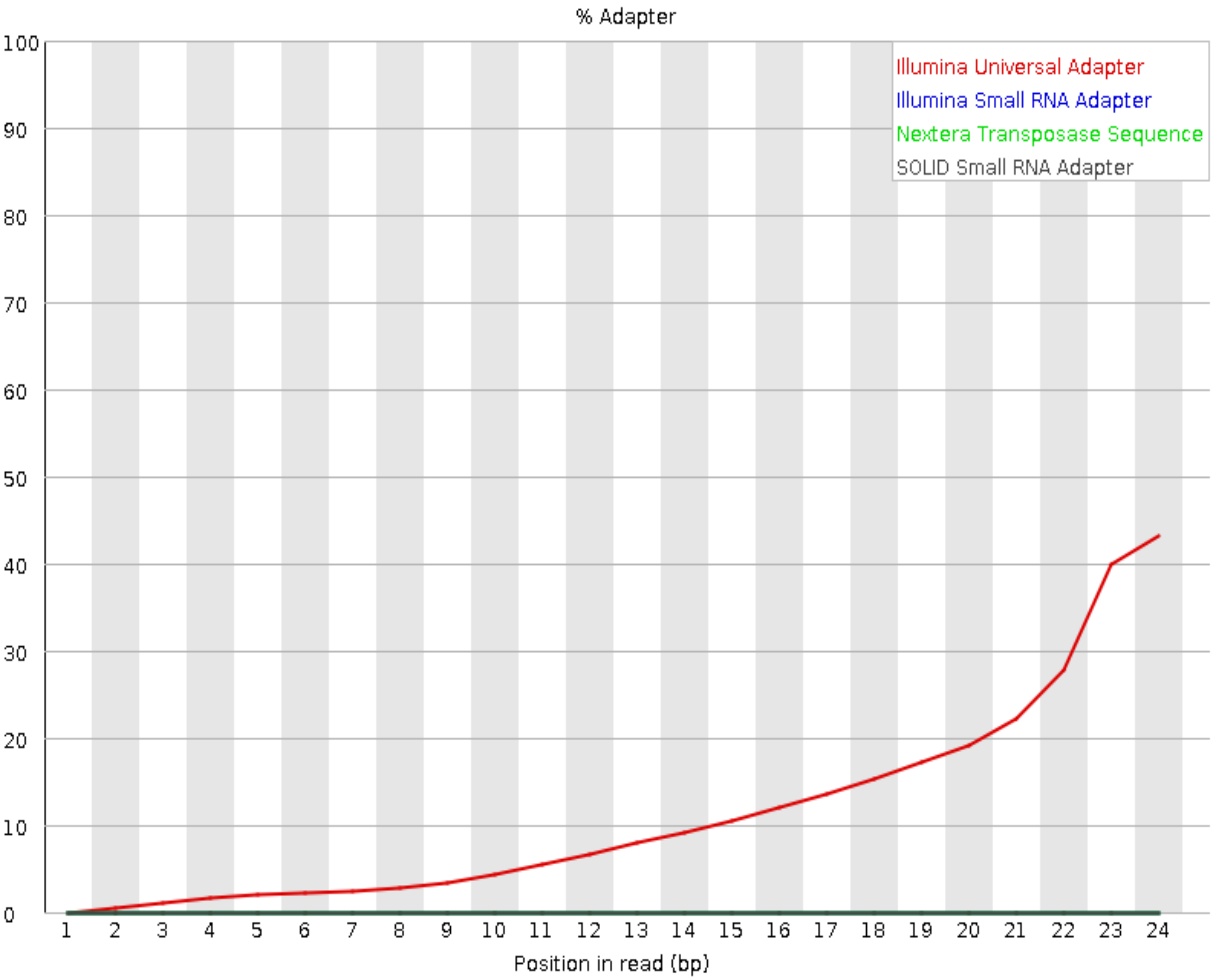

Kmer Content

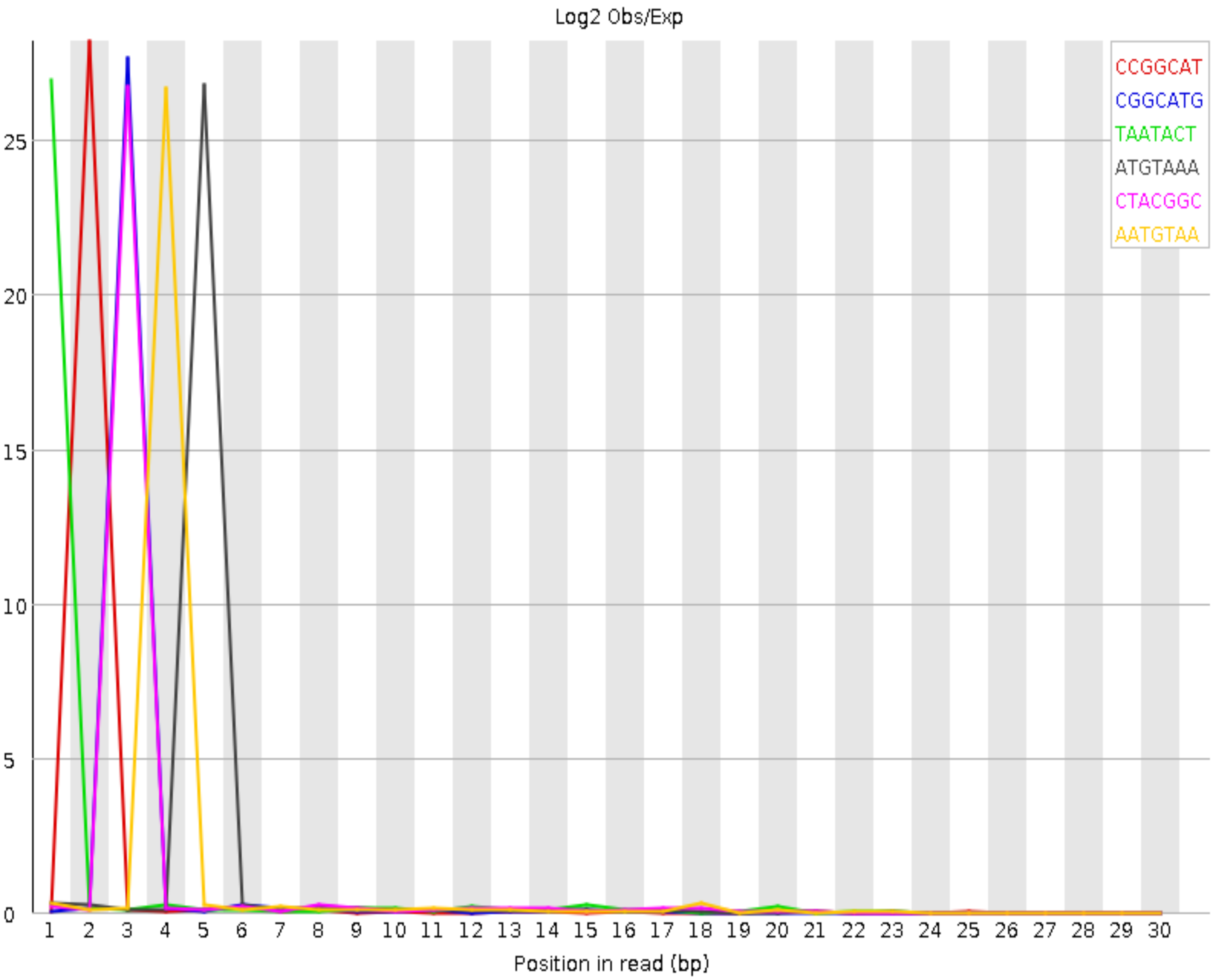

Sequence	Count	PValue	Obs/Exp Max	Max Obs/Exp Position
CCGGCAT	24065	0.0	28.181135	2
CGGCATG	23970	0.0	27.685678	3
TAATACT	13715	0.0	26.985329	1
ATGTAAA	47310	0.0	26.772886	5
CTACGGC	11305	0.0	26.76353	3
AATGTAA	48230	0.0	26.681927	4
GCATGGT	22055	0.0	26.477518	5
GGCATGG	23325	0.0	26.386248	4
AGTATGG	45155	0.0	26.325993	14
CGGCCAT	31710	0.0	26.187948	6
AACCCGT	17440	0.0	26.04932	1
CTCATCC	16855	0.0	26.047827	15
AAGTATG	47545	0.0	26.04047	13

GGAATGT	50555	0.0	26.040081	2
AGGTAAA	14860	0.0	25.981913	11
CACTCAT	21405	0.0	25.935406	13
CAGGTAA	14880	0.0	25.86635	10
GAATGTA	50940	0.0	25.825502	3
ATCATTG	19890	0.0	25.769745	5
TGTAAAG	49595	0.0	25.74817	6

Report

Summary

- Basic Statistics
- Per base sequence quality
- Per tile sequence quality
- Per sequence quality scores
- Per base sequence content
- Per sequence GC content
- Per base N content
- Sequence Length Distribution
- Sequence Duplication Levels
- Overrepresented sequences
- Adapter Content
- Kmer Content

Basic Statistics

Measure	Value
Filename	ISE6-NY5_R1.fastq
File type	Conventional base calls
Encoding	Sanger / Illumina 1.9
Total Sequences	35765024
Sequences flagged as poor quality	0
Sequence length	36
%GC	49

Per base sequence quality

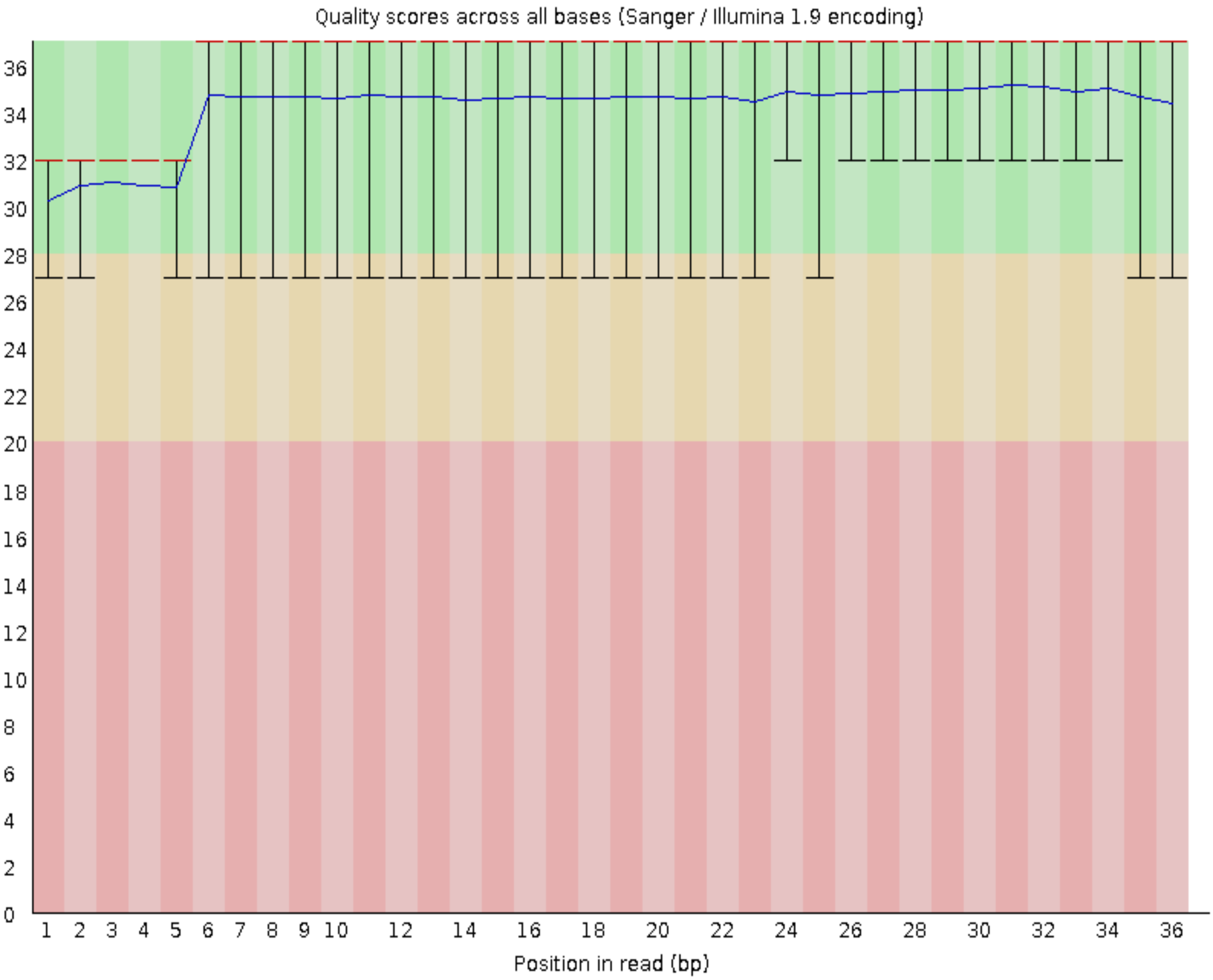

Per tile sequence quality

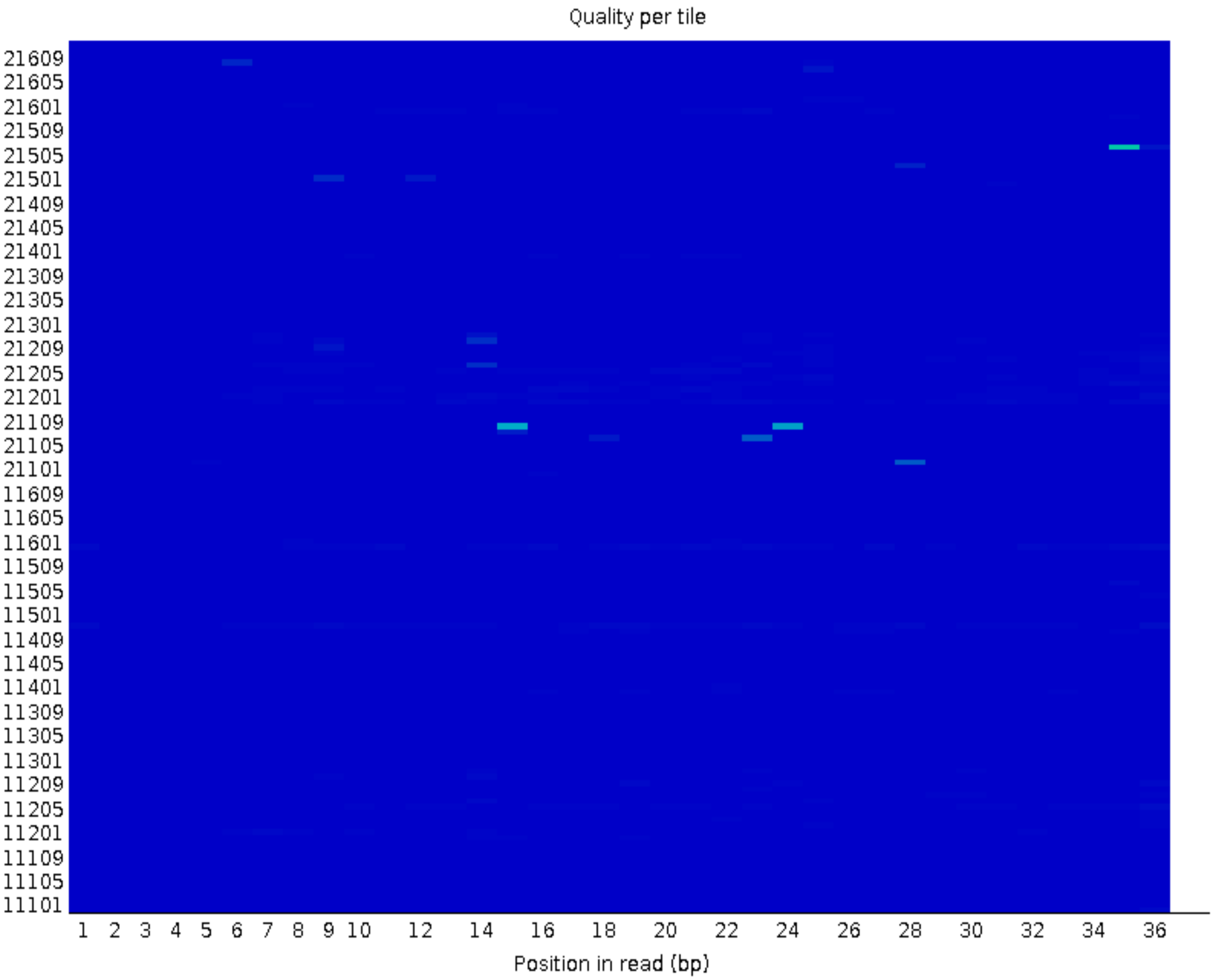

Per sequence quality scores

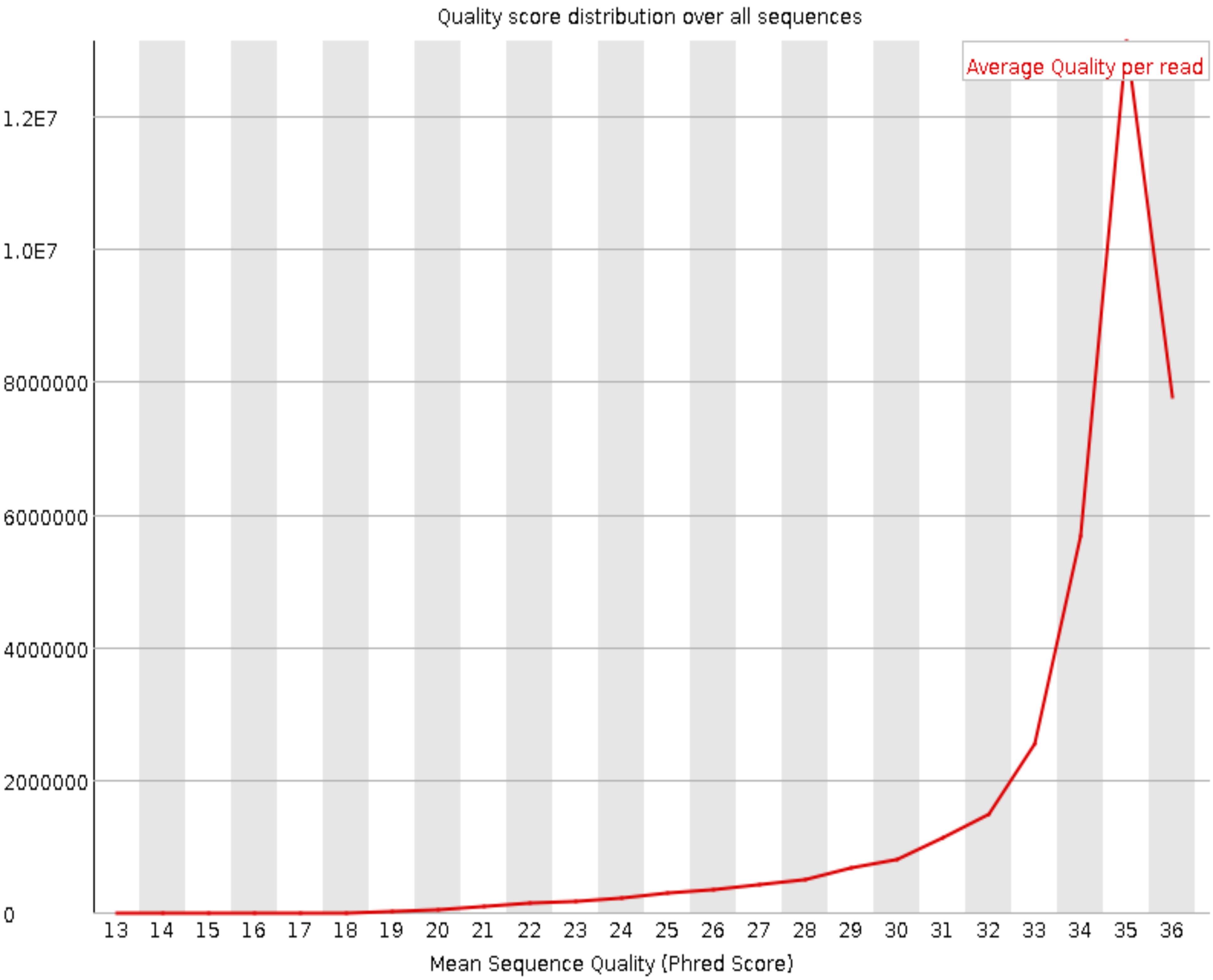

Per base sequence content

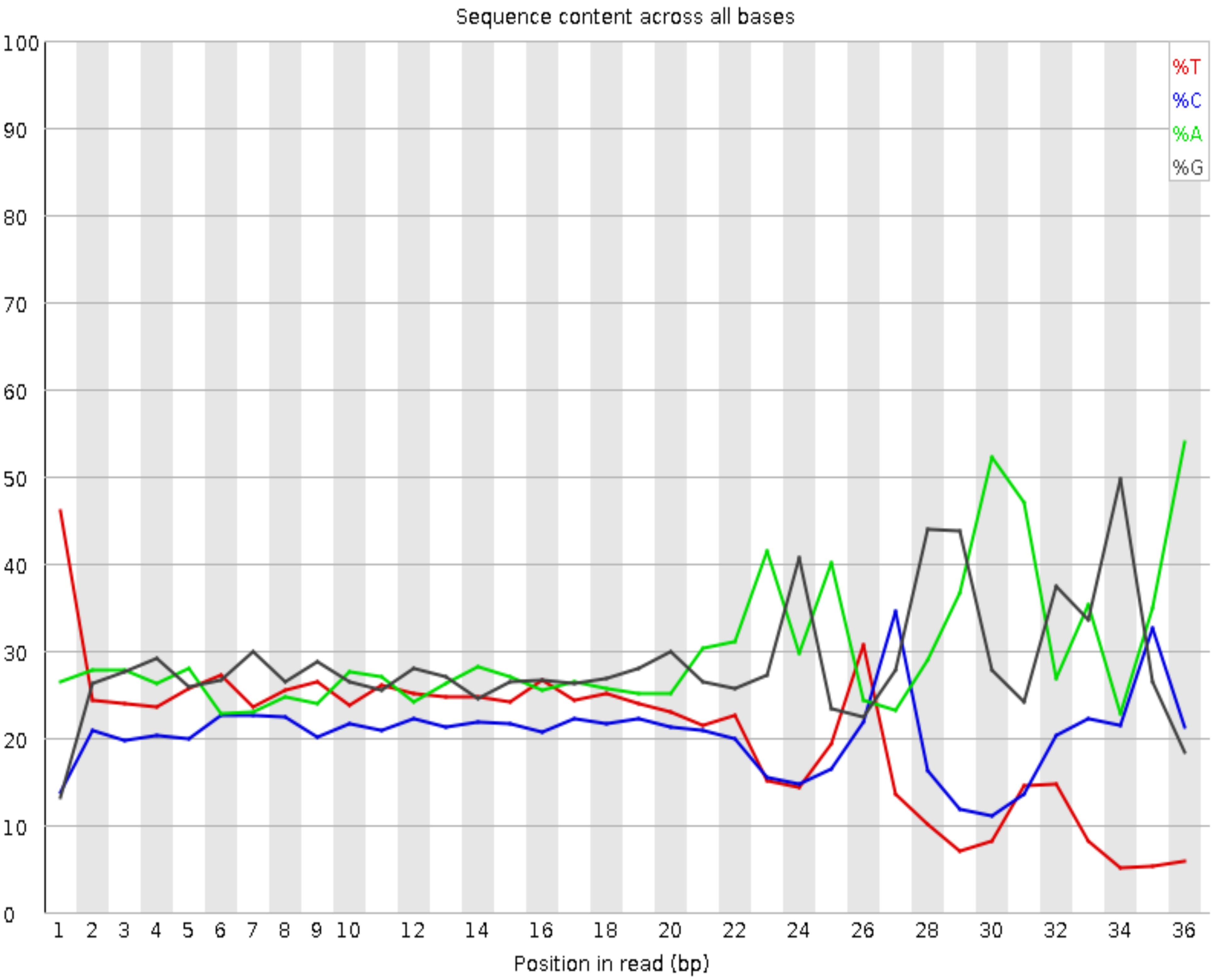

Per sequence GC content

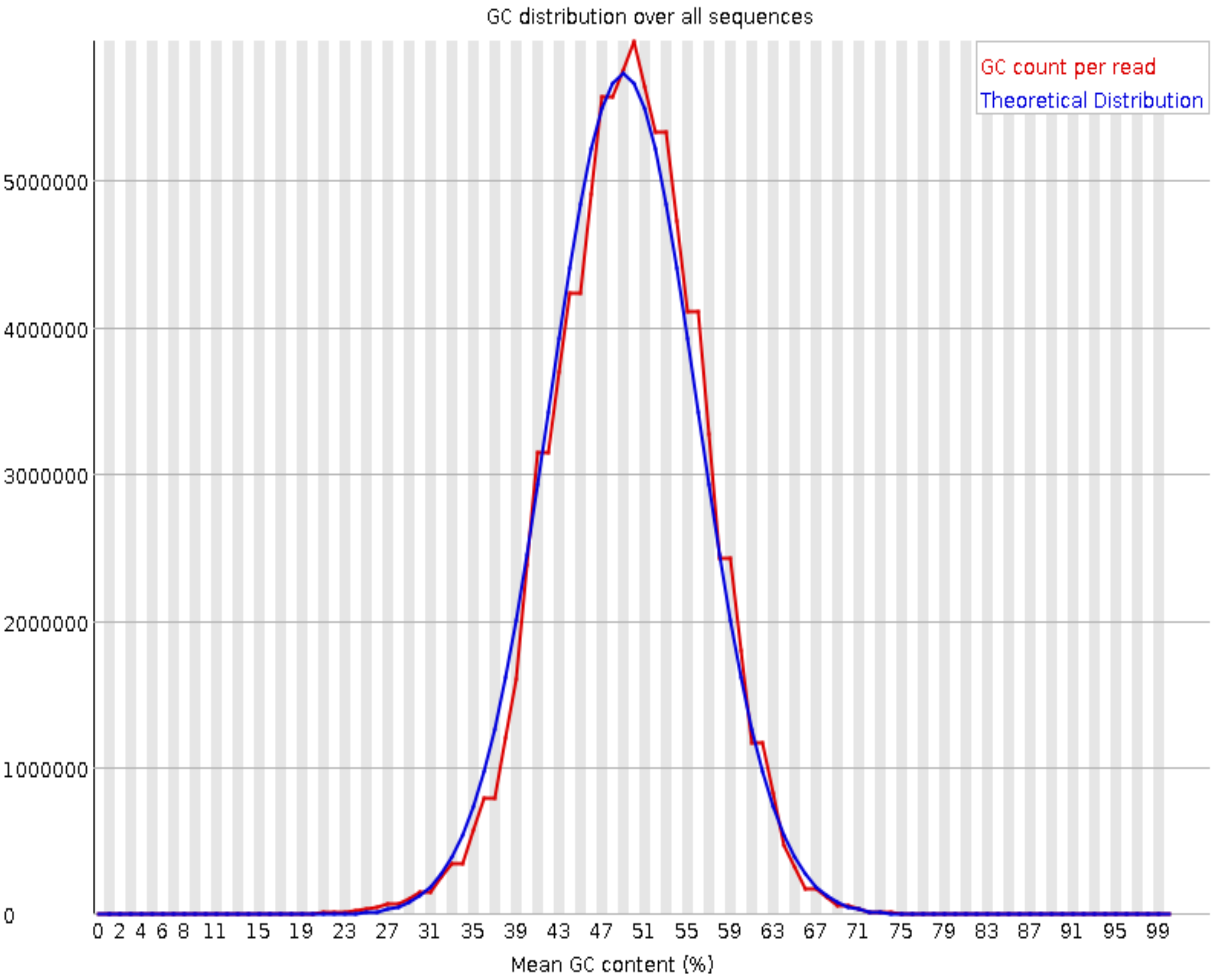

Per base N content

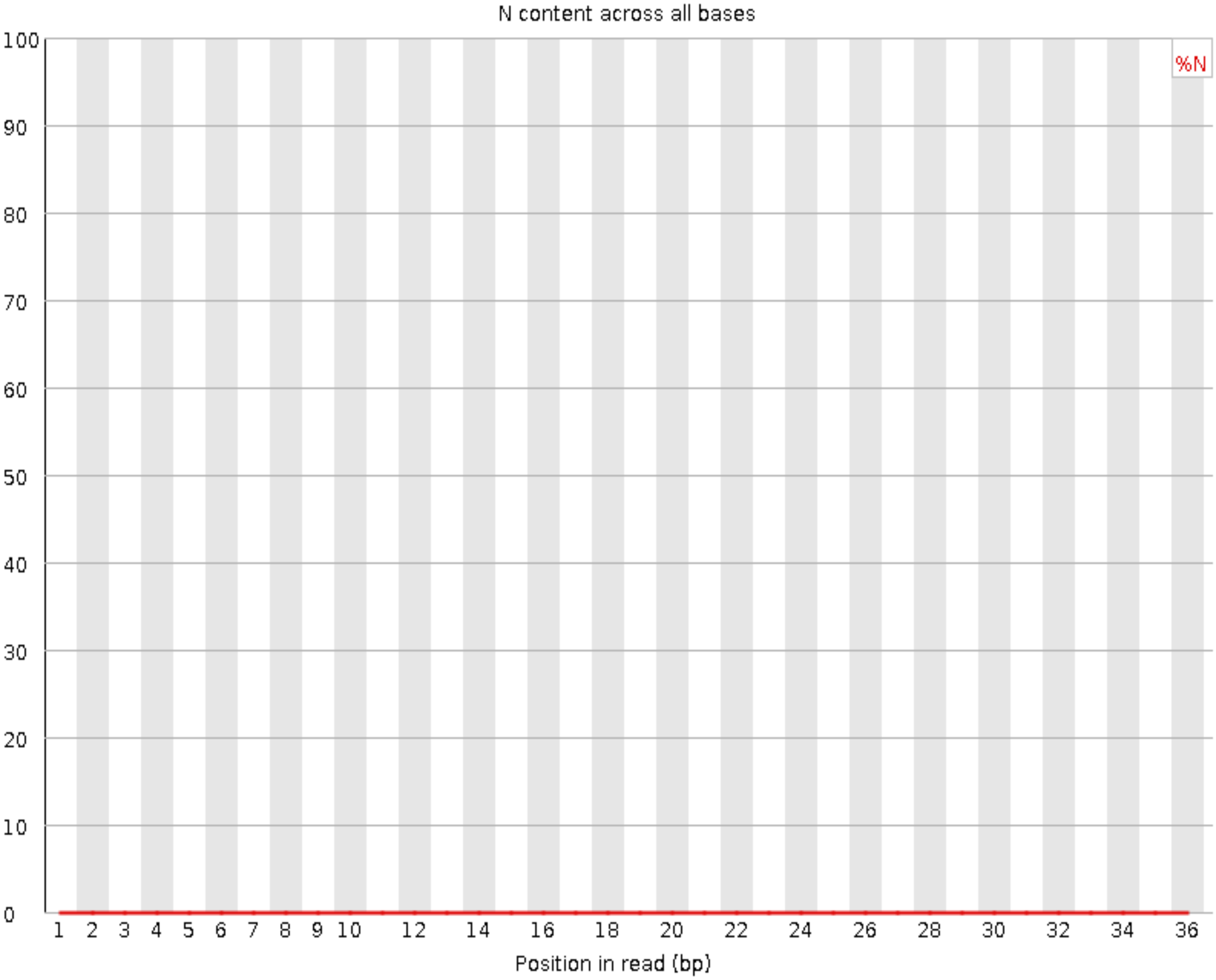

Sequence Length Distribution

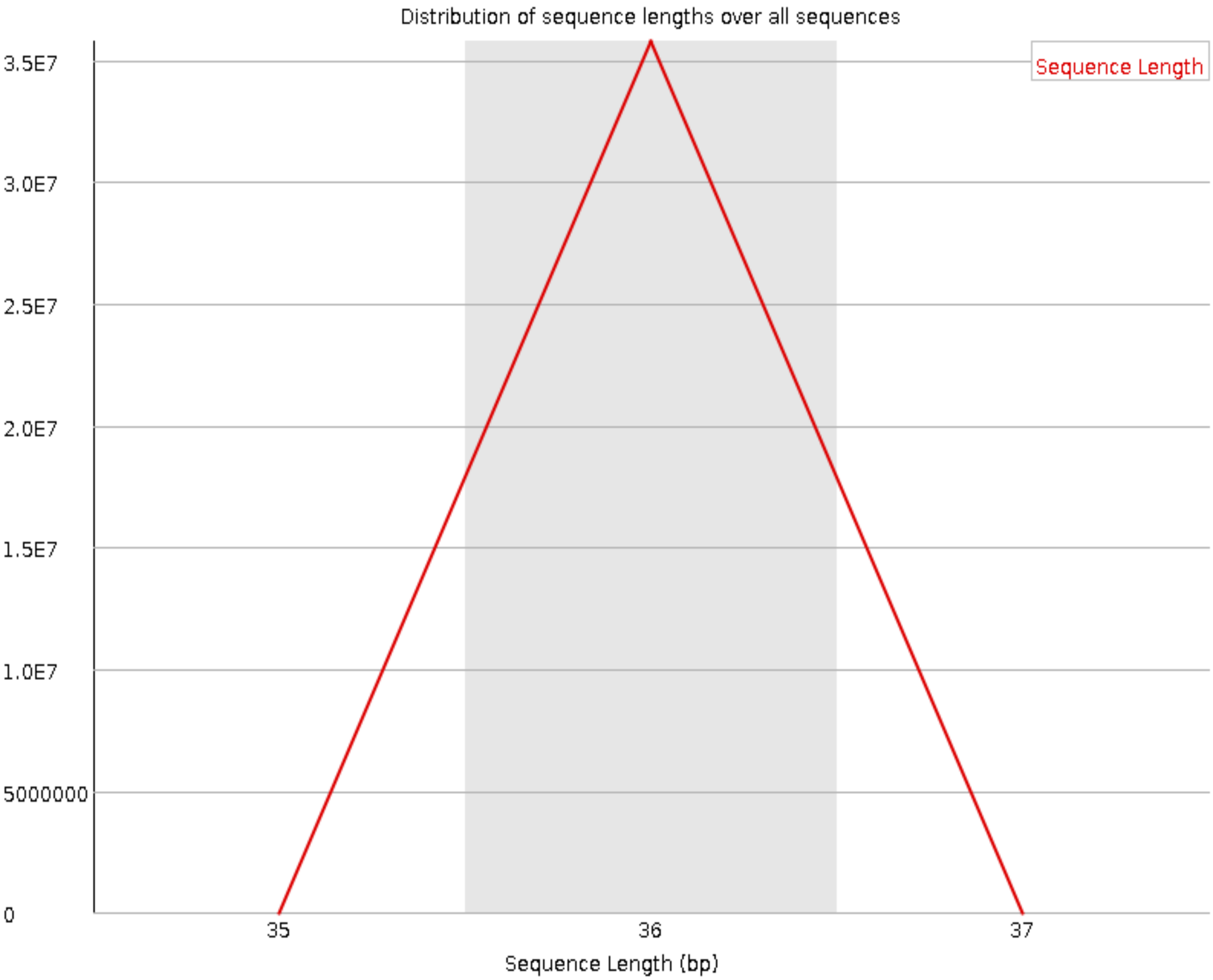

Sequence Duplication Levels

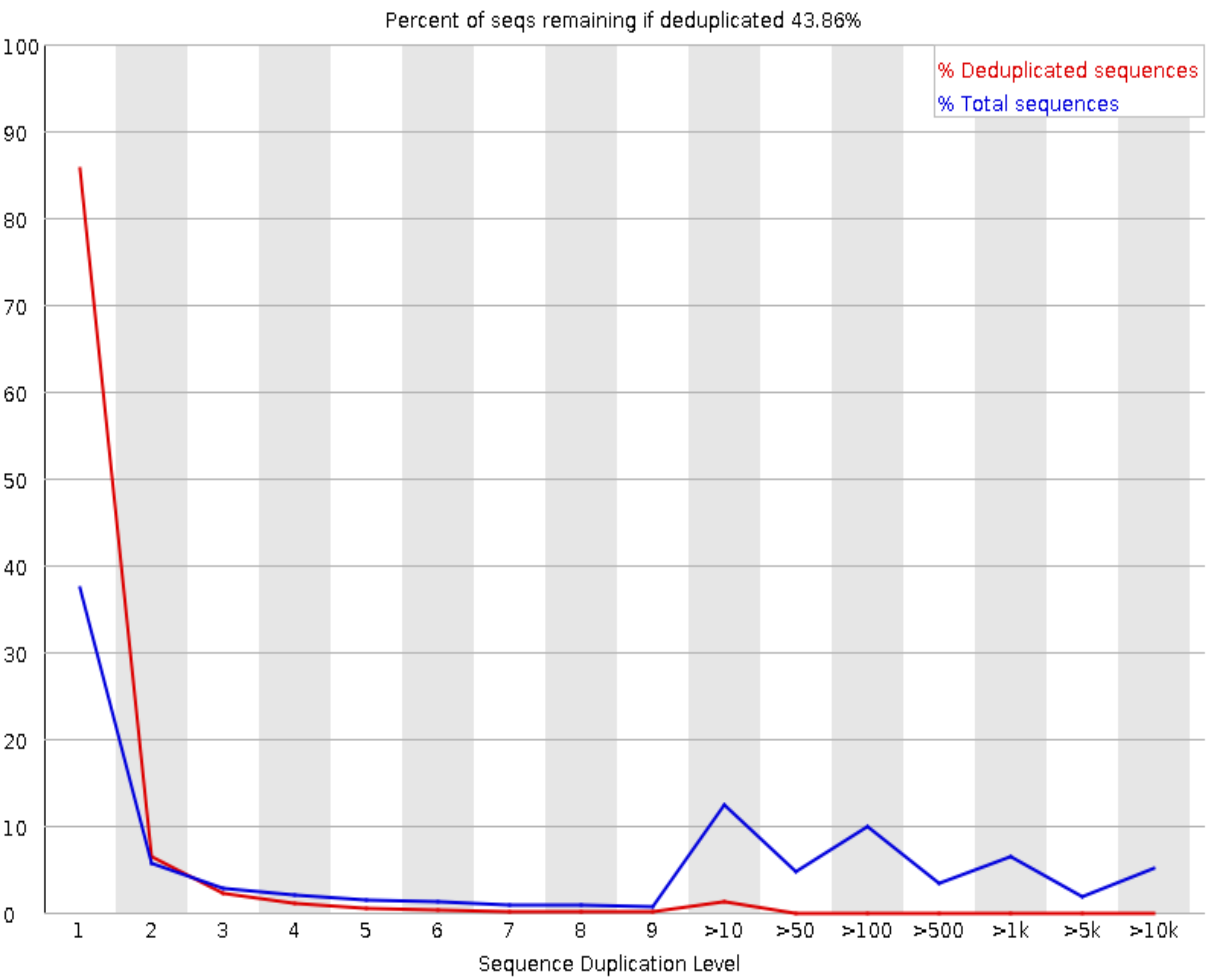

Overrepresented sequences

Sequence	Count	Percentage	Possible Source
TGGAATGTAAAGAAGTATGGAGAGATCGGAAGAGCA	169880	0.474989196148729	No Hit
TGAGATCATTGTGAAAGCTGATTAGATCGGAAGAGC	90687	0.2535633696205544	No Hit
CTTTGCAAGATCTGATCAGATCGGAAGAGCACACGT	77602	0.21697734635939292	No Hit
ACCTGGGAACACGGAGTGCTGATGGCATAGATCGGA	72655	0.2031453970225212	No Hit
TGACTAGATCCACACTCATCCAAGATCGGAAGAGCA	66863	0.18695080422705712	No Hit
TGGAATGTAAAGAAGTATGGAAGATCGGAAGAGCAC	64341	0.17989922221218138	No Hit
AACCCGTAGATCCGAACTTGTGAGATCGGAAGAGCA	56737	0.15863822711261147	No Hit
CAATTTTTGGTAGTAGATCGGAAGAGCACACGTCTG	47432	0.13262118879047866	Illumina Multiplexing PCR Primer 2.01 (100% over 22bp)

TGACTAGATCCACACTCATCCAGATCGGAAGAGCAC	46931	0.1312203788818931	No Hit
AGGGTCGGATAGATCGGAAGAGCACACGTCTGAACT	44530	0.12450711622617673	Illumina Multiplexing PCR Primer 2.01 (100% over 26bp)
TAGATCGGAAGAGCACACGTCTGAACTCCAGTCACG	42288	0.11823842198456236	Illumina Multiplexing PCR Primer 2.01 (100% over 34bp)
GATAACACGGACAATGGTGTAACGGTAGATCGGAAG	39664	0.11090164513799851	No Hit
AACCCGTAGATCCGAACTTGTAGATCGGAAGAGCAC	36415	0.10181735094040478	No Hit

Adapter Content

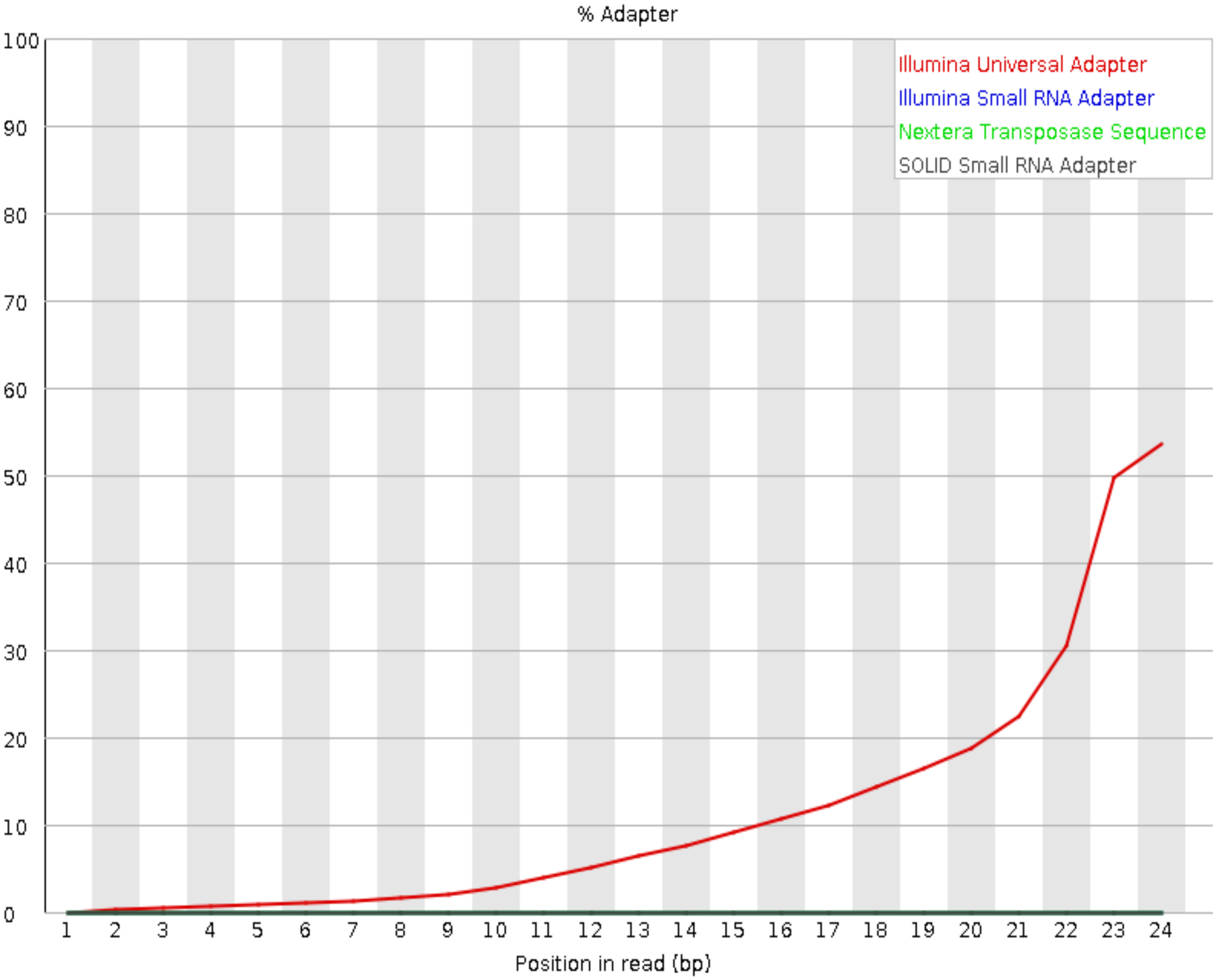

Kmer Content

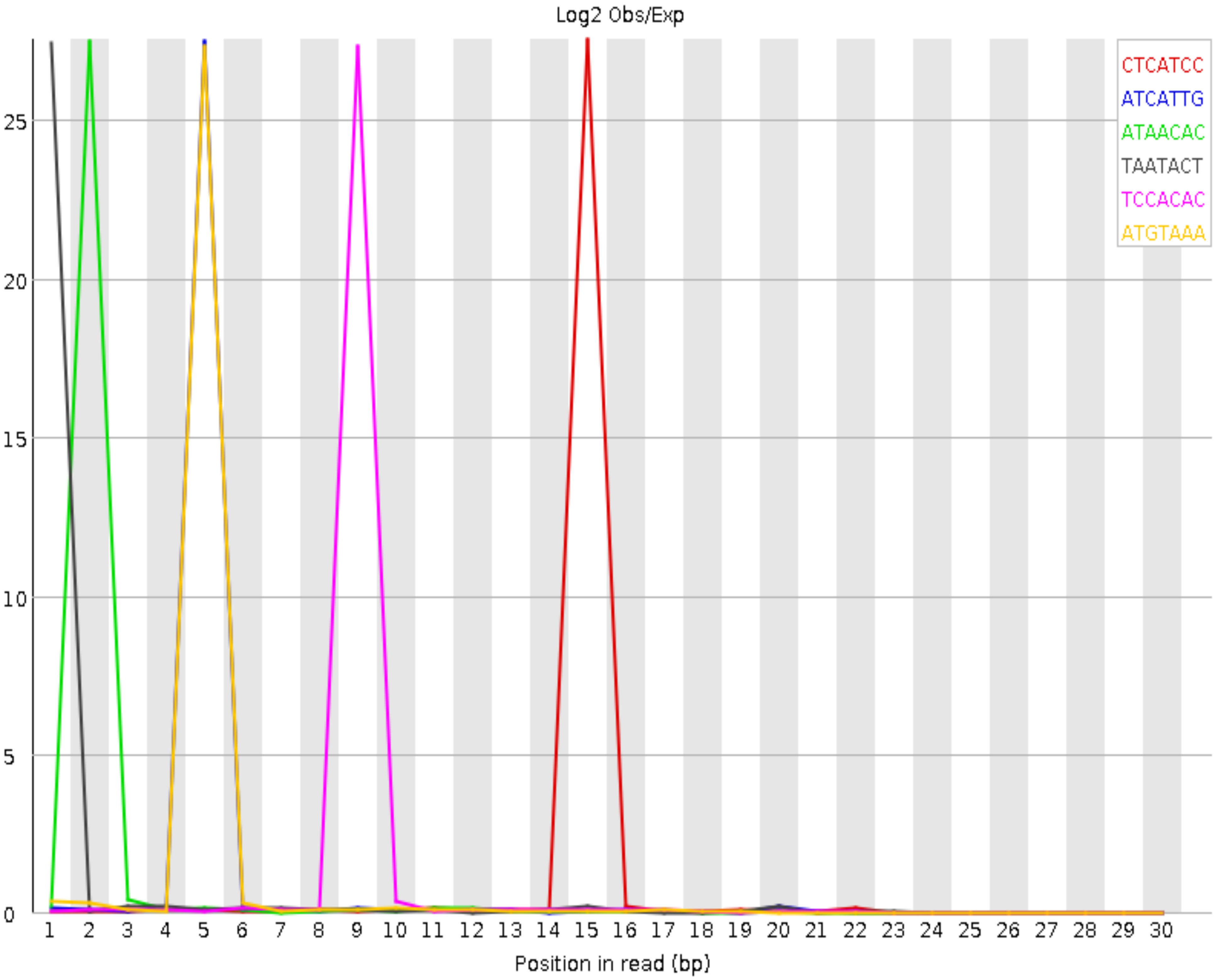

Sequence	Count	PValue	Obs/Exp Max	Max Obs/Exp Position
CTCATCC	19740	0.0	27.521591	15
ATCATTG	19610	0.0	27.499828	5
ATAACAC	15450	0.0	27.497097	2
TAATACT	12875	0.0	27.429806	1
TCCACAC	26900	0.0	27.344414	9
ATGTAAA	48445	0.0	27.322802	5
AATGTAA	49430	0.0	27.212337	4
CACTCAT	25800	0.0	27.179806	13
CCACACT	26545	0.0	27.156738	10
ACCCGTA	19245	0.0	27.149239	2

AACCCGT	19130	0.0	27.146208	1
AGTATGG	46175	0.0	26.968788	14
CAGGTAA	13640	0.0	26.919909	10
GATCATT	21340	0.0	26.88022	4
GAATGTA	50970	0.0	26.864653	3
AGGTAAA	13865	0.0	26.710238	11
GGAATGT	51405	0.0	26.649103	2
TGTAAAG	50100	0.0	26.591	6
AAGTATG	48820	0.0	26.389383	13
GATCCAC	28575	0.0	26.385195	7

Produced by FastQC (version 0.11.3)

Report

Fri 3 Apr 2015
ISE6-U2_R1.fastq

Summary

- Basic Statistics
- Per base sequence quality
- Per tile sequence quality
- Per sequence quality scores
- Per base sequence content
- Per sequence GC content
- Per base N content
- Sequence Length Distribution
- Sequence Duplication Levels
- Overrepresented sequences
- Adapter Content
- Kmer Content

Basic Statistics

Measure	Value
Filename	ISE6-U2_R1.fastq
File type	Conventional base calls
Encoding	Sanger / Illumina 1.9
Total Sequences	42027955
Sequences flagged as poor quality	0
Sequence length	36
%GC	49

Per base sequence quality

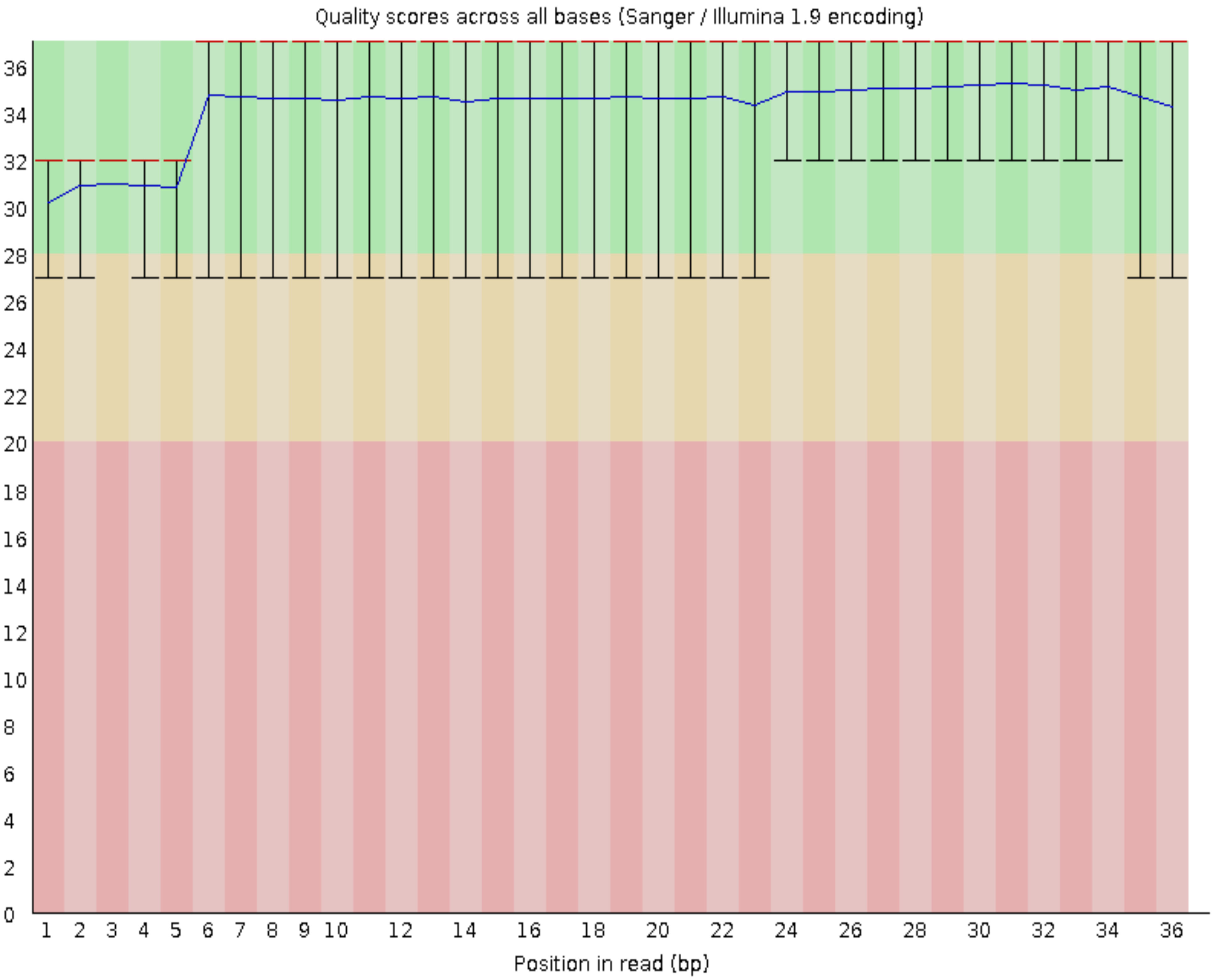

Per tile sequence quality

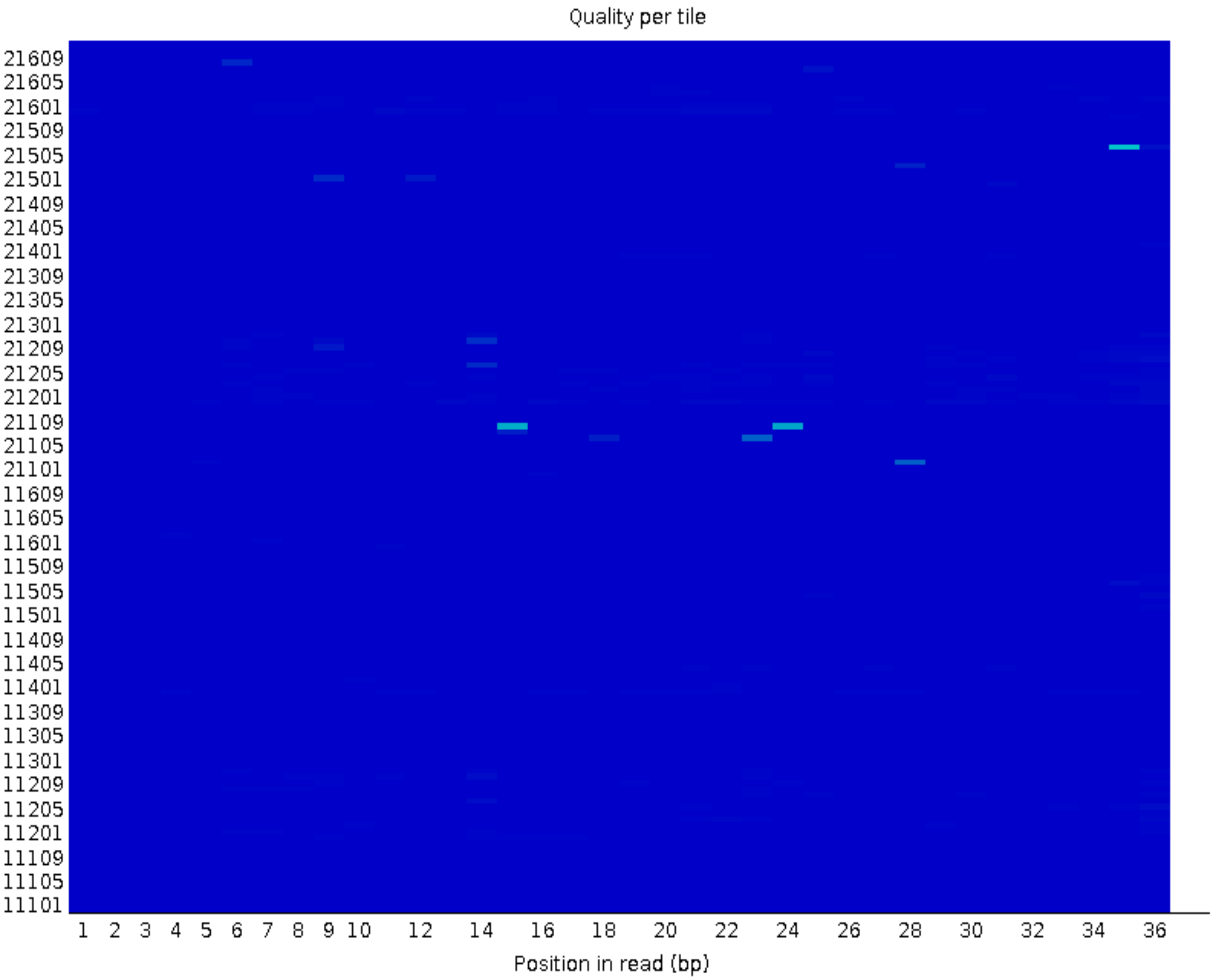

Per sequence quality scores

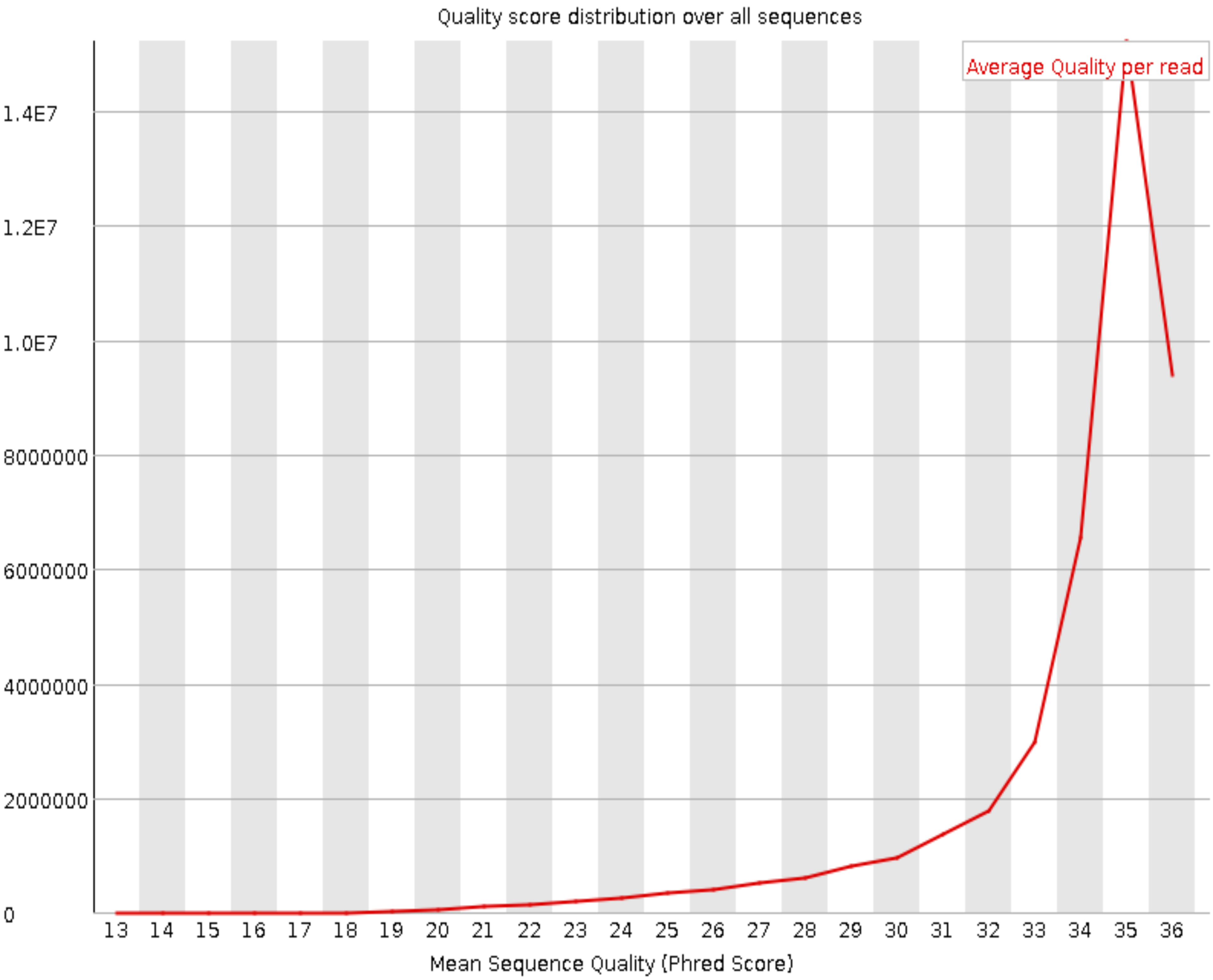

Per base sequence content

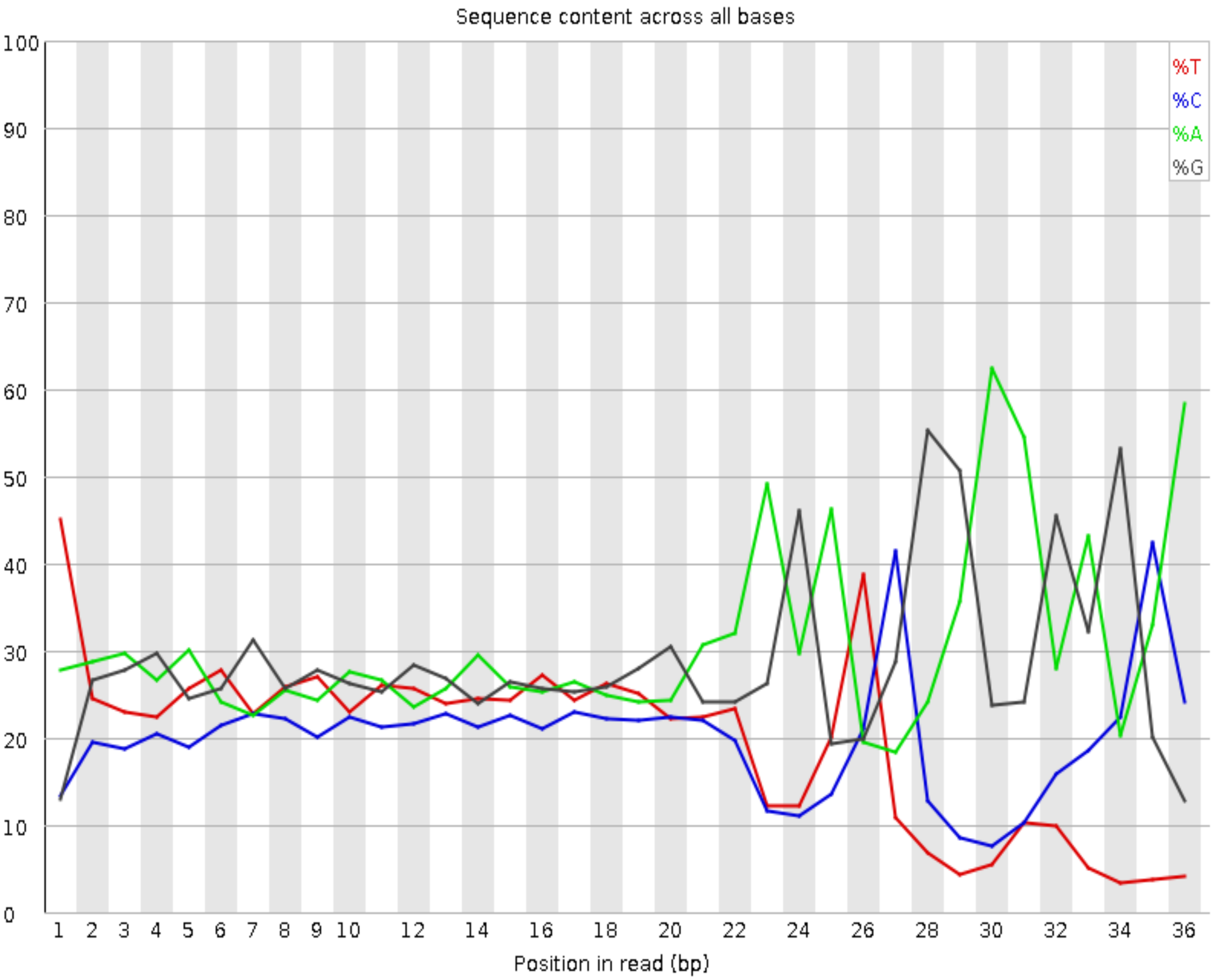

Per sequence GC content

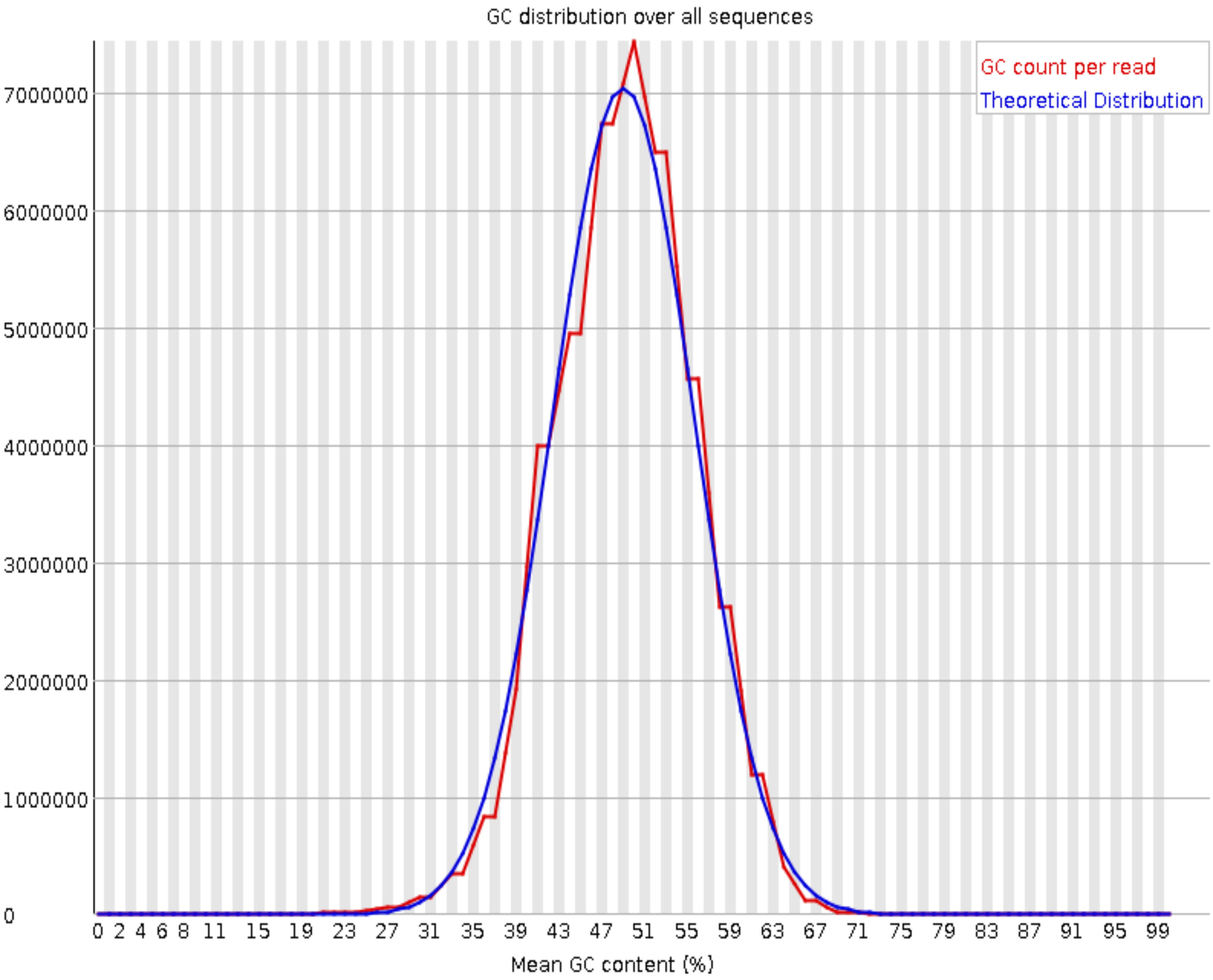

Per base N content

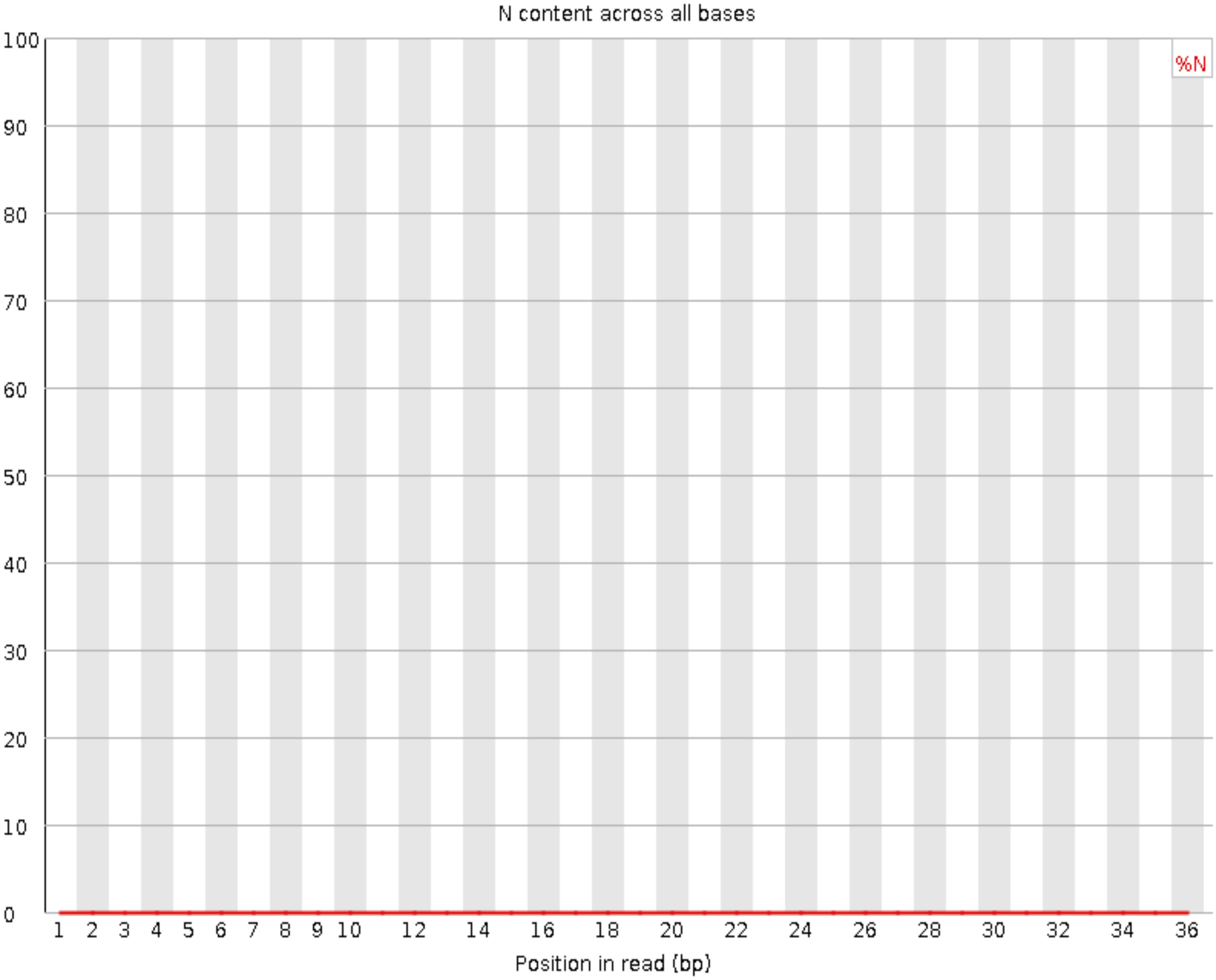

Sequence Length Distribution

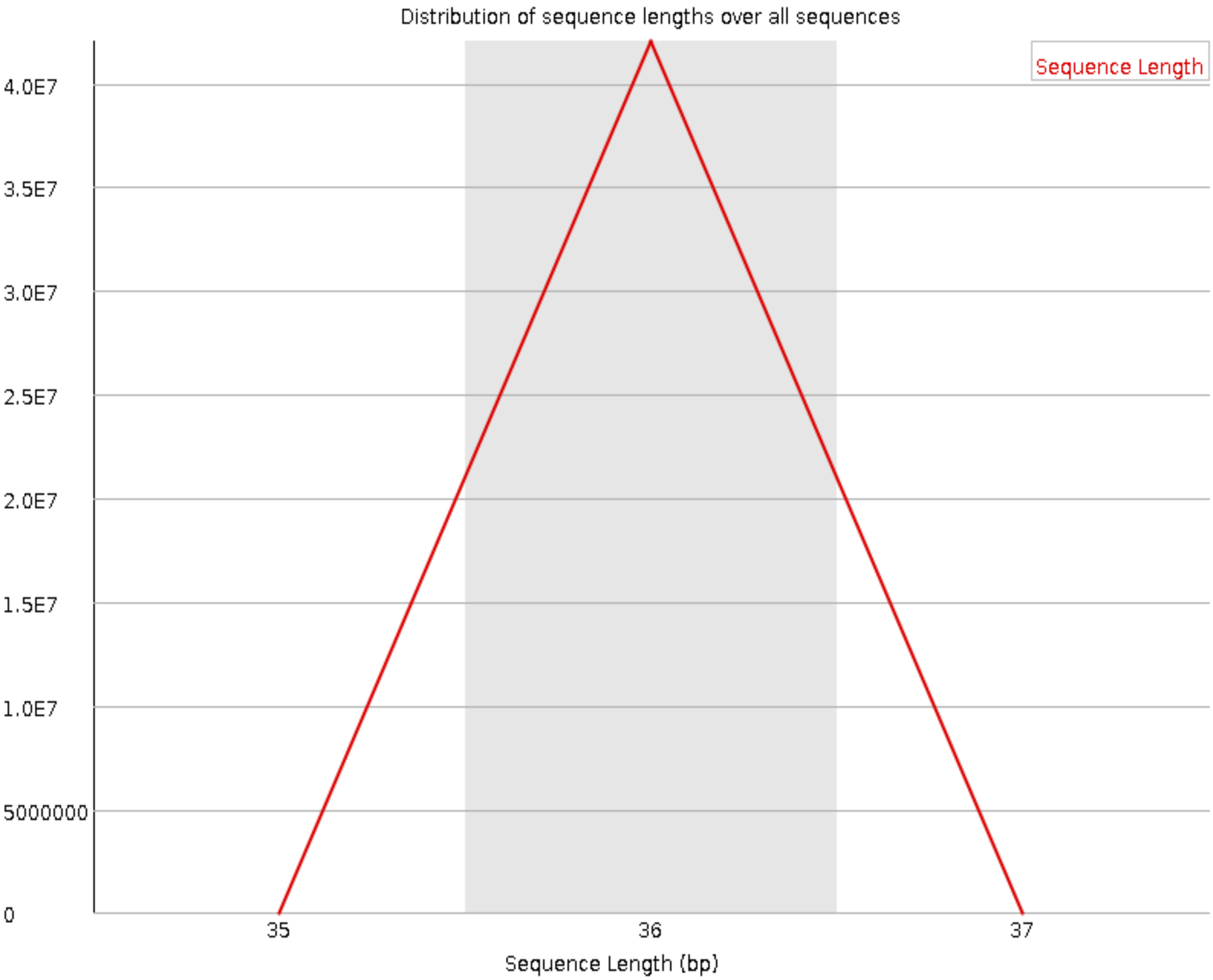

Sequence Duplication Levels

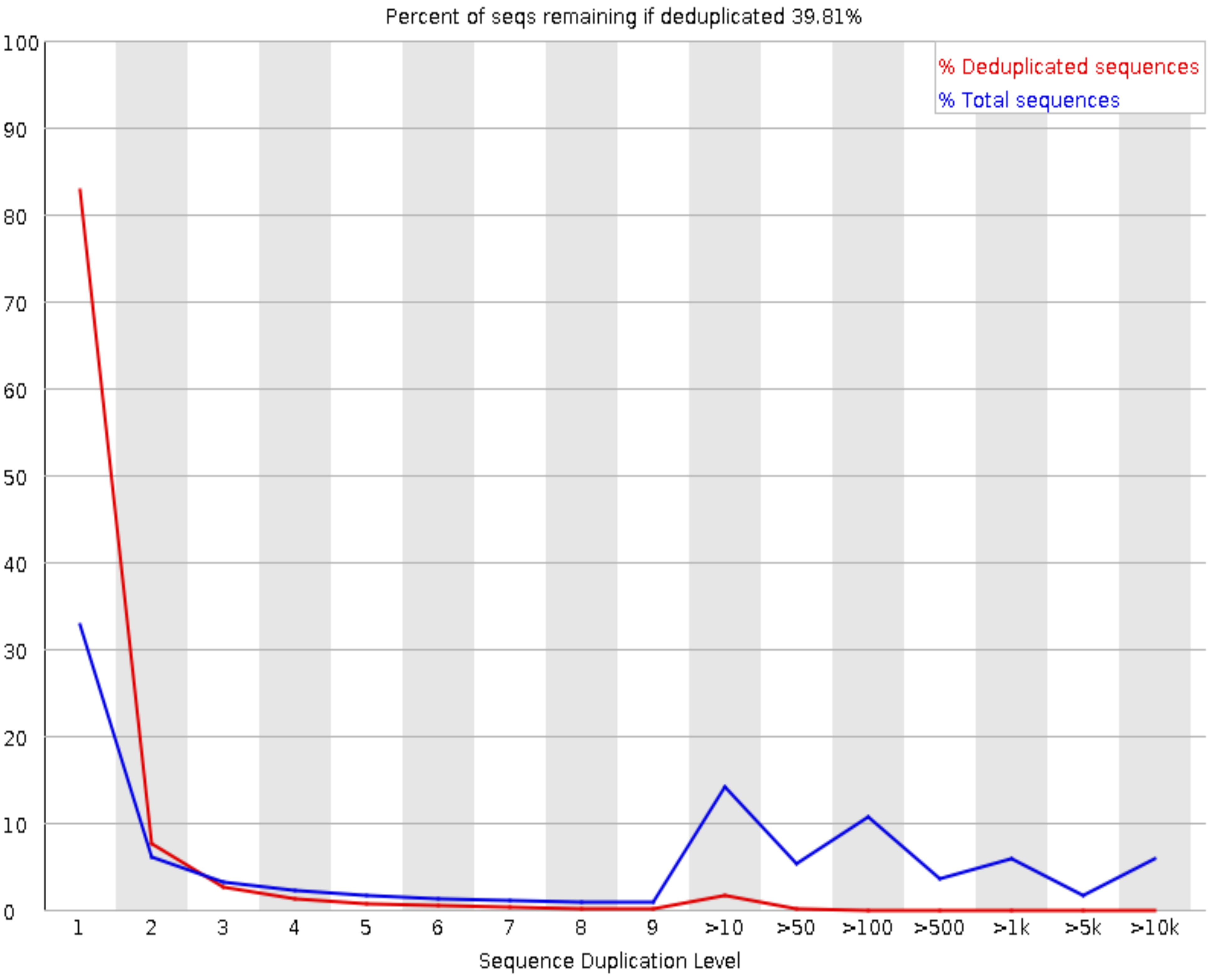

Overrepresented sequences

Sequence	Count	Percentage	Possible Source
TGGAATGTAAAGAAGTATGGAGAGATCGGAAGAGCA	318687	0.75827386795289	No Hit
TGAGATCATTGTGAAAGCTGATTAGATCGGAAGAGC	172352	0.41008895150858515	No Hit
TGACTAGATCCACACTCATCCAAGATCGGAAGAGCA	146748	0.3491675957109976	No Hit
TGGAATGTAAAGAAGTATGGAAGATCGGAAGAGCAC	128825	0.3065221707789494	No Hit
TGACTAGATCCACACTCATCCAGATCGGAAGAGCAC	112225	0.26702465061647657	No Hit
AACCCGTAGATCCGAACTTGTGAGATCGGAAGAGCA	88285	0.21006256430987422	No Hit
AACCCGTAGATCCGAACTTGTAGATCGGAAGAGCAC	79176	0.1883888949628884	No Hit
TGGAATGTAAAGAAGTATGGAGATCGGAAGAGCACA	55659	0.13243328160982376	No Hit
TGGAAGACTAGTGATTTTGTTGTTAGATCGGAAGAG	52403	0.1246860571731363	No Hit

AGATCGGAAGAGCACACGTCTGAACTCCAGTCACAG	51763	0.12316326121506507	TruSeq Adapter, Index 2 (97% over 35bp)
TGACTAGATCCACACTCATCTAGATCGGAAGAGCAC	42659	0.10150148871150166	No Hit

Adapter Content

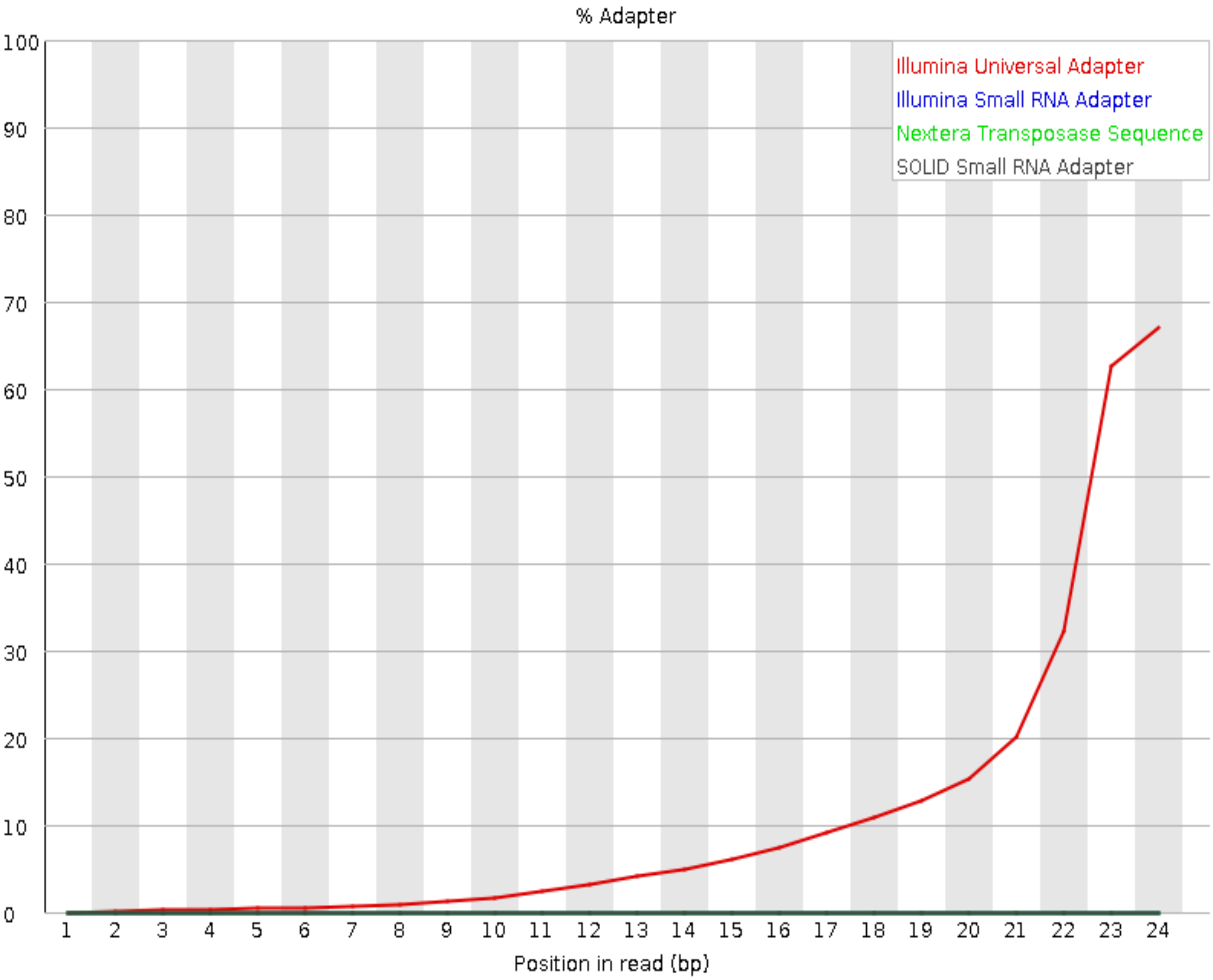

Kmer Content

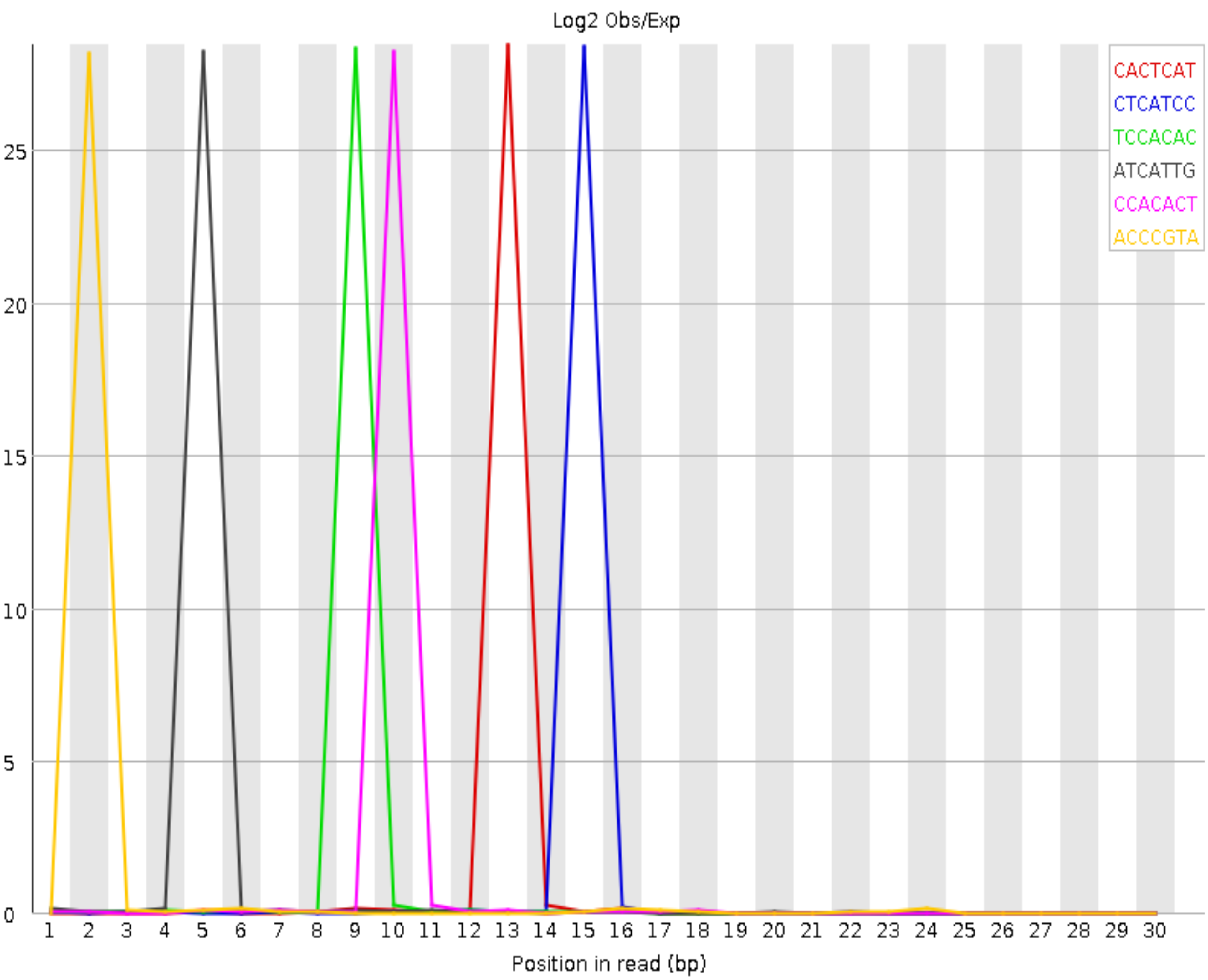

Sequence	Count	PValue	Obs/Exp Max	Max Obs/Exp Position
CACTCAT	51490	0.0	28.42117	13
CTCATCC	39630	0.0	28.383307	15
TCCACAC	55285	0.0	28.327763	9
ATCATTG	34330	0.0	28.230392	5
CCACACT	53890	0.0	28.215288	10
ACCCGTA	31435	0.0	28.201769	2
AACCCGT	31285	0.0	28.192446	1
ATGTAAA	85990	0.0	28.14395	5
GATCATT	35965	0.0	28.027193	4
AATGTAA	87970	0.0	27.982782	4
GATCCAC	57450	0.0	27.948393	7
AGTATGG	80465	0.0	27.800564	14
GAATGTA	90220	0.0	27.774414	3

ACTCATC	50805	0.0	27.75345	14
TGTAAAG	87865	0.0	27.644192	6
ATCCACA	57915	0.0	27.511253	8
GGAATGT	91155	0.0	27.497822	2
TAATACT	16100	0.0	27.461235	1
AAGTATG	84805	0.0	27.380571	13
CAGGTAA	16515	0.0	27.347359	10

Report

Fri 3 Apr 2015
ISE6-U4_S2_L002_R2_001.fastq.gz

Summary

- Basic Statistics
- Per base sequence quality
- Per tile sequence quality
- Per sequence quality scores
- Per base sequence content
- Per sequence GC content
- Per base N content
- Sequence Length Distribution
- Sequence Duplication Levels
- Overrepresented sequences
- Adapter Content
- Kmer Content

Basic Statistics

Measure	Value
Filename	ISE6-U4_S2_L002_R2_001.fastq.gz
File type	Conventional base calls
Encoding	Sanger / Illumina 1.9
Total Sequences	2751362
Sequences flagged as poor quality	0
Sequence length	36
%GC	51

Per base sequence quality

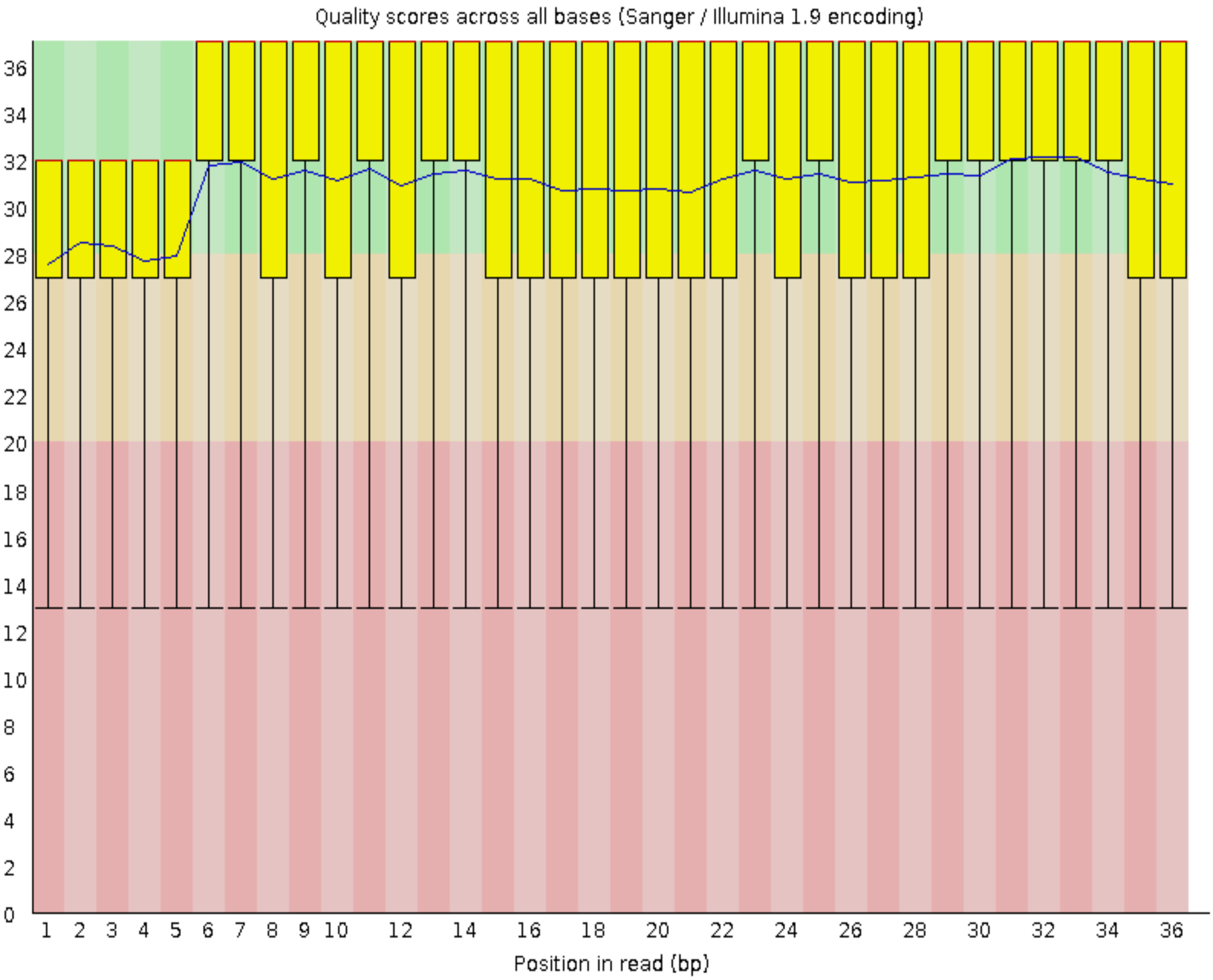

Per tile sequence quality

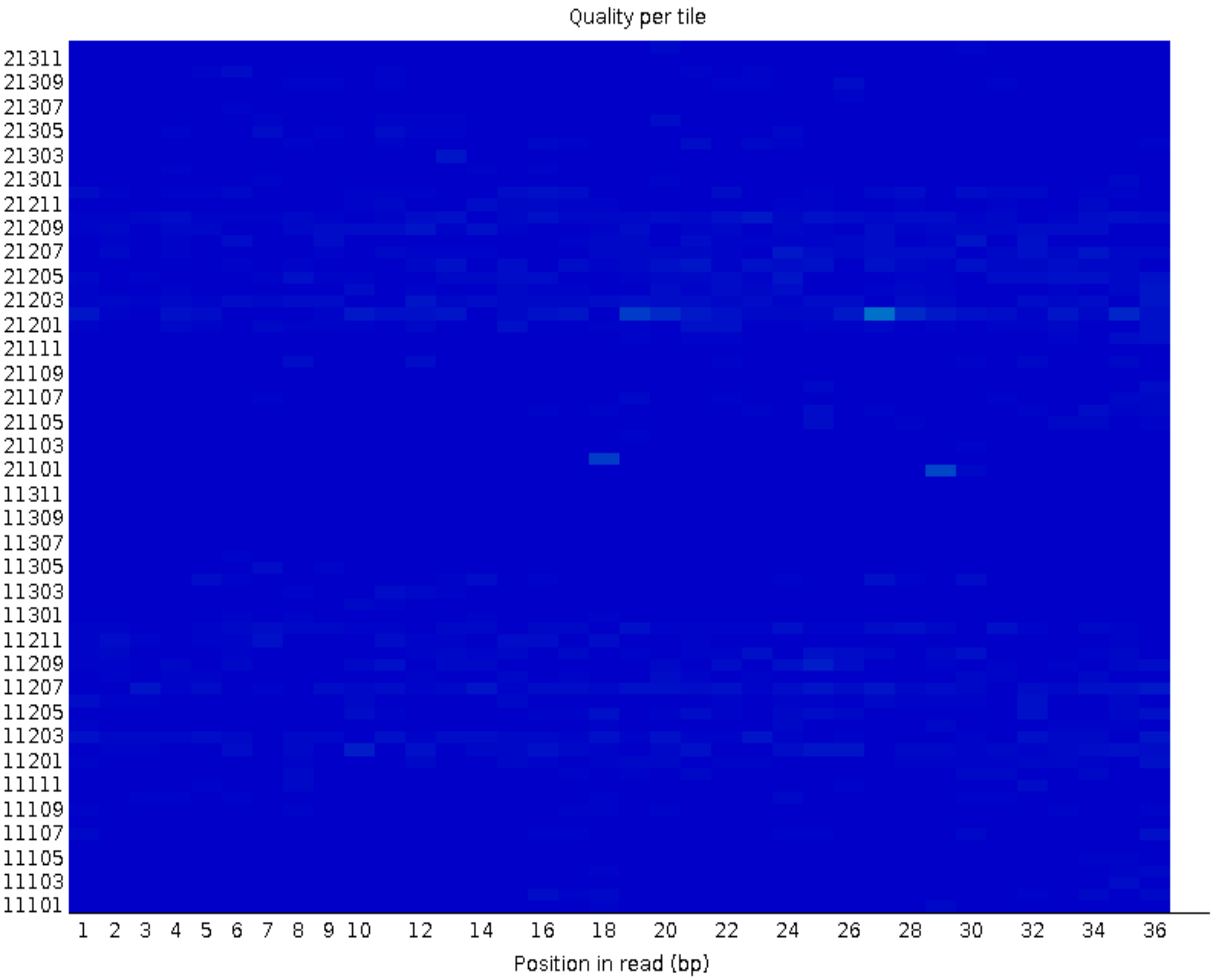

Per sequence quality scores

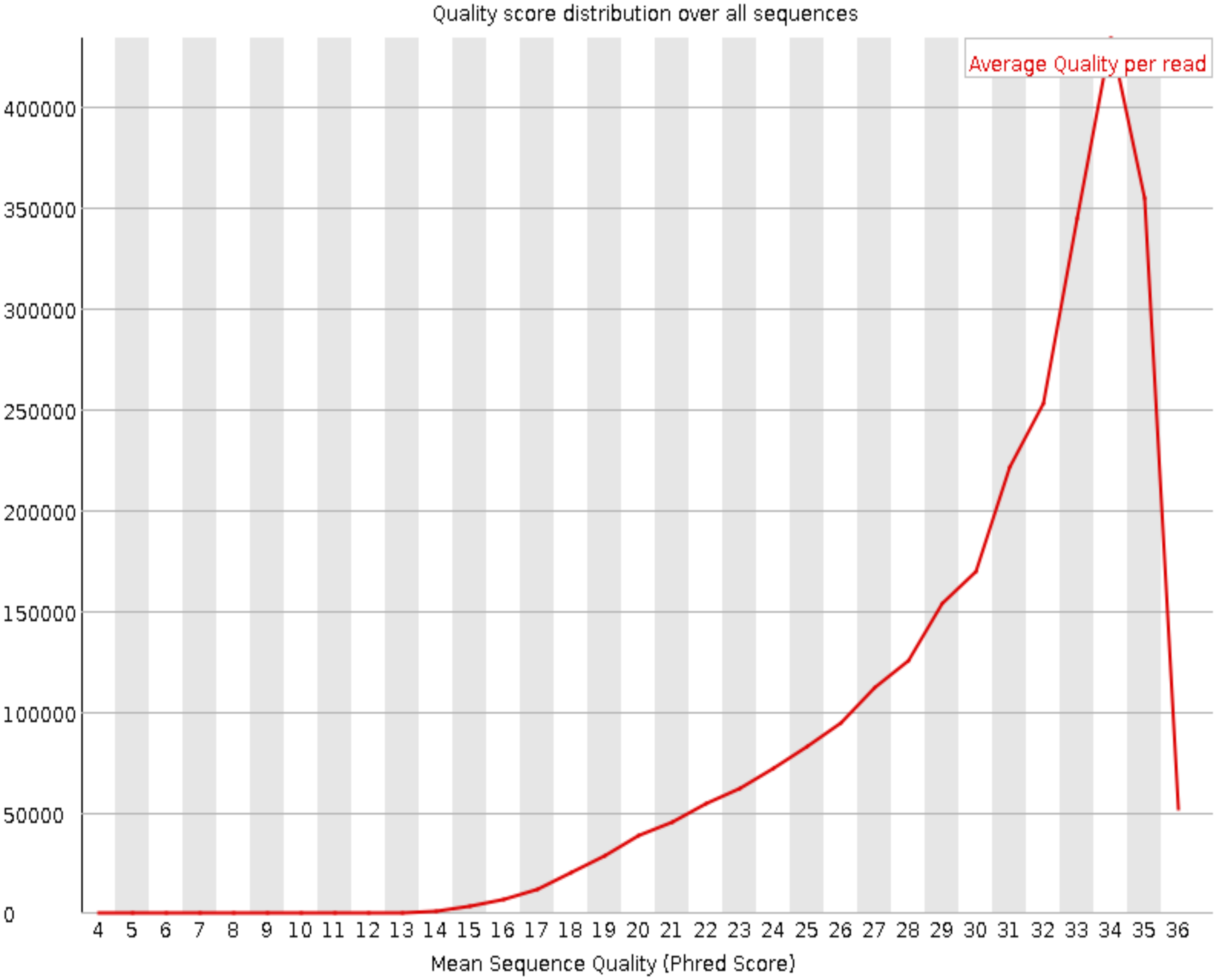

Per base sequence content

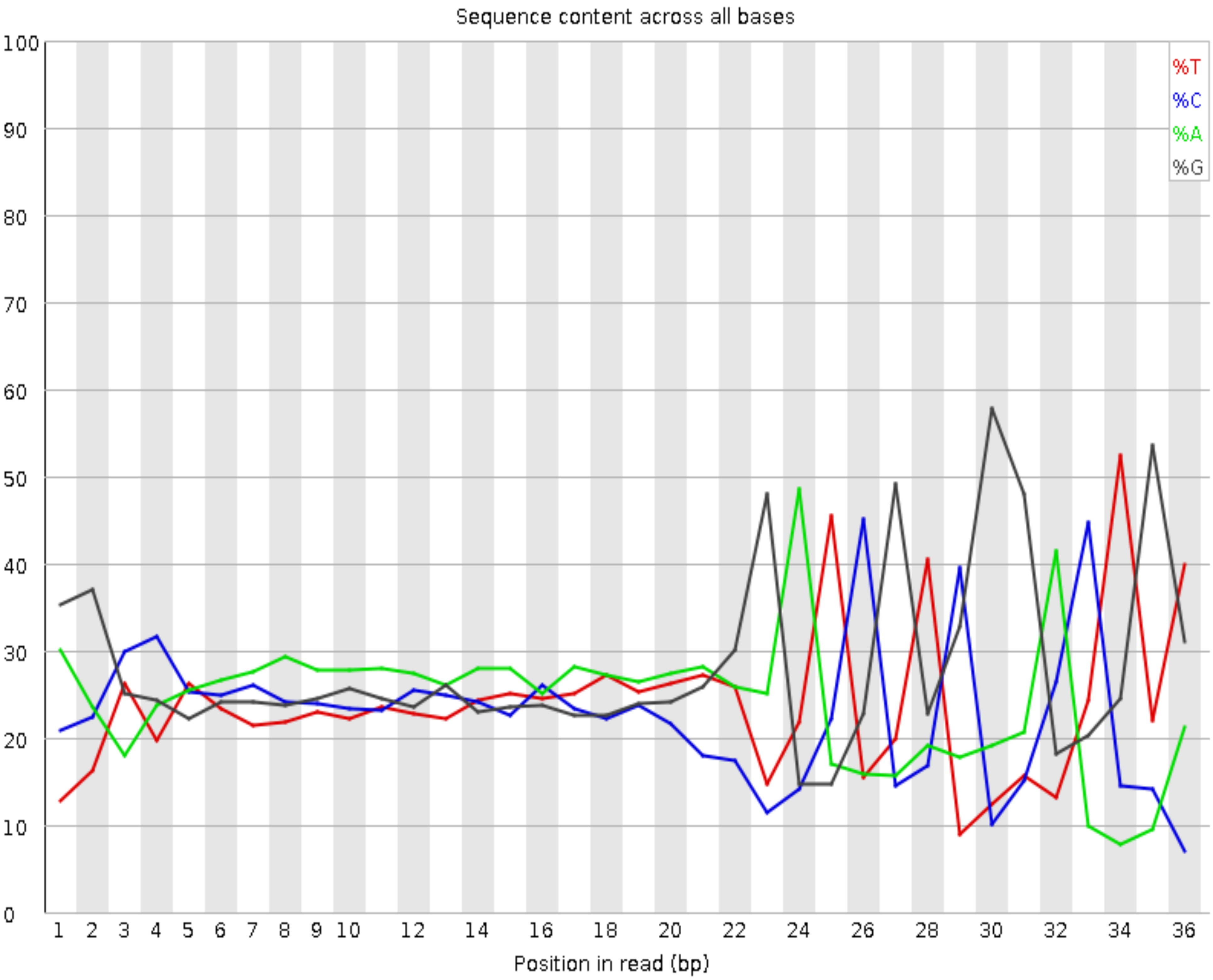

Per sequence GC content

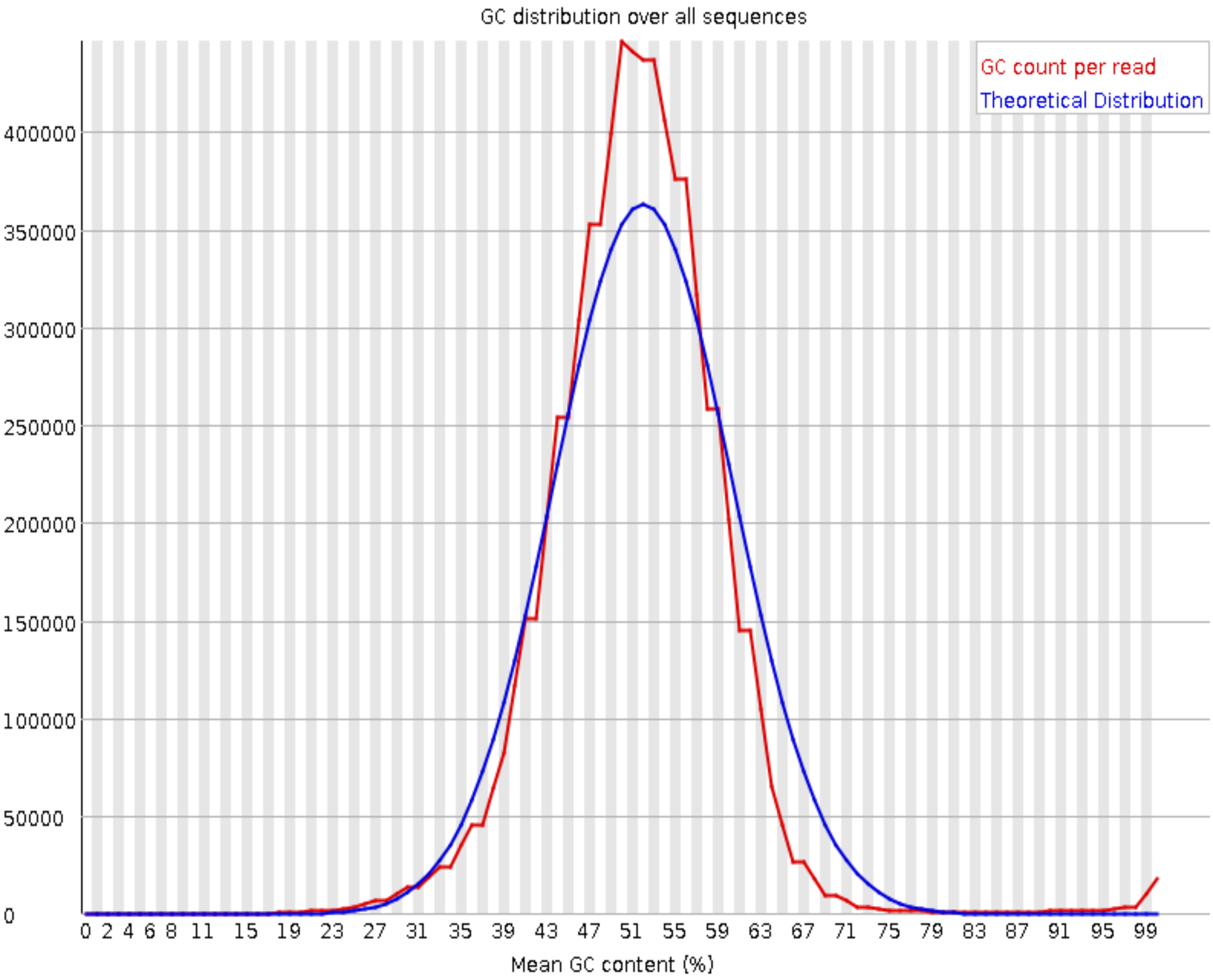

Per base N content

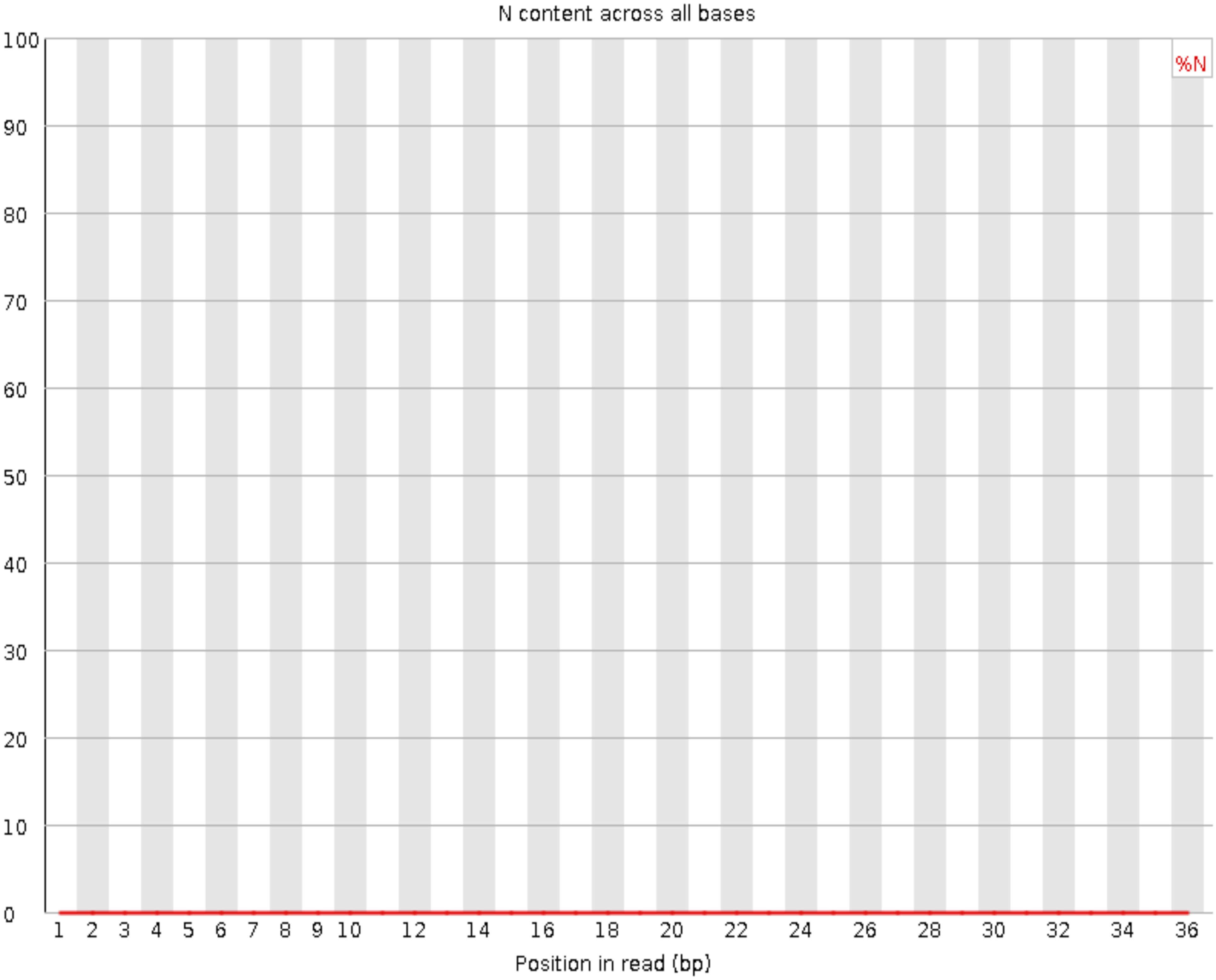

Sequence Length Distribution

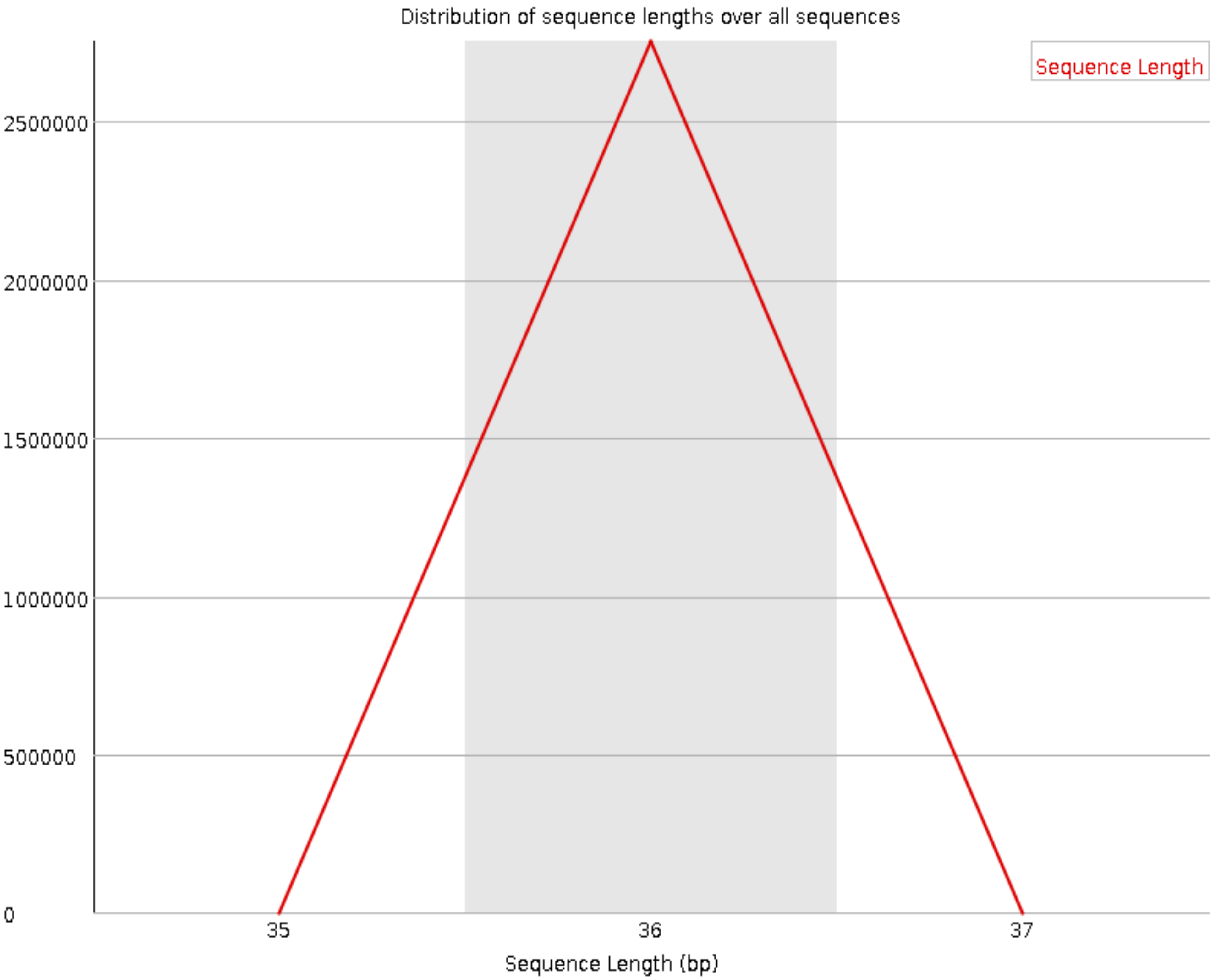

Sequence Duplication Levels

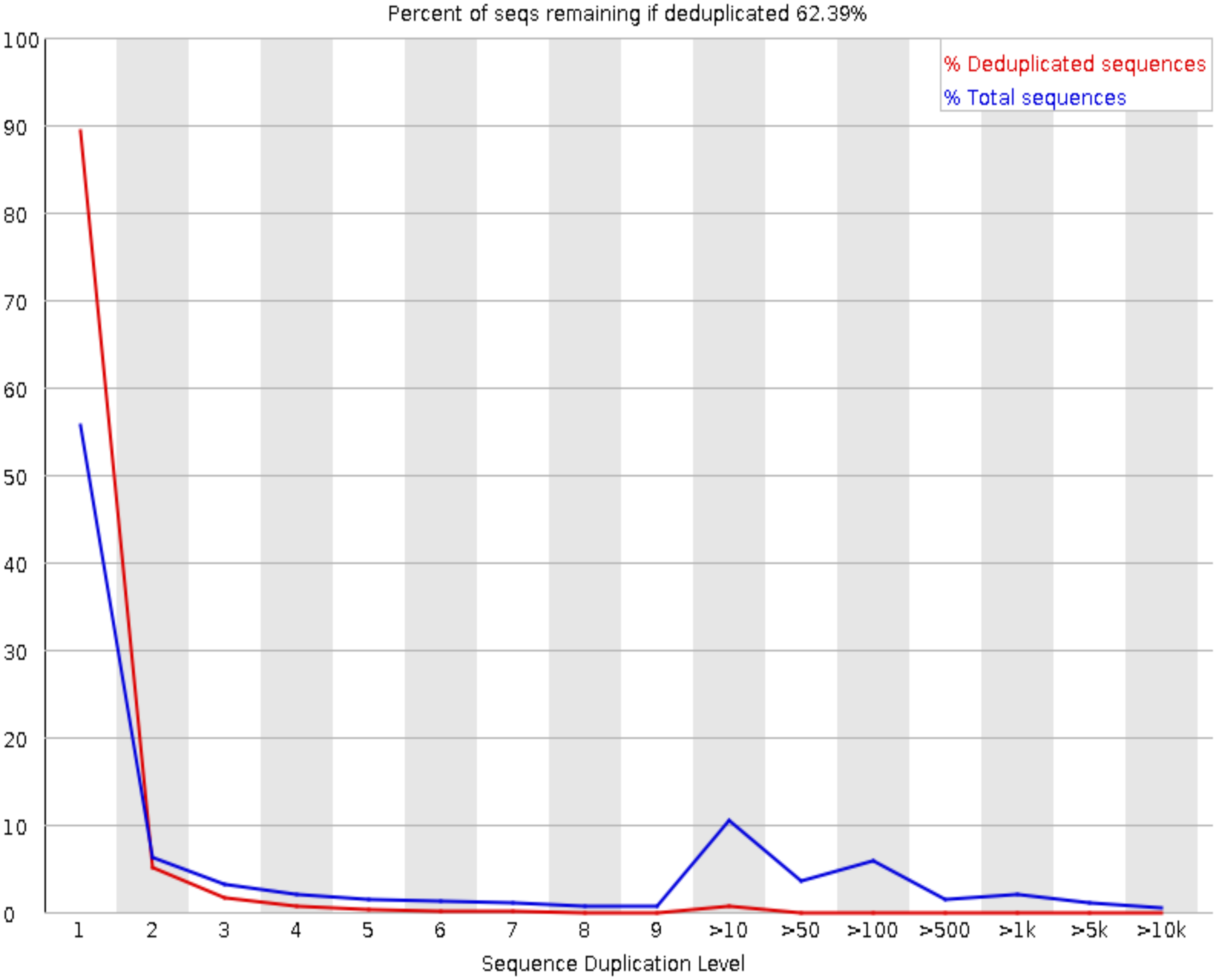

Overrepresented sequences

Sequence	Count	Percentage	Possible Source
GGGGGGGGGGGGGGGGGGGGGGGGGGGGGGGGGGGG	16729	0.6080261339656504	No Hit
GACATCTTTACCTGACAGTATTAGATCGTCGGACTG	9899	0.3597854444453329	No Hit
AGGCTGAACTTCACAGCTCGAAGAAAGAGATCGTCG	8147	0.2961078912916584З	No Hit
ACAAATTCGGATCTACAGGGTAGATCGTCGGACTGT	7937	0.28847530786570436	No Hit
TGGATGAGTGTGGATCTAGTCAGATCGTCGGACTGT	5792	0.21051392001488717	No Hit
AATCAGCTTTCACAATGATCTCAGATCGTCGGACTG	3914	0.14225681680564026	No Hit
TGGTTCCGACACCGGGAATCGAGATCGTCGGACTGT	2906	0.10562041636106045	No Hit
CGTACACAGCAGGATCTTAGTTGATCGTCGGACTGT	2866	0.10416659094659299	No Hit
AGTTATGAATCATAGCTTCCTAGATCGTCGGACTGT	2855	0.10376678895761444	No Hit

AGGCTTGACGTCAAACTTTATTAGGCCGGATCGTCG	2774	0.10082279249331787	No Hit

Adapter Content

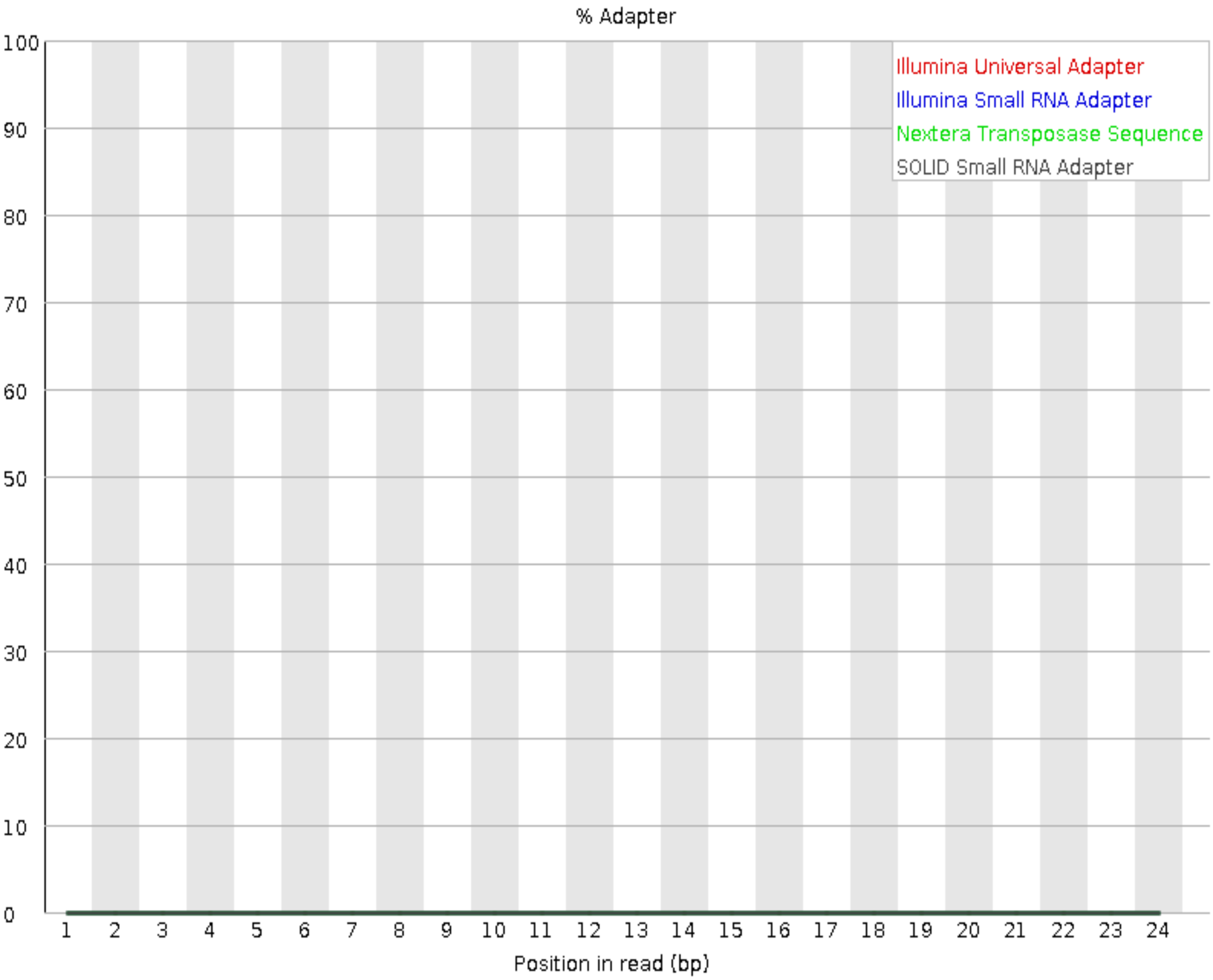

Kmer Content

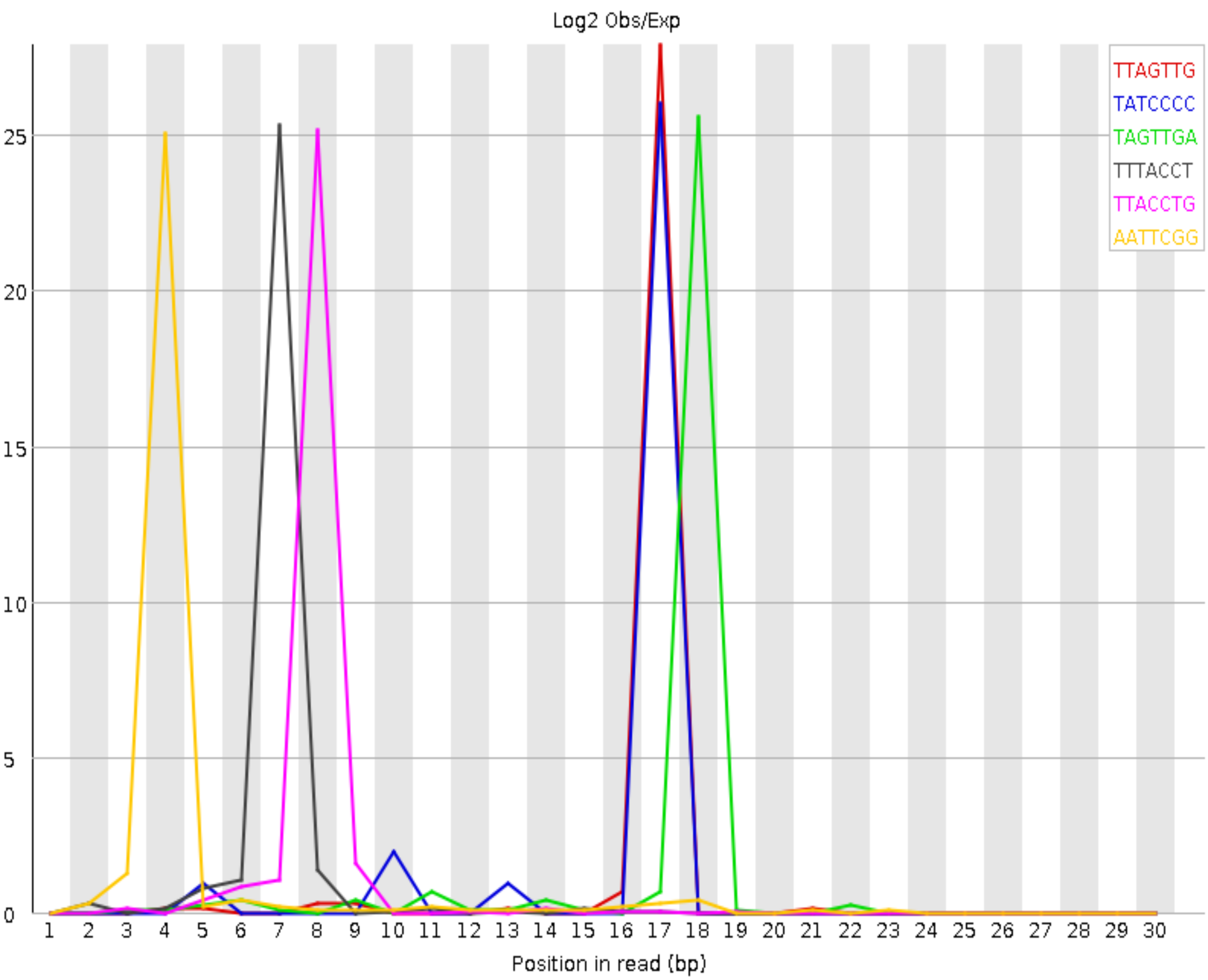

Sequence	Count	PValue	Obs/Exp Max	Max Obs/Exp Position
TTAGTTG	845	0.0	27.869873	17
TATCCCC	150	0.0	26.000048	17
TAGTTGA	1015	0.0	25.567478	18
TTTACCT	1610	0.0	25.338438	7
TTACCTG	1650	0.0	25.178204	8
AATTCGG	1275	0.0	25.054316	4
CAGGGTA	1200	0.0	24.751846	16
CTTTACC	1630	0.0	24.750597	6
AGGGTAG	1235	0.0	24.413	17
AAATTCG	1295	0.0	24.20414	3
GGCTGAA	1375	0.0	23.99568	2
CACCGGG	645	0.0	23.953533	10
ACAGGGT	1305	0.0	23.910696	15

TCTTTAC	1820	0.0	23.814396	5
AGTATTA	1775	0.0	23.746521	17
TGAACTT	1275	0.0	23.525179	5
ATTCGGA	1400	0.0	23.460066	5
GGGTAGA	1340	0.0	23.284472	18
CTGAACT	1280	0.0	23.198952	4
ACCTGAC	1860	0.0	23.145203	10

Produced by FastQC (version 0.11.3)
